# Effects of the Norfolk diabetes prevention lifestyle intervention (NDPS) on glycaemic control in screen-detected type 2 diabetes: a randomised controlled trial

**DOI:** 10.1186/s12916-021-02053-x

**Published:** 2021-08-19

**Authors:** Michael Sampson, Allan Clark, Max Bachmann, Nikki Garner, Lisa Irvine, Amanda Howe, Colin Greaves, Sara Auckland, Jane Smith, Jeremy Turner, Dave Rea, Gerry Rayman, Ketan Dhatariya, W. Garry John, Garry Barton, Rebecca Usher, Clare Ferns, Melanie Pascale, on behalf of the NDPS group, Sara Auckland, Max Bachmann, Garry Barton, Allan Clark, Ketan Dhatariya, Clare Ferns, Nikki Garner, Colin Greaves, Andy Goldson, Martin Hadley-Brown, Amanda Howe, Lisa Irvine, Garry John, Melanie Pascale, David Rea, Jane Smith, Jeremy Turner Rebecca Usher, Tara Wallace

**Affiliations:** 1 Elsie Bertram Diabetes Centre, Department of Diabetes and Endocrinology, Norfolk and Norwich University Hospital NHS Trust, Colney Lane, Norwich, NR4 7UY UK; 2 Norwich Medical School, University of East Anglia, Norwich, UK; 3 School of Sport, Exercise & Rehabilitation Sciences, University of Birmingham, Birmingham, UK; 4 University of Exeter Medical School, College of Medicine & Health, University of Exeter, Exeter, UK; 5 Department of Diabetes and Endocrinology, Ipswich General Hospital NHS Trust, Ipswich, UK; 6 Department Clinical Biochemistry, Norfolk and Norwich University Hospital NHS Trust, Norwich, UK

**Keywords:** Type 2 diabetes, Screen detected, Lifestyle Intervention, Glycaemic control, Diabetes Prevention Mentors, Peer support

## Abstract

**Background:**

The purpose of this trial was to test if the Norfolk Diabetes Prevention Study (NDPS) lifestyle intervention, recently shown to reduce the incidence of type 2 diabetes in high-risk groups, also improved glycaemic control in people with newly diagnosed screen-detected type 2 diabetes.

**Methods:**

We screened 12,778 participants at high risk of type 2 diabetes using a fasting plasma glucose and glycosylated haemoglobin (HbA1c). People with screen-detected type 2 diabetes were randomised in a parallel, three-arm, controlled trial with up to 46 months of follow-up, with a control arm (CON), a group-based lifestyle intervention of 6 core and up to 15 maintenance sessions (INT), or the same intervention with additional support from volunteers with type 2 diabetes trained to co-deliver the lifestyle intervention (INT-DPM). The pre-specified primary end point was mean HbA1c compared between groups at 12 months.

**Results:**

We randomised 432 participants (CON 149; INT 142; INT-DPM 141) with a mean (SD) age of 63.5 (10.0) years, body mass index (BMI) of 32.4 (6.4) kg/m^2^, and HbA1c of 52.5 (10.2) mmol/mol. The primary outcome of mean HbA1c at 12 months (CON 48.5 (9.1) mmol/mol, INT 46.5 (8.1) mmol/mol, and INT-DPM 45.6 (6.0) mmol/mol) was significantly lower in the INT-DPM arm compared to CON (adjusted difference −2.57 mmol/mol; 95% CI −4.5, −0.6; *p* = 0.007) but not significantly different between the INT-DPM and INT arms (−0.55 mmol/mol; 95% CI −2.46, 1.35; *p* = 0.57), or INT vs CON arms (−2.14 mmol/mol; 95% CI −4.33, 0.05; *p* = 0.07). Subgroup analyses showed the intervention had greater effect in participants < 65 years old (difference in mean HbA1c compared to CON −4.76 mmol/mol; 95% CI −7.75, −1.78 mmol/mol) than in older participants (−0.46 mmol/mol; 95% CI −2.67, 1.75; interaction *p* = 0.02). This effect was most significant in the INT-DPM arm (−6.01 mmol/mol; 95% CI −9.56, −2.46 age < 65 years old and −0.22 mmol/mol; 95% CI −2.7, 2.25; aged > 65 years old; *p* = 0.007). The use of oral hypoglycaemic medication was associated with a significantly lower mean HbA1c but only within the INT-DPM arm compared to CON (−7.0 mmol/mol; 95% CI −11.5, −2.5; *p* = 0.003).

**Conclusion:**

The NDPS lifestyle intervention significantly improved glycaemic control after 12 months in people with screen-detected type 2 diabetes when supported by trained peer mentors with type 2 diabetes, particularly those receiving oral hypoglycaemics and those under 65 years old. The effect size was modest, however, and not sustained at 24 months.

**Trial registration:**

ISRCTN34805606. Retrospectively registered 14.4.16

**Supplementary Information:**

The online version contains supplementary material available at 10.1186/s12916-021-02053-x.

## Introduction

Nearly half a billion people are living with diabetes worldwide, with more than 4 million in the UK [1–3], and we need effective lifestyle modification interventions to prevent type 2 diabetes and improve clinical outcomes [4–11]. In the UK, national policy is that people in a ‘high risk of Type 2 diabetes’ glycaemic category should be offered a diet and lifestyle intervention [4]. In parallel, UK policy recommends people with newly diagnosed type 2 diabetes should have access to structured education, with self-management advice, an emphasis on diet and lifestyle modification, delivered by trained educators, and with a theory-driven, quality-assured, and evidence-based structured curriculum [7]. A lifestyle intervention that was found to be effective in both type 2 diabetes prevention and in improving glycaemic control in people with type 2 diabetes would be an attractive choice for clinicians and policy makers. This seems intuitively likely, as the key elements of preventive lifestyle interventions (increased physical activity, dietary modification, and weight loss targets) have modest but statistically significant effects on glycaemic control in type 2 diabetes [8–11]. However, the few lifestyle interventions shown to reduce the risk of type 2 diabetes in current high risk of diabetes glycaemic categories have not been tested as an intervention in people with type 2 diabetes.

The increasing clinical workload in type 2 diabetes management has driven a search for better and less expensive workforce models, including the use of lay volunteers. Small or observational studies have commonly shown benefits from such support in people with type 2 diabetes [12–18]. Shared life experience may help to support the development of motivation for lifestyle change, and ‘modelling’ of behaviours by credible others is a recognised behaviour change technique [19, 20]. People with type 2 diabetes themselves are an obvious choice as true peer supporters since they are demographically similar to the target group, share a common experience of being diabetes aware, and face the same lifestyle challenges. However, the value of using lay people with type 2 diabetes themselves to work in this role alongside health care professionals has not been tested in a large clinical trial.

We recently reported results from the Norfolk Diabetes Prevention Study (NDPS), a clinical trial testing the effectiveness of a group-delivered diet and lifestyle intervention, with or without additional support from trained peer volunteers with type 2 diabetes themselves, to prevent type 2 diabetes in high-risk groups [21]. The intervention was effective in reducing the risk of incident type 2 diabetes, by 40–48% over 2 years [21]. The NDPS screening programme also identified many people with a new diagnosis of type 2 diabetes [21–24]. We hypothesised that the NDPS lifestyle intervention would improve glycaemic control in these people with screen-detected type 2 diabetes.

## Methods

### Study design

NDPS was a research programme conducted between 2011 and 2018 in the East of England (UK National Institute for Health Research NIHR RP PG 0109 –10013). The programme protocol, baseline characteristics of the screened population, and results from the main prevention trial are published [21–24], and the initial protocol and final statistical analysis plan (SAP) are available as Additional files 1 and 2. The NDPS screening programme [21–24] was intended to detect people with a ‘high risk of Type 2 diabetes’ glycaemic category for entry into a prevention trial, but also detected people with newly diagnosed, ‘screen detected’ type 2 diabetes. The parallel, three-arm randomised controlled trial reported here ran alongside the prevention trial and examined the effects of the NDPS lifestyle intervention, with or without support from trained peer volunteers with type 2 diabetes (DPM) [21–24] on glycaemic control (HbA1c) in people with screen-detected type 2 diabetes, with pre-specified end points at 12 and 24 months [24]. The CONSORT checklist is available as Additional file 3.

### Recruitment

To identify high-risk participants eligible for screening, we contacted 194 primary care practices in the East of England and 135 (70%) collaborated. Based on searches of computerised patient databases in each practice, we invited all individuals without known diabetes in these practices who (a) were aged ≥ 40 years and with a recorded body mass index (BMI) ≥ 30 kg/m^2^ or (b) were aged ≥ 40 years and with a BMI ≥ 25 kg/m^2^ and a recorded first degree family history of type 2 diabetes, or a history of coronary artery disease, or gestational diabetes or (c) were recorded in any previous high risk of type 2 diabetes glycaemic category [24].

#### Screening

Potential participants were initially screened with fasting plasma glucose and venous HbA1c measurements, and biometric and medical history data were collected [24] in 8 screening sites across the East of England. Participants with an eligible glycaemic category on initial testing had repeated testing a median 40 days (interquartile range 27–69 days) later [21–24]. Trial randomisation was offered if participants met the inclusion criteria described below. The first screening appointment was August 22, 2011, and last March 24, 2017.

### Inclusion criteria

Eligibility for randomisation into this trial was initially for participants with fasting plasma glucose ≥ 7.0 mmol/l on paired baseline samples. In light of international changes in diabetes diagnostic criteria during the programme and UK national policy changes [4, 25–28], we also then enrolled screened participants with paired HbA1c ≥ 48 mmol/mol for the diagnosis of type 2 diabetes from May 2014 [21–24]. In the initial years of this programme (end 2011–2013), there was still uncertainty about the clinical adoption of HbA1c as a test for the diagnosis of type 2 diabetes in normal UK practice, compared to data obtained during a standard 75-g oral glucose tolerance test (OGTT) [28, 29]. To exclude an OGTT-based diagnosis of type 2 diabetes (paired 2-h plasma glucose > 11.1 mmol/l) and incorrect randomisation into the NDPS diabetes prevention trial [21, 24], we undertook an OGTT in higher risk screened participants (fasting plasma glucose ≥ 6.1–< 7.0 mmol/l and HbA1c > 42 mmol/mol) and recruited into the trial reported here those with paired OGTT 2-h glucose values of both > 11.1 mmol/mol. Participants who declined a repeat OGTT after an initial diagnostic value were also offered randomisation into this trial if they had paired baseline HbA1c ≥ 48 mmol/mol [24–29]. Participants with a single abnormal OGTT were also accepted if they had extreme or symptomatic hyperglycaemia.

#### Ethical issues

Ethical approval was obtained from the National Research Ethics Service (NRES), Essex 1 Research Ethics Committee (10/H0301/55; January 13, 2011), and all participants gave written informed consent. Participants with newly diagnosed type 2 diabetes were informed of their diagnosis and offered discussion with the study team and CI. Each participants’ primary care clinician was informed of the diagnosis, and normal diabetes care and management was provided outside the trial protocol by their usual clinician.

#### Randomisation and consent

We randomised participants in parallel with the screening programme using a rolling recruitment approach with screening and randomisation continuing from August 2011 until 6.4.2017. This allowed each participant to reach a 12 months minimum and 46 months maximum follow-up within the trial. Randomisation was conducted automatically using a dedicated algorithm in the trial data management system. The randomisation mechanism consisted of a pre-prepared random list of codes (for the intervention and control groups) stored in the trial database, and randomisation to groups was in a 1:1:1 ratio [24]. Randomisation enrollment was undertaken by NDPS diabetes prevention facilitators (DPF).

#### Interventions

The NDPS intervention is described in detail elsewhere [21, 24]. Eligible participants were randomised into a control arm (CON) who received no trial intervention, an intervention arm (INT) who received the lifestyle intervention (INT), or an intervention arm who received the same intervention, but with additional telephone support from peer volunteer diabetes prevention mentors (INT-DPM).

##### Control (CON) group

The CON arm participants attended a single, 2-h, group-based session delivered by a DPF [24]. Discussions included a presentation and written information on type 2 diabetes at diagnosis, and the impact of lifestyle modification, in line with then current local NHS clinical policy. CON arm participants did not then receive any additional in trial lifestyle modification advice.

##### Intervention (INT) group

The intervention comprised six, 2-h educational group sessions for the first 12 weeks, followed by *up to* 15 maintenance sessions 8 weeks apart from month 4 onwards. Maintenance sessions were 2.5 h in duration and included 90 min of discussion-based activities to review progress and action plans and discuss key topics relating to lifestyle change and maintenance. Every maintenance session also included a 50-min supervised physical activity/muscle-strengthening exercise session. The maximum possible session contact time per participant was up to 49.5 h. Sessions contained no more than 15 participants each. Participant groups included people with a ‘high risk of Type 2 diabetes’ glycaemic category in the prevention trial [21, 24] as well as people with screen-detected type 2 diabetes, and participants were not informed of each other’s glycaemic category. The INT arm intervention was delivered by DPF alone.

##### Intervention and mentor (INT-DPM) group

Participants randomised to the INT-DPM arm received the same intervention as the INT group as described above (six ‘core’ education sessions with a maximum number of 15 maintenance sessions) but in addition received up to 18 individual motivational telephone calls scheduled between intervention sessions [21, 24]. DPMs were each assigned up to seven participants and telephone contacts were monthly for the first 3 months and then every 2 months. During these contacts, the DPM and participants discussed progress, goal achievement, action planning, and barriers to coping. INT-DPM participants therefore received a contact from the study at least every 4 weeks. The INT-DPM arm intervention was delivered jointly by DPF and trained volunteers with type 2 diabetes themselves (DPM) [22, 24]. DPMs were from a range of professional backgrounds and trained to co-deliver the lifestyle intervention [22].

The intervention was based on the Process Model for Lifestyle Behaviour Change [30, 31] and aimed to support the maintenance of changes in physical activity and diet, using patient-centred counselling techniques to encourage decision-making about behaviour change, increase motivation to change, engage social support, and aid individually tailored goal setting, action planning, self-monitoring, and support problem solving [21–24]. Behaviour change targets were set by participants, who were encouraged to think about (and presented with the health benefits of) achieving 7% weight loss if BMI was > 30 kg/m^2^, achieving 150 min per week of moderate-intensity physical activity over 5 days or more, undertaking 2–3 sessions of muscle-strengthening exercise per week, and reducing intake of total and saturated fat. Participants who had a BMI under 30 kg/m^2^ were not given a suggested weight loss target but were advised to work towards achieving a BMI within a healthy range and that any movement towards that range would be beneficial. Participants in the two intervention groups were additionally given a pedometer as a motivational tool to encourage an increase in activity. These data were not collected for analysis but allowed the participants to monitor and self-regulate their exercise behaviour. Pedometer data were discussed as part of participants’ progress reviews within the intervention sessions and DPM telephone calls.

All trial participants received normal clinical diabetes care from their existing primary care team in line with normal clinical practice and uniform glycaemic targets [32], and none received a structured lifestyle intervention from other sources in parallel with the NDPS intervention. Intervention fidelity was assessed by audio-recording all intervention sessions and a scoring checklist was applied to a sample of these sessions by independent observers. DPFs were required to complete a checklist at the end of every session reporting on the perceived levels of fidelity reached [24]. The completion rate for the DPF-completed checklists was 91.7% and these data will be reported separately.

### DPM training and characteristics

A full description of the DPM training and characteristics has been published [22]. We invited 9951 people with type 2 diabetes to become DPM. Four hundred twenty-seven (4.3%) individuals responded, 356 (83.3%) were interviewed by phone, and 131 (36.8%) were interviewed face to face. One hundred four (79%) were appointed to the role (mean age 62 years, 55% [*n* = 57] male), volunteering for a total of 2895 months, and made 6879 telephone calls to randomised participants in the full NDPS programme [24]. Seventy-five (72%) DPM volunteered for at least 6 months and fifty-four (52%) for at least 1 year. The study-specific training programme was designed as a standardised training programme to allow for exact replication in future cohorts. Group training seminars were delivered over 7 weeks (one per week) allowing time for self-reflection and reading between seminars. Each seminar lasted 2.5 h. The training had two aims: to provide up-to-date information on physical activity, diet, pre-diabetes, and lifestyle-related areas and secondly to undertake practice role play work to allow for the development of the key skills required for the role.

#### Outcome measures

The primary pre-specified outcome was mean HbA1c at 12 months, and pre-specified secondary outcomes included biometric, biochemical, behavioural, and quality of life measures, which are described elsewhere [24]. The Quality and Outcomes Framework (QOF) is a UK primary care incentive scheme for many conditions, and for diabetes, the main QOF metric is simply the percentage of people in a primary care diabetes population achieving agreed target glycaemic levels [32]. We also report QOF attainment at baseline and at 12 months by trial arm as a primary end point. Weight, body mass index (BMI), body fat mass (kg), and visceral fat were measured using a Tanita body fat composition analyser (TANITA - Hoogoorddreef, 1101 BE, Amsterdam, The Netherlands; Model BC-420 MA), with a possible visceral fat score of 1–59, with a rating between 1 and 12 indicating a healthy level of visceral fat and 12–59 indicating excessive level of visceral fat. Fasting plasma glucose was measured using the hexokinase/G-6-PDH method (Architect c8000: Abbott, Maidenhead, UK). HbA1c was measured using Affinity high-performance liquid chromatography (Hb9210: Menarini Diagnostics Ltd., Wokingham, UK). Fasting plasma insulin was measured on the Siemens Immulite 2000 XPI (Siemens Healthcare Ltd, Frimley, Camberley, Surrey; GU16 8QD), and homeostasis model assessment (HOMA) of insulin sensitivity and beta cell function were calculated [33]. Physical activity was self-reported using the short form International Physical Activity Questionnaire (IPAQ) [34, 35] which gathers information on the intensity (vigorous, moderate, or light) and duration of a range of activities engaged in over the course of the last week and also the amount of sedentary activity measured in hours each day. Physical activity outputs were reported in categories (low, moderate, high) and converted to Metabolic Equivalents (METS) to express the intensity of physical activity [34, 35]. Resistance activity was assessed via a study-specific two-item questionnaire measuring the type of resistance activity, the number of minutes per day, and the number of days per week the participant engaged in resistance activity. This questionnaire was measured alongside the IPAQ. In the NDPS programme, a small number of accelerometers were used between all randomised participants in the main prevention trial (24) and this trial. If an accelerometer was available for assignment, it was provided to the next attending participant but the data was only measured in a very small subsample of participants in this trial. This use of accelerometers in only a subset of participants is specified in protocol (Additional File; Protocol p41), but there are insufficient data from this small accelerometry dataset to be of value, and are nor reported here. Dietary behaviours related to fat and fibre intake were assessed using a Diet Behaviour Questionnaire (DBQ) adapted from an existing dietary questionnaire [36]. Well-being was assessed by the WBQ-12 questionnaire, which captures general well-being, including negative well-being, energy, and positive well-being [37]. Health-related quality of life was measured using the EuroQol EQ-5D [38, 39]. Diabetes quality of life was assessed using the Audit of Diabetes-Dependent Quality of Life (ADDQol) questionnaire [39]. Measures of deprivation for each participant were derived from their postcode and published indices of deprivation [40]. The Diabetes Treatment Satisfaction Questionnaire (DTSQ) [41] and diabetes management self-efficacy scale (DMSES) [42] were used at follow-up time points [24]. Participant medication use was ascertained using data collected from a health resources use (HRU) questionnaire, the trial case report form (CRF) data collection, and direct interview with participants by programme staff at trial end to ascertain medication use at 12 months.

#### Health economic analysis

A within-trial analysis estimated the cost-effectiveness of the intervention (with and without DPM), compared to usual care. To estimate costs (from a UK National Health Service (NHS) perspective at 2016-2017 prices), those who delivered the intervention recorded the resource use (time inputs) associated with the training, education, and maintenance sessions, plus ongoing supervision/support. Additionally, all participants were asked to complete a self-report health service use questionnaire at baseline, 6-, 12-, and 24-month time points [43]. Unit costs were assigned to each item of resource use [43–45]. Additional DPM costs included training, a telephone charge cost for each call, and DPM supervision, as well as a single honorarium payment of £350 for each DPM. Effectiveness was estimated based on the quality-adjusted life year (QALY) scores which were derived from the EQ-5D-3L [45–47]. Incremental costs per QALY were estimated over a 24-month follow-up period, where costs and QALYs incurred after 12 months were discounted at 3.5% and multiple imputation was used to estimate missing data [48]. Bivariate regression analysis [49, 50] was undertaken and the incremental cost-effectiveness ratio (ICER: mean incremental cost/mean incremental effect) was estimated.

### Statistical analysis and power estimates

The primary statistical analysis compared mean HbA1c between the three trial arms using observed data. The comparison was based on a linear regression model with the arm as a fixed effect and adjusted for the baseline value of the outcome. Other continuous outcomes were assessed using the same approach. Binary outcomes were compared using a logistic regression using the same approach. Pre-specified subgroup analyses were conducted by including an interaction between the arm and the subgroup in the regression models separately for sex, age (< 65 vs ≥ 65), deprivation quartile, and BMI quartile. A post hoc analysis of oral hypoglycaemic use, or no reported oral hypoglycaemic use on the primary outcome by trial arm was also undertaken. The sensitivity of the results to missing data was assessed by multiply imputing the missing outcomes for individuals using iterative chain equations. Missing data were assumed to be missing at random due to the reasons provided for drop-out, and that for the 24-month data, the main reason for missingness was that the rolling recruitment strategy meant randomised participants would reach a minimum of 12 months but would not reach 24 months by programme end. In order to control for type-1 error, we pre-specified a restricted analysis to only the 12- and 24-month follow-up data, with 12-month data (365 days from randomisation ± 45 days) selected as the primary end point. We pre-specified this final statistical analysis plan (SAP) in agreement with programme independent data monitoring and ethics committee (DMEC). This SAP (12.10.18) is available as an Additional material file, and antedated programme completion, database lockdown, or any analyses.

Four between-arm comparisons were made: INT-DPM vs INT, INT vs CON, INT-DPM vs CON, and INT combined with INT-DPM vs CON (a post hoc analysis). Within-arm analyses examined the association between intervention exposure (dose) and outcomes with dose defined as follows: For the INT group, the ‘dose of intervention’ attained was defined as LOW (less than 30% attendance at sessions), MODERATE (between 30 and 59% attendance), and HIGH (at least 60% attendance at sessions). For the INT-DPM group, these doses were defined as LOW (less than 30% attendance at sessions regardless of calls connected OR less than 30% of calls connected regardless of attendance at sessions), MODERATE (between 30 and 59% attendance at sessions and more than 30% of calls connected or between 30 and 59% of calls connected and more than 30% attendance at sessions), and HIGH (at least 60% attendance at sessions AND at least 60% of calls connected). Linear regression models were used for the dose-response analysis within each intervention arm. The factors adjusted for were based on a backward elimination algorithm starting with all baseline measures, retaining those that were significant at the 5% level. The baseline measures (Table 1) were considered to be potential confounders of the associations between intervention dose and outcome and included sociodemographic, behavioural, biometric, and generic health variables. These variables were considered as potential confounders because they could either directly influence both intervention uptake and change in HbA1c or glucose (through mechanisms other than the intervention) or be indirect indicators of behavioural and physical health at baseline that could also influence intervention uptake and outcomes. The statistical analysis plan (SAP), sample size estimates, and initial power calculations have been described [24; Additional material File]. The initial power estimates were based on 90% power to detect a mean difference in HbA1c of 0.5% (DCCT aligned HbA1c; 2010) between trial arms, with an initial sample size of *n* = 100 per group.

**Table 1:** Baseline characteristics of control (CON), standard intervention (INT), and intervention with diabetes prevention mentors (INT-DPM) arms

	CON		INT		INT-DPM	
	*n*		*n*		*n*	
Age (years)	149	63.5 (10.0)	142	64.6 (10.1)	141	64.1 (9.9)
Sex (*n*; %)						
Male	149	63 (42.3%)	142	61 (43.0 %)	141	57 (40.4%)
Female		86 (57.7%)		81 (57.0%)		84 (59.6%)
Family history type 2 diabetes (*n*; %)	149	73 (49.0 %)	142	58 (40.8%)	141	63 (44.7%)
Ethnicity						
White	148	91.9	140	96.4	141	92.9
South Asian		1.4		1.4		3.5
Black		1.4		1.4		1.4
Others (%)		5.4		0.7		2.1
Family history cardiovascular disease (*n*; %)		13 (8.7)		22 (15.5)		25 (17.7)
Social deprivation score^a^	149	16.8 (10.6)	142	17.2 (13.7)	141	16.3 (9.5)
Weight (kg)	149	93.3 (19.3)	142	93.0 (17.4)	141	93.7 (19.7)
Body mass index (kg/m^2^)	149	32.4 (6.4)	142	32.4 (5.4)	141	32.8 (6.4)
Waist circumference (cm)	149	108.6 (14.2)	142	108.5(12.5)	141	109.1 (14.2)
Body fat mass (kg)^b^	142	35.9 (9.6)	139	36.5 (9.1)	138	36.2 (8.7)
Visceral fat score^b^	142	15.2 (5.2)	139	15.4 (4.6)	138	16.0 (5.7)
HbA1c (mmol/mol)	149	52.5 (10.2)	142	52.7 (11.3)	141	51.1 (8.1)
HbA1c < 58 mmol/mol (*n*; %)^c^	149	129 (86.6%)	142	124 (87.3%)	141	129 (91.5%)
HbA1c < 86 mmol/mol (*n*; %)^c^	149	147 (98.7%)	142	136 (95.8%)	141	139 (98.6%)
Fasting plasma glucose (mmol/l)	149	7.2 (1.7)	142	7.3 (1.8)	141	7.1 (1.4)
Fasting HDL cholesterol (mmol/l)	140	1.26 (0.3)	136	1.22 (0.3)	139	1.23 (0.36)
Fasting LDL cholesterol (mmol/l)	136	3.2 (1.1)	135	3.1 (1.0)	136	3.1 (1.1)
Fasting plasma insulin (pmol/l)	142	106.3 (65.6)	133	137.1 (107)	138	124.3 (87.1)
HOMA insulin sensitivity (%)^d^	141	70.4 (54.3)	131	55.9 (38.8)	137	59.3 (39.5)
HOMA beta cell function (%)^d^	141	77.2 (36.8)	131	93.3 (57.6)	137	88.6 (48.3)
Physical activity: MET minutes per week^e^	97	2523 (2590)	97	2786 (2861)	96	2786 (2912)
Physical activity: minutes sitting per week^e^	107	445 (258)	106	527 (336)	99	473 (265)
Dietary fibre intake scale^f^	112	2.46 (0.37)	116	2.40 (0.38)	111	2.41 (0.38)
Dietary fat scale^f^	114	2.32 (0.32)	116	2.33 (0.31)	111	2.38 (0.28)
ADD QoL^g^	106	1.41 (0.94)	108	1.17 (1.1)	100	1.1 (1.01)
W-BQ12^g^	106	25.8 (5.7)	102	23.6 (7.5)	98	22.7 (7.7)
EQ-5D^f^	111	0.84 (0.18)	112	0.82 (0.23)	108	0.75 (0.27)

Data are shown as mean and standard deviation (SD) for continuous variables or as *n* (%) for categorical variables. *n* columns show the data available for each variable for each group. ^a^*IMD* Index of Multiple Deprivation mean score. ^b^Body fat by Tanita body composition analyser. ^c^Quality and Outcomes Framework (QOF) glycaemic attainment. ^d^Homeostasis model assessment (HOMA) of baseline insulin sensitivity (S) and beta cell function (B) expressed as percentage of standard reference range. ^e^Physical activity scales derived from international physical activity questionnaire IPAQ. ^f^ Dietary fat and fibre scores based on self-reported Diet Behaviour Questionnaire (DBQ)—higher scale score indicates lower fat intake. ^g^Well-being score (WBQ-12) questionnaire, health-related quality of life score (EQ-5D) questionnaire, and Audit of Diabetes-Dependent Quality of Life (ADDQoL)

## Results

### Participant recruitment and characteristics

**Fig. 1:**
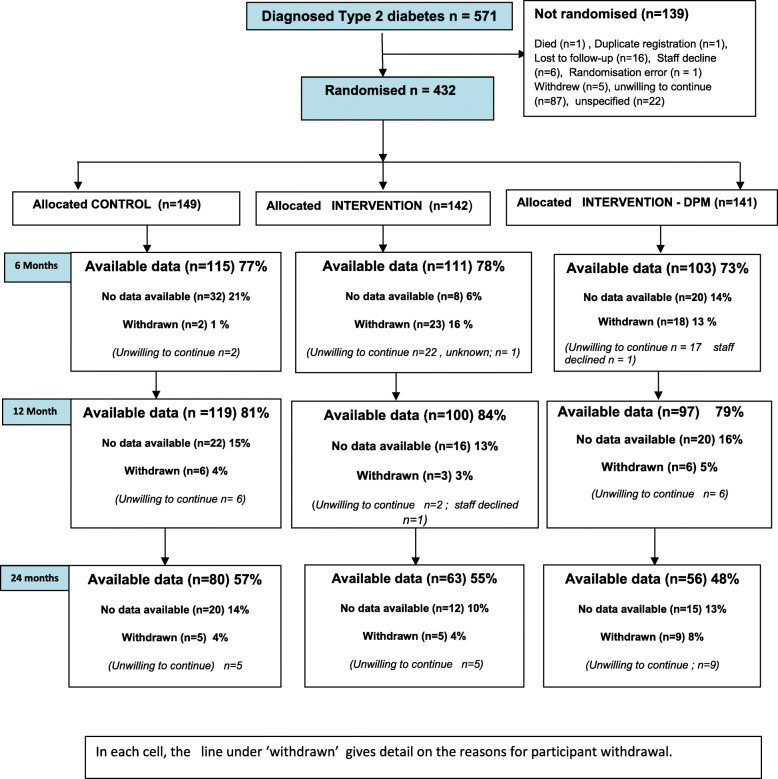
CONSORT diagram: participant flow through trial arms

We invited 141,973 people at increased risk of type 2 diabetes to participate in the screening programme, of whom 12,778 (9.0%) were screened. We detected 571 people with screen-detected type 2 diabetes (4.5%), of whom 432 (75.4%) consented to randomisation into trial arms: CON: *n* = 149, INT: *n* = 142, and INT-DPM: *n* = 141. Baseline characteristics (Table 1) and flow through trial, including loss to follow-up data, and the reasons for participants declining randomisation are shown (Fig. 1).

Of the 432 randomised participants, 74 (17.1%) were diagnosed on the basis of paired fasting plasma glucose measurements ≥ 7.0 mmol/l alone, 150 (34.7%) with paired HbA1c ≥ 48 mmol/mol alone, and 145 (33.6%) met both HbA1c and fasting glucose diagnostic criteria. In the early years of this programme, we randomised 29 (6.7%) with paired baseline 2-h OGTT values > 11.1 mmol/l, and 31 (7.3%) with a single OGTT > 11.1 mmol/l (28 with paired HbA1c > 48 mmol/mol and three with symptomatic hyperglycaemia), and 3 (0.7%) with symptoms and substantial single point hyperglycaemia (fasting plasma glucose 15.4–18.6 and HbA1c 101 to 110 mmol/mol).

All participants had the opportunity to attend all six ‘core’ education sessions allowing a minimum 12-month follow-up, but the trial end date of 31.03.2018 precluded all participants receiving all maintenance sessions (maximum 15) or receive all DPM calls (maximum 18). The median number of the six ‘core’ education sessions attended for both the INT and INT-DPM groups was 5 [interquartile range (IQR) = 2] (mean 4.7 (SD 1.5) INT, and 4.2 (1.9) INT-DPM) for participants who did not withdraw at any point during the programme. Intervention groups combined reported an overall attendance rate for core sessions of 78% (78.1% INT; 77.9% INT-DPM), with 98% of participants attending at least one core education session. Attendance rates and proportions were calculated for participants who did not withdraw during the programme (INT *n* = 89; INT-DPM *n* = 85). Intervention groups combined reported a 43.1% attendance at maintenance session (43.0% INT; 43.1% INT-DPM). The total number of telephone calls made to all participants in the INT-DPM arm in this trial was 1721. The mean percentage of connected calls based on participants’ randomisation date to trial (maximum 18) was 72.6% (SD 0.25), with a median connection rate of 80%.

### Primary outcome

The primary outcome (mean HbA1c) in each group at 12 months was CON 48.5 (9.1) mmol/mol, INT 46.5 (8.1) mmol/mol, and INT-DPM 45.6 (6.0) mmol/mol. The estimate of the difference in mean HbA1c by treatment arms (Table 2) showed the combined intervention arm had a significantly lower mean adjusted HbA1c than CON, and this difference remained significant after imputation analysis (*p* = 0.007). The only significant adjusted difference between arms was in the INT-DPM vs CON comparison (*p* = 0.007). At baseline, nearly all participants in each arm were already achieving the QOF [24] glycaemic targets of HbA1c < 58 or < 86 mmol/mol (Table 1). At 12 months, the equivalent data for the percentage of participants attaining HbA1c < 58 mmol/mol were CON 89.9%, INT 92.1%, and INT-DPM 95.0%, and for participants attaining HbA1c < 86 mmol/mol, the equivalent figures were 99%, 99%, and 100% respectively. There were no important harms or serious adverse events recorded.

**Table 2:** Primary outcome: estimates of difference at 12 months between treatment arms in mean HbA1c (mmol/mol) shown as effect size and 95% confidence interval

Analysis	INT-DPM vs INT	*p*	INT vs CONTROL	*p*	INT- DPM vs CONTROL	*p*	Combined INT vs CONTROL	*p*
**Adjusted**	**−0.55 (−2.46, 1.35)**	**0.57**	**−2.14 (−4.33, 0.05)**	**0.06**	**−2.57 (−4.52,−0.61)**	**0.01**	**−2.38 (−4.08, −0.68)**	**0.01**
**Imputed adjusted**	**−0.83 (−2.82, −1.15)**	**0.41**	**−1.92 (−4.01, 0.18)**	**0.07**	**−2.62 (−4.50, −0.74)**	**0.007**	**−2.30 (−3.96, −0.64)**	**0.007**

*INT*, standard intervention group; *INT-DPM*, intervention group with diabetes prevention mentors (DPM); combined intervention, both intervention groups combined; *CONTROL*, control arm without trial intervention; *Adjusted*, analysis corrected for baseline HbA1c and imputed for missing data. The Combined INT vs CONTROL comparison is a post hoc analysis

### Subgroup analyses

Pre-specified subgroup analyses showed no significant interaction in the primary end point with sex, deprivation score, or BMI (Table 3). There was, however, a significant interaction between age in the INT-DPM vs CON comparison, with greater effects among those aged < 65 years (difference in mean HbA1c −6.0 mmol/mol; 95% CI −9.56, −2.46) compared to those aged over 65 years (−0.22 mmol/mol; 95% CI −2.70, 2.25; interaction *p* = 0.007). There was a trend towards a greater intervention effect size in those in the higher BMI quartiles (Table 4). Post hoc trend analysis showed this trend was significant for the combined group vs control arm comparison (*p* = 0.04), but not for the other comparisons between arms.

**Table 3:** Subgroup analysis: estimate of adjusted difference in mean HbA1c (mmol/mol) at 12 months between treatment arms by age, sex, deprivation score, and body mass index subgroup

	INT-DPM vs INT	***p***	INT vs CONTROL	***p***	INT- DPM vs CONTROL	***p***	Combined intervention vs CONTROL	***p***
**Male**	−0.13 (−2.38, 2.13)	0.42	−3.78 (−6.81, −0.74)	0.1	−3.90 (−6.91, −0.89)	0.29	−3.84 (−6.18, −1.49)	0.09
**Female**	−1.78 (−5.28, 1.72)		0.19 (−3.43, 3.82)		−1.58 (−4.59, 1.43)		−0.70 (−3.56, 2.17)	
**Age < 65 years**	−2.55 (−5.55, 0.46)	0.15	−3.46 (−7.53, 0.62)	0.24	−6.01 (−9.56, −2.46)	0.007	−4.76 (−7.75, −1.78)	0.02
**Age ≥ 65 years**	0.46 (−2.25, 3.16)		−0.68 (−3.31, 1.96)		−0.22 (−2.70, 2.25)		−0.46 (−2.67, 1.75)	
**Deprivation quartile 1 (low)**	−1.1 (−4.43, 2.24)	0.27	0.58 (−2.53, 3.68)	0.41	−0.52 (−3.96, 2.93)	0.48	0.07 (−2.78,2.92)	0.42
**Deprivation quartile 2**	2.2 (−0.82, 5.22)		−4.9 (−9.49, −0.31)		−2.7 (−7.4, 2.0)		−3.8 (−7.33, −0.27)	
**Deprivation quartile 3**	−1.59 (−5.19, 2)		−1.94 (−5.96, 2.08)		−3.53 (−7.44, 0.37)		−2.72 (−5.84, 0.41)	
**Deprivation quartile 4 (high)**	−3.29 (−9.34, 2.77)		−1.84 (−8.73, 5.06)		−5.13 (−10.23, −0.02)		−3.55 (−8.55, 1.45)	
^**a**^**Body mass index quartile 1 (low)**	−0.21 (−3.06, 2.65)	0.34	−0.25 (−2.79, 2.28)	0.23	−0.46 (−3.1, 2.18)	0.35	−0.34 (−2.46, 1.78)	0.21
**Body mass index quartile 2**	−3.75 (−8.35, 0.85)		0.02 (−5.52, 5.55)		−3.73 (−7.43, −0.03)		−1.76 (−5.93, 2.41)	
**Body mass index quartile 3**	1.01 (−3.69, 5.71)		−3.27 (−8.6, 2.05)		−2.26 (−6.68, 2.16)		−2.64 (−6.5, 1.21)	
**Body mass index quartile 4 (high)**	−0.34 (−4.11, 3.43)		−5.52 (−11.15, 0.1)		−5.86 (−12.0, 0.31)		−5.68 (−10.11, −1.25)	

Data are shown as effect size and 95% confidence interval, and the *p* value is for interaction within each subgroup (age, sex, deprivation score, and body mass index). Adjusted for baseline data

**Table 4:** Post hoc subgroup analysis: analysis of adjusted differences in mean HbA1c at 12 months by self-reported oral hypoglycaemic use shown as effect size and 95% confidence interval

Analysis	INT-DPM vs INT	*p*	INT vs CONTROL	*p*	INT-DPM vs CONTROL	*p*	Combined INT vs CONTROL	*p*
**Oral hypoglycaemic use**	−2.8 (−7.2, 1.49)	0.19	−4.2 (−9.3, 0.77)	0.09	−7.0 (−11.5, −2.5)	0.003	−5.45 (−9.3, −1.6)	0.006
**No oral hypoglycaemic use**	−0.79 (−1.12, 2.69)	0.41	−0.93 (−2.46, 0.59)	0.23	− 0.68 (2.27, 0.92)	0.40	− 0.7 (−2.92, 0.72)	0.33

Adjusted for baseline Hba1c data

Self-reported oral hypoglycaemic (OHG) use at 12 months was CON 44/113 (38.9%), INT 37/93 (39.8%), and INT-DPM 32/88 (36.4%), and 92.5% of OHG use was metformin alone. The adjusted HbA1c effect in intervention arms was statistically significant only in those self-reporting OHG use at 12 months compared to controls (Table 4). This effect was significant in the combined intervention vs controls (Table 4), and only significant in the INT-DPM arm vs controls. The participants who were recorded at 12 months as taking OHG, when compared to those not taking OHG at 12 months, did not differ in mean age at randomisation (65.2 [9.5] years vs 62.6 [9.4] years), or mean BMI (31.8 [5.8] vs 33.1 [6.5] kg/m^2^), but had a higher mean HbA1c at randomisation (58.1 [14.5] mmol/mol vs 50.0 [6.3] mmol/mol; *p* = 0.0001)

### Secondary outcomes

Descriptive data by arm and adjusted differences for secondary outcomes are shown in Tables 5 and 6 and in Additional file 4. There were no significant adjusted differences between groups at 12 and 24 months in biometric or body composition measures, or in health-related quality of life. There was however a significant improvement in fat intake score between INT-DPM and INT group at 12 months (Table 5), and between INT-DPM and CON at 24 months, and an increase in physical activity (resistance activity days per week) at 12 months in both intervention groups compared to controls. There were no significant unadjusted differences between trial arms in DTSQ or DMSES mean scores at 12 or 24 months (data not shown). Questionnaire data on diabetes treatment satisfaction and self-efficacy (DTSQ and DMSES) were not collected at baseline as participants were screen detected and newly diagnosed, and the use of these psychometrically validated questionnaires would be outside licence in newly diagnosed patients. Later DTSQ and DMSES data could not therefore be adjusted for baseline differences. Further descriptive data at 12 and 24 months are shown in Additional file 4: Tables A and B.

**Table 5:** Adjusted differences between groups at 12 months

Analysis	INT-DPM vs INT	***p***	INT vs CONTROL	***p***	INT-DPM vs CONTROL	***p***	Combined intervention vs CONTROL	***p***
**Fasting plasma glucose (mmol/l)**	−0.17 (−0.54, 0.19)	0.35	−0.03 (−0.41, 0.35)	0.87	−0.21 (−0.52, 0.1)	0.19	−0.12 (−0.42, 0.18)	0.44
**Weight (kg)**	−0.29 (−1.63, 1.05)	0.67	0.03 (−1.46, 1.53)	0.96	−0.34 (−1.82, 1.15)	0.66	−0.16 (−1.37, 1.05)	0.79
**Body mass index (kg/m**^**2**^**)**	−0.13 (−0.6, 0.35)	0.60	0.06 (−0.46, 0.58)	0.82	−0.09 (−0.6, 0.43)	0.74	−0.02 (−0.44, 0.41)	0.94
**Body fat (%)**^**a**^	−0.4 (−1.4, 0.6)	0.43	0.93 (1.9, 2.01)	0.09	0.54 (−0.45, 1.54)	0.28	0.74 (−0.12, 1.59)	0.09
**Body fat mass (kg)**^**a**^	−0.57 (−1.82, 0.69)	0.38	0.7 (−0.7, 2.1)	0.32	0.12 (−1.15, 1.38)	0.86	0.41 (−0.69, 1.51)	0.46
**Visceral fat score**^**a**^	0.29 (−0.17, 0.76)	0.21	−0.12 (−0.6, 0.37)	0.63	−0.38(− 0.9, 0.15)	0.16	−0.08 (−0.3, 0.49)	0.70
**Waist (cm)**	0.17 (−1.4,1.74)	0.83	0.33 (−1.29, 1.95)	0.69	0.44 (−1.24, 2.13)	0.61	0.37 (−0.99, 1.73)	0.59
**Physical activity: MET min/week**^**b**^	−958 (−2192, 276)	0.13	671 (−478, 1820)	0.25	−234 (−1478, 1011)	0.71	298 (−680, 1277)	0.55
**Physical activity: resistance days/wk**^**b**^	0.71 (0.29, 1.70)	0.44	4.22 (1.71, 10.44)	0.01	3.32 (1.25,8.80)	0.02	3.81 (1.69, 8.61)	0.01
**Physical activity: min sitting/wk**^**b**^	71.8 (−33, 176)	0.18	−33 (−121, 54)	0.45	38.1 (−56, 133)	0.43	−0.03 (−78, 77)	1.0
**Fat scale score**^**c**^	0.17 (0.05, 0.29)	0.01	−0.26 (−0.07, 0.58)	0.28	0.11 (0, 0.23)	0.06	0.02 (−0.08, 0.11)	0.74
**Fibre scale score**^**c**^	−0.01 (−0.15, 0.13)	0.92	−0.05 (−0.15, 0.05)	0.57	0.01 (−0.12, 0.14)	0.84	0.02 (−0.08, 0.12)	0.71
**General well-being score WBQ-12**^**d**^	−0.54 (−2.35, 1.27)	0.55	−0.12 (−1.21, 1.44)	0.86	−0.61 (−2.16, 0.94)	0.44	−0.27 (−1.52, 0.99)	0.68
**EQ-5D**^**d**^	0.06 (0, 0.12)	0.07	−0.03 (−0.09, 0.02)	0.25	0.03 (−0.03, 0.09)	0.36	0 (−0.05, 0.04)	0.86
**ADDqol**^**d**^	−0.17 (−0.49, 0.16)	0.31	0.1 (−0.18, 0.37)	0.48	−0.06 (−0.34, 0.21)	0.65	0.03 (−0.2, 0.27)	0.79
**HOMA–B (%)**^**e**^	−5.6 (−13.89, 2.68)	0.18	3.38 (−4.8, 11.5)	0.42	−0.43 (−7.53, 6.67)	0.91	1.37 (−5.24, 7.97)	0.68
**HOMA–S (%)**^**e**^	5.28 (−5.87, 16.44)	0.35	−3.6 (−15.3, 8.1)	0.55	−1.01 (−14.65, 12.63)	0.88	−1.32 (−11.71, 9.07)	0.80

Data adjusted for baseline value for each variable and shown as change in mean and 95% CI. ^a^Fat mass (kg and %) by Tanita body composition analyser. ^b^Physical activity scales derived from international physical activity questionnaire IPAQ. ^c^Dietary fat and fibre scores based on self-reported Diet Behaviour Questionnaire (DBQ)—higher fat scale scores indicate lower fat intake. ^d^Well-being score (WBQ-12) questionnaire, health-related quality of life score (EQ-5D) questionnaire, and ADDQoL. ^e^Homeostasis model assessment (HOMA) of baseline insulin sensitivity (S) and beta cell function (B)

**Table 6:** Adjusted differences between groups at 24 months

Analysis	INT-DPM vs INT	***p***	INT vs CONTROL	***p***	INT-DPM vs CONTROL	***p***	Combined intervention vs CONTROL	***p***
**HbA1c (mmol/mol)**	0.11 (−2.46, 2.67)	0.93	−1.29 (−3.77, 1.19)	0.31	−1.18 (−3.8, 1.5)	0.38	−1.24 (−3.35, 0.87)	0.25
**Fasting plasma glucose (mmol/l)**	−0.09 (−0.5, 0.32)	0.66	0.01 (−0.38, 0.41)	0.95	−0.06 (−0.5, 0.4)	0.78	−0.03 (−0.36, 0.31)	0.87
**Weight (kg)**	−0.46 (−2.43, 1.51)	0.39	−1.51 (−4.97, 1.94)	0.39	−1.93 (−5.6, 1.7)	0.30	−1.7 (−4.34, 0.94)	0.2
**Body mass index (kg/m**^**2**^**)**	−0.17 (−0.87, 0.54)	0.46	−0.47 (−1.72, 0.79)	0.46	−0.63 (−1.96, 0.7)	0.35	−0.54 (−1.5, 0.41)	0.26
**Body fat (%)**^**a**^	−0.29 (−1.65, 1.07)	0.67	1.46 (−0.02, 2.94)	0.05	1.14 (−0.32, 2.59)	0.13	1.3 (0.1, 2.49)	0.03
**Body fat mass (kg)**^**a**^	−0.62 (−2.36, 1.12)	0.48	0.98 (−0.95, 2.92)	0.32	0.37 (−1.58, 2.33)	0.71	0.69 (−0.87, 2.26)	0.38
**Visceral fat score**^**a**^	0.16 (−0.5, 0.82)	0.63	−0.27 (−0.99, 0.45)	0.45	−0.36 (−1.15, 0.43)	0.37	−0.06 (−0.66, 054)	0.84
**Waist (cm)**	0.41 (−2.76, 1.93)	0.73	−0.05 (−1.91, 1.81)	0.87	−0.28 (−2.6, 2.1)	0.81	−0.05 (−1.91, 1.81)	0.96
**Physical activity: MET min/week**^**b**^	−698 (−2079, 683)	0.32	482 (−718, 1681)	0.43	−223 (−1433, 987)	0.71	198 (−823, 1220)	0.70
**Physical activity: resistance days/wk**^**b**^	1.03 (0.32, 3.31)	0.96	2.06 (0.71, 5.99)	0.18	1.71 (0.59, 4.94)	0.32	1.90 (0.79, 4.59)	0.15
**Physical activity: min sitting/wk**^**b**^	−115 (−220, −11)	0.03	18.5 (−91.9, 129)	0.74	−94.6 (−200, 11)	0.08	−27.7 (−115, 60)	0.53
**Fat scale score**^**c**^	0.04 (−0.11, 0.18)	0.6	0.11 (−0.02, 0.23)	0.09	0.15 (0.02, 0.27)	0.02	0.12 (0.02, 0.23)	0.02
**Fibre scale score**^**c**^	0.02 (−0.1, 0.15)	0.7	0.07 (−0.08, 0.21)	0.36	0.09 (−0.07, 0.25)	0.26	0.08 (−0.04, 0.19)	0.19
**General well-being score WBQ-12**^**d**^	−0.91 (−2.91, 1.09)	0.37	1.09 (−0.69, 2.88)	0.23	0.48 (−1.41, 2.37)	0.62	0.76 (−0.76, 2.27)	0.33
**EQ-5D**^**d**^	−0.08 (−0.5, 0.33)	0.69	−0.07 (−0.43, 0.29)	0.7	−0.14 (−0.5, 0.22)	0.44	−0.1 (−0.4, 0.19)	0.5
**ADDqol**^**d**^	0 (−0.07, 0.08)	0.97	−0.01 (−0.07, 0.05)	0.72	0 (−0.07, 0.07)	0.99	0 (−0.06, 0.05)	0.89
**HOMA–B (%)**^**e**^	−3.05 (−14.5, 8.5)	0.6	2.8 (−7.04, 12.6)	0.57	1.41 (−7.47, 10.29)	0.75	2.32 (−6.02, 10.66)	0.58
**HOMA–S (%)**^**e**^	−5.3 (−19.6, 8.9)	0.46	0.95 (−15.6, 13.7)	0.90	−5.88 (−20.9, 9.2)	0.44	−3.12 (−15.3, 9.05)	0.61

Data adjusted for baseline value for each variable and shown as change in mean and 95% CI. ^a^Fat mass (kg and %) by Tanita body composition analyser. ^b^Physical activity scales derived from international physical activity questionnaire IPAQ. ^c^Dietary fat and fibre scores based on self-reported Diet Behaviour Questionnaire (DBQ)—higher fat scale scores indicate lower fat intake. ^d^ Well-being score (WBQ-12) questionnaire, health-related quality of life score (EQ-5D) questionnaire, and ADDQoL. ^e^Homeostasis model assessment (HOMA) of baseline insulin sensitivity (S) and beta cell function (B)

### Dose of intervention attained and outcomes

For the INT arm (*n* = 142), 50 (35.2%), 32 (22.5%), and 60 (42.3%) were defined as attaining a low, moderate, or high dose of the intervention, respectively. For the INT-DPM arm (*n* = 141), the equivalent data were 57 (40.4%), 32 (22.7%), and 52 (36.9%). The ‘dose’ effect by intervention arm at 12 and 24 months is shown (Table 7) compared to the low-dose attainment group. There was no significant dose-response’ effect in HbA1c or weight in the INT-DPM group (Table 7). However, the INT group maintained significantly more weight loss to 24 months in those attaining a high dose compared to those attaining a low dose (−5.19 kg; −9.65, −0.74; *p* = 0.02).

**Table 7:** Adjusted changes in weight, fasting plasma glucose, and HbA1c at 12 and 24 months by participants achieving higher ‘dose’ of intervention (moderate, or high) compared to lowest dose participants in each intervention group

	Intervention group				Intervention group-DPM			
	Moderate dose	***p***	High dose	***p***	Moderate dose	***p***	High dose	***p***
**12 months**								
**HbA1c (mmol/mol)** *Adjusted*	**1.69 (−3.4, 6.7)**	**0.50**	**−3.45 (−7.69, 0.78)**	**0.11**	**−3.00 (−6.88, 0.89)**	**0.13**	**−3.17 (−6.72, 0.38)**	**0.08**
**HbA1c (mmol/mol)** *Unadjusted*	**−1.43 (−8.8, 6.0)**	**0.70**	**−8.96 (−15.6, −2.3)**	**0.01**	**0.9 (−3.81, 5.62)**	**0.70**	**1.56 (−2.7, 5.8)**	**0.47**
**Weight (kg)** *Adjusted*	**1.36 (−2.5, 5.3)**	**0.48**	**−3.6 (−6.9, −0.2)**	**0.04**	**−0.73 (−4.67, 3.22)**	**0.71**	**−1.03 (−4.55, 2.49)**	**0.56**
**Weight (kg)** *Unadjusted*	**−1.06 (−4.2, 2.1)**	**0.51**	**−3.28 (−6.1, −0.4)**	**0.02**	**0.72 (−2.2, 3.7)**	**0.63**	**−0.07 (−2.69, 2.54)**	**0.96**
**Fasting plasma glucose (mmol/l)** *Adjusted*	**0.40 (−0.44, 1.23)**	**0.34**	**0.06 (−0.65, 0.76)**	**0.88**	**−0.08 (−0.85, 0.68)**	**0.83**	**−0.06 (−0.76, 0.64)**	**0.87**
**Fasting plasma glucose (mmol/l)** *Unadjusted*	**−0.62 (−1.8,0.56)**	**0.30**	**−1.2 (−2.25, −0.15)**	**0.03**	**0 (−0.8, 0.79)**	**0.99**	**0.15 (−0.57, 0.86)**	**0.68**
**24 months**								
**HbA1c (mmol/mol)** *Adjusted*	**−0.05 (−5.09, 5.00)**	**0.99**	**−3.77 (−8.31, 0.78)**	**0.10**	**4.71 (−1.62, 11.04)**	**0.14**	**2.29 (−3.44, 8.03)**	**0.42**
**HbA1c (mmol/mol)** *Unadjusted*	**−0.21 (−6.9, 6.4)**	**0.95**	**−2.74 (−8.9, 3.5)**	**0.38**	**3.51 (−2.9, 9.9)**	**0.277**	**1.77 (−4.13, 7.67)**	**0.55**
**Weight (kg)** *Adjusted*	**−2.18 (−7.41, 3.06)**	**0.40**	**−5.19 (−9.65, −0.74)**	**0.02**	**−1.45 (−6.07, 3.16)**	**0.52**	**−1.15 (−5.13, 2.83)**	**0.55**
**Weight (kg)** *Unadjusted*	**0.24 (−3.7, 4.2)**	**0.90**	**−2.56 (−6.3, 1.15)**	**0.17**	**−0.75 (−5.23, 3.74)**	**0.74**	**−1.6 (−5.73, 2.54)**	**0.44**
**Fasting plasma glucose (mmol/l)** *Adjusted*	**−0.05 (−0.77, 0.67)**	**0.90**	**−0.42 (−1.09, 0.24)**	**0.20**	**0.09 (−0.77, 0.95)**	**0.84**	**0.10 (−0.68, 0.88)**	**0.79**
**Fasting plasma glucose (mmol/l)** *Unadjusted*	**0.02 (−1.34, 1.37)**	**0.98**	**− 0.17 (−1.43, 1.09)**	**0.79**	**0.16 (−0.76, 1.07)**	**0.73**	**0.44 (−0.45, 1.33)**	**0.32**

Baseline factors adjusted for in dose-response modelling (considered to be potential confounders; Additional File 5; Figure 2) were based on a backward elimination algorithm starting with all baseline measures, retaining those that were significant at the 5% level. Baseline factors adjusted for in dose-response modelling were (a) 12 months INT HbA1c (HbA1c, fat scale score), 12 months INT weight (physical activity, gender, body fat mass, HbA1c, total met minutes per week), and 12 months INT fasting plasma glucose (waist circumference , fasting plasma glucose, body fat mass, fat scale score, and HbA1c); (b) 12 months INT-DPM HbA1c (HbA1c), 12 months INT-DPM weight (baseline BMI), and 12 months INT-DPM fasting plasma glucose (fasting glucose); (c) 24 months INT HbA1c (HbA1c, visceral fat mass, deprivation index, fasting plasma glucose), 24 months INT weight (minutes sitting, weight, family history diabetes), and 24 months INT fasting plasma glucose (age, visceral fat mass, fibre scale score, total MET minutes per week, deprivation index, body fat mass, fasting plasma glucose); and (d) 24 months INT-DPM HbA1c ( total MET minutes per week, HbA1c, EQ5D, deprivation index), 24 months INT-DPM weight (visceral fat mass, ADD QoL, weight, EQ5D, deprivation index, HbA1c, fasting plasma glucose, total MET minutes per week), and 24 months INT-DPM fasting plasma glucose (fasting plasma glucose, body fat mass, physical activity category, BMI, deprivation index). Data is also shown unadjusted for baseline variables

### Health economic analysis

There was an unexplained baseline imbalance in EQ5D scores (Table 1) and return rates for these questionnaires were low at 24 months in each arm: CON (*n* = 64; 43%), INT (*n* = 41; 29%), and INT-DPM (*n* = 31; 22%). The estimates of QALY and incremental cost-effectiveness ratio (ICER) per QALY were therefore not robust and have not been presented.

### Additional non-pre-specified post hoc analyses

We defined diabetes ‘remission’ in a post hoc non-pre-specified analysis at 12 months as HbA1c < 48 mmol/mol combined with a fasting plasma glucose < 7.0 mmol/l. Using these criteria, at 12 months, remission rates were CON 61/119 (51.2%), INT 62/100 (62%), and INT-DPM 56/97 (57.7%).

## Discussion

In this trial, people with newly diagnosed, screen-detected type 2 diabetes received a group-delivered diet and lifestyle intervention, known to be effective in reducing the incidence of type 2 diabetes in high-risk groups [21]. Here, we found the same NDPS lifestyle intervention led to a modest but significant improvement in glycaemic control, particularly for participants receiving additional lifestyle support from trained peer volunteers with type 2 diabetes (DPM). Improvement in mean HbA1c was better in younger participants, and in those self-reporting oral hypoglycaemic use at 12 months. These effects were also most significant in the group receiving additional support from trained volunteers with type 2 diabetes. The glycaemic benefit was less, and not statistically significant, at 24 months.

The value of structured education and lifestyle self-management programmes for people with type 2 diabetes is well established [7–11, 51, 52]. Analysis of clearly defined structured education interventions show a significant reduction in HbA1c, greater efficacy in interventions of more than 12 h in total, and that group support is at least as effective as an individual intervention [51, 52]. In the UK, national recommendations are that diabetes self-management and education should be delivered by trained educators from a range of backgrounds, using an evidence-based curriculum that is quality assured, and initiated at diagnosis [7]. The NDPS lifestyle intervention meets these criteria, has a strong theoretical base in behaviour change and learning theories [21, 22, 24], and can now be added to the small list of lifestyle programmes with trial evidence for efficacy [7], or where a lifestyle prevention intervention has shown glycaemic benefit in type 2 diabetes [53].

The improvement in HbA1c in this trial was most consistent in the intervention arm supported by diabetes prevention mentors (INT-DPM). The statistically significant subgroup effects in the younger age group (< 65 years old), and in those self-reporting OHG use at 12 months, were also confined to the INT-DPM group. There were significant changes in reported lifestyle behaviours in intervention participants. At 12 months, dietary fat scores in the INT-DPM group were significantly different compared to INT arm alone, suggesting INT-DPM participants changed behaviour related to dietary fat reduction compared to the standard intervention arm (INT). The EPIC-Norfolk cohort study, in a population similar to ours, reported a significant direct association between dietary fat intake and HbA1c in type 2 diabetes [54], and this may be a mechanism in the current study. Both intervention groups also showed significant improvement in physical activity (resistance exercise days per week) compared to controls at 12 and 24 months.

The possible longer term clinical benefit of the small intervention effect size we observed in mean HbA1c (between −2 and −3 mmol/mol at 12 months compared to controls, and not sustained to 24 months) is unclear and may not translate to better clinical outcomes. This could only be answered by a long-term follow-up. The HbA1c effect size in this trial is certainly less than the HbA1c effect size seen in meta-analysis of the type 2 diabetes intensive treatment trials that have shown outcome benefit for microvascular end points [55]. Prospective follow-up of people with newly diagnosed type 2 diabetes in a broadly similar population [56], however, has shown that higher HbA1c at 12 months does have predictive value for later complications. Even small early differences in HbA1c are associated with later outcomes, with a 1.1 mmol/mol increase in HbA1c associated with a significant increased later microvascular risk (OR 1.14; 1.05,1.24) [56]. In the trial reported here, the HbA1c reduction effect was greater (−6 to −7 mmol/mol) in younger participants, in those receiving oral hypoglycaemics, and in more obese subgroups, and longer term benefit would be more likely at this level of effect and in these groups [55, 56]. It should also be emphasised that while there are epidemiological direct associations between HbA1c and adverse macrovascular and microvascular complications in type 2 diabetes, therapy-driven HbA1c reductions may have differential effects on macrovascular and microvascular outcomes [55, 57, 58].

There was a substantial loss to follow-up in trial after 12 months (Fig. 1), and the main reason for withdrawal was that participants were unwilling to continue. It should be emphasised that the participants in this trial had been invited to screening primarily for a diabetes prevention research programme, as they were at increased risk of type 2 diabetes, and that the 9% of the eligible population we screened were therefore a self-selecting population interested in their diabetes risk—25% of those with screen-detected type 2 diabetes declined randomisation after diagnosis (Fig. 1). It is possible also that participants in this trial with screen-detected type 2 diabetes were less willing to continue after diagnosis with a lifestyle intervention developed and presented primarily as diabetes prevention lifestyle intervention. These factors should be taken into account in terms of translation to other type 2 diabetes populations, and the generalisability of our findings to non-research clinical settings.

The use of trained lay volunteers (with or without diabetes) to support the clinical management of people with type 2 diabetes is an attractive model in terms of workforce planning and limiting cost [12–18, 59–67]. The level of input from trained lay volunteers can range from participating in simple support groups to acting as a leading provider of care and can be delivered in many clinical settings alongside health care professionals (HCP) [59–67]. In type 2 diabetes, studies describing this model have commonly been in high-income countries in minority populations in low-income settings. These lay volunteer workers have provided education, delivered a standard curriculum, or provided informational and emotional support in addition to the support from HCP [12–18, 59–67]. The use of volunteer peer supporters with type 2 diabetes themselves in delivering a lifestyle intervention with a health care professional (HCP) to people with type 2 diabetes is a less common model that has shown inconsistent outcomes [17, 59–67]. This study is the first to test the added effect of volunteer peer intervention alongside HCP, compared to HCP alone or a control group in newly diagnosed type 2 diabetes.

Efficacy in participants receiving peer support may be more apparent when the peers themselves have high levels of self-efficacy [68, 69]. In this study, DPM diabetes-specific self-efficacy levels were high, and as a group, they were similar in age, medication use, diabetes-specific self-efficacy scores, and well-being (WBQ12) scores as the INT-DPM participants. By definition, the DPM had experienced a longer duration of diabetes than the trial participants had, and 65% of the DPM had had type 2 diabetes for > 4 years [22]. Non-adherence to oral hypoglycaemic use, particularly metformin, in people with type 2 diabetes is often high, with high discontinuation rates [70, 71]. Improved adherence to metformin or other OHG use is associated with better glycaemic control [72] and community health worker or peer support in people with type 2 diabetes may improve medication adherence [65, 73, 74]. However, we did not undertake formal objective assessment of medication adherence other than self-reported measures. DPM training did not allow them to discuss changes in medication directly with participants, and further work should see if active DPM support for medication adherence further improves outcomes in the NDPS intervention. The effect size in mean HbA1c in this trial was significantly greater in those < 65 years old compared to those > 65 years old, and a similar age effect is apparent in meta-analysis of lifestyle and behavioural programmes in type 2 diabetes [52]. A greater glycaemic benefit for peer intervention in type 2 diabetes has been described when the peers are older than the participants and in younger participants [75–77].

The participants randomised in this trial were diagnosed as having type 2 diabetes with the current diagnostic criteria being used between 2011 and 2017 and would be regarded as having reasonable glycaemic control with nearly 90% having a baseline HbA1c < 58 mmol/mol. It is important to make a distinction between people with screen-detected diabetes (as in NDPS) and newly diagnosed type 2 diabetes, as the former have a better prognosis [78], and the potential for reducing HbA1c in newly diagnosed populations is more limited than in populations with higher baseline HbA1c [79]. Lifestyle intervention programmes in type 2 diabetes have usually studied populations with established or newly diagnosed type 2 diabetes and as far as we are aware have not been used in screen-detected type 2 diabetes. The DESMOND type 2 diabetes education programme for patients with established or newly diagnosed T2DM had an estimated cost-effectiveness ICER of £5,387 per QALY, but has not been applied to screen-detected type 2 diabetes [79]. However, we would stress that baseline imbalances in EQ-5D data between arms and low questionnaire return rates at 12 and 24 months precluded a robust estimate of cost-effectiveness in this current study.

The interest in type 2 diabetes ‘remission’ with lifestyle change or treatment has grown in the last few years [80, 81], but was not part of the pre-specified analysis, or an aim of this study. It is also difficult to define remission with different glycaemic diagnostic categories. However, at 12 months, more than half of all participants in each arm recorded a biochemical remission with both an HbA1c < 48 mmol/mol and a fasting plasma glucose < 7.0 mmol/l.

### Limitations

The study population, and the DPM, were largely white and it is unclear if these findings are generalisable to other populations with Type 2 diabetes. The mechanism of the effect of improved glycaemic control also needs further exploration and would require a more detailed collection of medication adherence data and the use of objective dietary and physical activity measures. This is important as the glycaemic effect of the intervention was associated with limited evidence for significant lifestyle changes. This may reflect the complexity of the intervention which had multiple behavioural targets and allowed for considerable individual tailoring of lifestyle changes, and some participants may have achieved better glycaemic control through weight loss and others through engaging in either aerobic or resistance/muscle-strengthening exercise. The heterogeneity of pathways to benefit is well recognised in complex behavioural interventions [82]. The ‘dose response’ data presented in this manuscript should also be interpreted cautiously, as this analysis was not based on participant randomisation or power calculations, and a more detailed process analysis will be undertaken to examine the complex causal pathways to any outcome benefit.

The role of the DPM in changes in dietary behaviour and medication adherence and the mechanisms underlying the age effect are unclear. The improvement in glycaemic control is modest and lasted for 12 months, but it is unknown if this would translate into longer term benefit. The NDPS intervention was also not tested in longer established type 2 diabetes populations and these current data only apply to screen-detected populations, and our health economic data for cost-effectiveness are not robust enough to draw conclusions from. It is also important to take into account the multiplicity of comparisons. For the primary analysis of the primary outcome, for which there were 3 comparisons: (INT vs CON, INT-DPM vs CON, and INT vs INT-SPM), a Bonferroni correction would use a significance level of 1.7%. The main INT-DPM vs CON comparison (*p* = 0.007) is still highly significant at this level. There should also be caution in attaching weight to relatively small isolated significant effects in multiple secondary analyses. One limitation of our pre-specified SAP dose-response modelling that should also be acknowledged is that the decision of what factors to adjust the relationship for was based on a backward elimination approach which is based purely on statistical significance. An alternative approach would have been the manual selection of covariates based on both clinical and statistical significance.

## Conclusions

This trial shows that a diet and lifestyle intervention known to reduce the risk of type 2 diabetes [21] in highest risk glycaemic categories also has glycaemic benefit in people with screen-detected type 2 diabetes when delivered with peer volunteer support, with the most significant effects seen at 12 months in younger participants and in those taking oral hypoglycaemics. These effect sizes were modest, however, and not sustained at 24 months. Our findings suggest that primary care provision of lifestyle interventions to improve diabetes outcomes could be enhanced with the additional support of trained peer volunteers with type 2 diabetes. These findings are relevant to the recent emphasis on community-level interventions and social prescribing through integrated care services or the voluntary sectors [83]. This trial shows that the NDPS intervention has value to clinicians and policy makers, as it is a lifestyle intervention with trial evidence of benefit in both screen-detected type 2 diabetes and in diabetes prevention [21], and can be added to the small list of lifestyle programmes with trial evidence for efficacy in type 2 diabetes [7].

## Supplementary Information

**Additional file 1.** NDPS protocol.

**Additional file 2.** Final statistical analysis plan.

**Additional file 3.** CONSORT checklist.

**Additional file 4.** Additional data Tables A and B.

**Additional file 5: Figure 2**: Directed acyclic graph (DAG).

## Data Availability

The dataset used in this publication is available from the corresponding author on reasonable request.

## Abbreviations

DPF: Diabetes prevention facilitator
DPM: Diabetes prevention mentors: trained volunteers with type 2 diabetes
HCP: Health care professional
ICER: Incremental cost-effectiveness ratio
IFG: Impaired fasting glucose
NDH: Non-diabetic hyperglycaemia
NDPS: Norfolk Diabetes Prevention Study
OHG: Oral hypoglycaemics
QALY: Quality-adjusted life year
SAP: Statistical analysis plan
