# Supplementary material for: Effects of the Norfolk diabetes prevention lifestyle intervention (NDPS) on glycaemic control in screen-detected type 2 diabetes: a randomised controlled trial
Source: BMC Med. 2021 Aug 19;19:183. doi: 10.1186/s12916-021-02053-x (PMC8375190; doi:10.1186/s12916-021-02053-x)
Supplement: Supplementary file 1 — Additional file 1. NDPS protocol. [file 12916_2021_2053_MOESM1_ESM.docx]

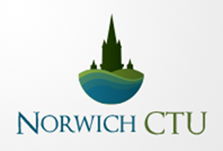


| **Document Title** | **Statistical Analysis plan for NDPS: T2DM (project 4) trial** |
| --- | --- |
| **Author** | **Allan Clark** |
| **Date** | **12/10/2018** |
| **Version** | **1.0** |

# 1. Authorship

| Author | Title | Signature | Date |
| --- | --- | --- | --- |
| Allan Clark | CTU Senior Statistician |  |  |
| Reviewers | Title | Signature | Date |
| Mel Pascale |  |  |  |
| Approver | Title | Signature | Date |
| Mike Sampson |  |  |  |

# 2.0Trial Background

The Norfolk Diabetes Prevention Study, NDPS, is a pragmatic, unblinded, parallel group, three arm randomised controlled trial. The three arms are:

**Arm 1: Control (C)**

Participants in control arm are referred to the local diabetes outpatient clinic as per xxx guidelines. They will also receive a one-off 2 hour dietary & lifestyle education session lasting 2 hours. As this one-off session would not occur in normal practice, the cost of implementing this will be exclude from economic analysis.

**Arm 2: Lifestyle intervention (LI)**

Free access to bespoke diabetes prevention lifestyle programme (NDPS) for a maximum period of 46 months. Six education sessions take place monthly every two weeks for the first three months, followed by “Maintenance” sessions, taking place monthly for up to 15 sessions.

**Arm 3: Lifestyle intervention + supplementary peer support (LIM)**

Free access to NDPS programme as outlined above. Additionally, participants are matched to a DPM (volunteer with an established diagnosis of T2DM) who will provide support throughout intervention period in the form of scheduled telephone calls – frequency xx over xx months.

# 3.0 Populations

The analysis will be based on the following populations:

1. The intention-to-treat population: all participants will be included in the analysis according to the group they were allocated regardless of compliance.
2. The safety population will be all individuals in the control group and all individuals who attended at least one “core” session.

# 4.0 Flow of participants

This will be constructed in accordance with the CONSORT statement, drafted below. Each follow-up time will be presented but only the 6 month follow-up is included below.

Excluded (n=)

-reasons

Diagnosed IFG =

Randomised (n=)

Allocated to LIM

(n=)

- Did not receive

Allocated to LI

(n=)

- Did not receive

Allocated to C

(n=)

- Did not receive

FU available n=

withdrawn n =

finished study n =

FU available n=

withdrawn n =

finished study n =

FU available n=

withdrawn n =

finished study n =

FU: 6 MTH

# 5.0 Research Hypotheses and data

The objectives are split into primary aims and secondary aims.

Primary Aims:

- To determine the effectiveness of Lifestyle intervention plus mentor vs Lifestyle intervention in terms of control of HbA1c.

Secondary aims:

- To determine the effectiveness of Lifestyle intervention vs control in terms of control of HbA1c
- To determine the effectiveness of Lifestyle intervention plus mentor vs control of control of HbA1c
- To determine the effectiveness of Lifestyle intervention or Lifestyle intervention plus mentor vs control in terms of of control of HbA1c
- To compare the secondary outcomes (listed) below between LI vs C
- To compare the secondary outcomes (listed) below between LIM vs C
- To compare the secondary outcomes (listed) below between LIM vs LI

All full list of all baseline characteristics to be summarised is given in section 7.0 and the primary and secondary outcomes in 6.1 and 6.2

# 6 .0 Outcomes

### 6.1 Primary outcome

The primary outcome is the HbA1c at 12 months post randomisation. This differs from the published protocol which states 40 months, but however the decision to change this was taken before database locking or any analysis undertaken so that the sample size and statistical power could be maximised.

### 6.2 Secondary outcomes

The secondary outcome measures are measured at the time-points in the data-collection schedule table. The outcomes are:

1. HbA1c (mmol/mol) at time-points other than the primary time-point
2. HbA1c <=58 mmol/mol
3. HbA1c <=86 mmol/mol
4. Fasting glucose (mmol/L)
5. HDL cholesterol (mmol/L)
6. LDL cholesterol (mmol/L)
7. Insulin (pmol/L)
8. HOMA estimate of insulin sensitivity
9. HOMA estimate of B-cell function
10. physical activity (self-reported short form IPAQ questionnaire)
    1. Total MET minutes per week
    2. Categorical score of physical activity
    3. Minutes of sitting per week
11. resistance activity (self reported study specific questionnaire) measured by question 1c. “How many times per week”.
12. in dietary behaviour (self-reported by DBQ study specific designed questionnaire)
    1. Fat scale
    2. Fibre scale
13. weight
14. BMI
15. waist circumference,
16. body fat %
17. body fat (kg)
18. visceral fat
19. change from baseline in WBQ12 general wellbeing score
20. ADDQoL questionnaire:
    1. Change from baseline in Question 1: “In general, my present quality of life is”
    2. Question 2: “If I did not have diabetes, my quality of life would be”
    3. Weighted impact score
21. change from baseline in EQ-5D utility
22. DMSES score
23. DTSQ questionnaire
    1. Treatment satisfaction
    2. perceived frequency of hyperglycaemia
    3. perceived frequency of hypoglycaemia
24. Weighted composite lifestyle change score based on changes in weight, BMI, waist circumference and exercise levels. This will only be calculated for individuals with a baseline BMI of 30 or greater. The score will range from 0 to 4 with a point given for each of the following: i) weight loss of 7% of baseline BMI or more; ii) moderate or vigorous exercise for 30 min on 5 days of the week or more based on IPAQ questionnaire; iii) positive change in at least 3 items of the Fibre scale; iv) positive change in at least 5 items of the Fat scale.

As multiple measures of HbA1c, Fasting glucose, HDL, LDL, insulin, HOMA can be made at each time point for follow-up time point the first available measurement will be used and for the baseline the last available measurement will be used.

Most of the questionnaire-based outcomes have already been ‘scored’ in the database. However, we will score the following according to the rules:

- Resistance activity. This will be recoded into categories 0 to 7+, with any individual reporting more than seven will be categorized as 7+. Additionally, individuals who have returned the questionnaire and have entries in other questions will be assumed to have 0 for this question.
- DBQ Fat scale will be the average of the observed responses to subscales 1, 2, 3 and 4. This is a maximum of 17 responses. The score will range from 1 to 3, with a high score indicating higher fat intake.
- DBQ Fibre scale will be the average of the observed responses to subscale 5, 6 and 7. This is a maximum of 11 responses. The score will range from 1 to 3 with a high score indicating lower fibre intake.

### 6.3 Schedule of observations

The observations are scheduled as follows

| Variable |  | Time point (months) from randomisation | | | | | | |
| --- | --- | --- | --- | --- | --- | --- | --- | --- |
|  |  |  | | | | | | |
|  |  | +/- 45 days | | | | | | |
|  | Randomisation or screening | 4 | 6 | 12 | 24 | 36 | 40 | 46 |
| HbA1c (mmol/L) | X |  | X | X | X | X | X | X |
| Fasting glucose (mmol/L) | X |  | X | X | X | X | X | X |
| HDL cholesterol (mmol/L) | X |  | X | X | X | X | X | X |
| LDL cholesterol (mmol/L) | X |  | X | X | X | X | X | X |
| HOMA estimate of insulin sensitivity | X |  | x | x | x | x | x | X |
| HOMA estimate of B-cell function | x |  | x | x | x | x | x | x |
| physical activity (self-reported short form IPAQ questionnaire) | X | X | X | X | X | X | X | X |
| resistance activity (self reported study specific questionnaire) | X | X | X | X | X | X | X | X |
| dietary behaviour (self-reported by DBQ study specific designed questionnaire) | X | X | X | X | X | X | X | X |
| weight | X | X | X | X | X | X | X | X |
| BMI | X | X | X | X | X | X | X | X |
| waist circumference, | X | X | X | X | X | X | X | X |
| body fat % | X | X | X | X | X | X | X | X |
| body fat (kg) |  |  |  |  |  |  |  |  |
| visceral fat | x | X | X | X | X | X | X | X |
| change from baseline in WBQ12 | X |  | X | X | X |  | X |  |
| change from baseline in item 1 from ADDQoL | x |  | X | X | X |  | X |  |
| change from baseline in EQ-5D | x |  | X | X | X |  | X |  |
| Change in DMSES | X |  | X | X | X |  | X |  |
| DTSQs | X |  | X | X | X |  | X |  |

# 7.0 Baseline variables to be summarised.

The following baseline variables will be summarised using either the mean and standard deviation if continuous; or the number in each category and the percentage if categorical for each treatment group. No formal comparison based on p-values will be made between groups.

The variables to be summarised are:

| Demographics | Age  Gender  Ethnicity  Smoking status  Recruitment route  Screening site |
| --- | --- |
| Risk factors | First degree relative with T2DM  Personal History of CHD  Gestational diabetes |
| Outcomes | All outcome measures in section 6 available at baseline. |

### 8.0 Treatment allocation

Randomisation of participants is conducted automatically using a dedicated function in the trial data management system. The randomisation mechanism for consists of a pre-prepared random list of codes [for the Intervention and control groups] that are stored in the trial database. The list was built prior to the start of the programme using standard Microsoft tools. Full details are available in the protocol.

### 9.0 Treatment received.

For both the Lifestyle intervention and of Lifestyle intervention plus mentor groups the number of sessions attended and the pattern of attendance will be summarised. For each session by the number and percentage attending each session (education and maintenance) the number will be presented for each group.

For all groups the length of follow-up will be displayed graphically using an inverse Kaplan-Meieir plot. This will be done for days from randomisation until date of withdrawal (or the end of study) and for days from randomisation until date of withdrawal or withdrawal from intervention.

# 10.0 Efficacy Analysis

### 10.1 General principles

The analysis for each outcome measure will use the intention-to-treat (ITT) principle, this implies that all participants will be included in the analysis according to the group they were allocated [1]. All significance tests will be two-sided at the 5% level.

The analysis for all outcomes will be presented in a common format consistent with the CONSORT statement, namely:

1. The number of participants included in the analysis in each study arm;
2. A summary measure of the outcome by study arm. For continuous outcomes this will either be the mean and standard deviation or the median and interquartile range. For binary outcomes the number of "successes" and the percentage of "successes".
3. The estimated treatment effect size with a corresponding 95% confidence interval and p-value. For continuous measures this will be the mean difference. For binary measures it will be the odds ratio and the risk difference.

The analysis will test the following comparisons: C vs LI; C vs LIM; LI vs LIM and C vs (LI or LIM). No adjustment for multiple comparisons will be made as the primary comparison if LI vs LIM and the other comparisons are secondary.

### 10.2 Analysis of primary outcome

For the ITT population, the primary outcome will be analyzed using a t-test. Equal variances will be assessed using Levene’s test. The assumptions of the t-test will be checked using histograms and if evidence of non-normal distribution is found then the sensitivity of the results to this will be assessed using the non-parametric bootstrap. A sensitivity analysis adjusting for age, BMI and IFG levels will also be carried out.

### 10.3 Analysis of secondary outcomes

For secondary outcomes which are continuous the analysis will be as follows. For each the mean difference at 12 months will be compared between the groups using a t-test. This will be called the unadjusted analysis. In addition, an 'adjusted' analysis will also be estimated by including the baseline value of the outcome in a regression model as a fixed effect along with the treatment difference. The assumptions of the modelling will be checked and, if appropriate, a non-parametric bootstrap will be used.

Additionally, in order to take account of the repeated measurements within an individual, a longitudinal analysis will also be undertaken using a linear mixed effects models with individuals included as random effects and will include data from all post-baseline time points using the date of the measurement. Time from baseline will be included in the model as a fixed effect. The decision to use the date of the measurement rather than the allocated time-point has been made to maximize the sample size, however in the case of repeated measurements made at the same allocated time point the earliest will be used. In addition, an 'adjusted' analysis will also be estimated by including the baseline value of the outcome in the model. The comparisons that will be made for an overall mean difference between treatment groups and if there is an interaction between time and the difference between groups.

For secondary outcomes which are ordinal, ordinal logistic regression proportional odds model will be used. If the assumption of proportional odds are not met then a suitable dichotomisation will be applied. The ordinal outcomes are IPAQ category, ADDQoL question 1, DTSQ perceived frequency of hyperglycaemia; DTSQ perceived frequency of hypoglycaemia.

The decision to select a specific time-point, 12 months, for analysis and then a repeated measures analysis is

1. This is the time-point at which the education sessions have finished, is a reasonable level of follow-up, and the minimum amount of follow-up that we offered to the participants.
2. to limit the amount of multiple testing that is undertaken, where by the chance of finding a significant difference increases with the number of tests taken;
3. to limit against over-interpretation of, potential non-significant, results at later time-points due to reduced sample size;
4. The analysis will allow us to see the effect at the end of the education session and then if it is maintained, or changes, over time but in a fashion that controls the rate of false positive findings.

### 10.4 Missing data

A multiple imputation analysis using iteratively chained equations [1] will be used to account for incomplete and missing in outcomes. This will include outcomes that were not observed due to the participant not being in trial for the required period of time, for example individuals will have the 40 month HbA1c outcome imputed even if they were recruited when only 24 month follow-up was possible. The total number of imputations approximately the same as the percentage of cases that are incomplete up to a maximum of 20 imputations. The estimates from imputation will be combined using Rubin's equations. The imputation model used will include all outcome measures and any baseline covariates which are associated with loss-to-follow-up and treatment group.

If any of the variables have a skewed distribution, then transformations will be attempted, however if none are found then predictive mean matching will be used for these variables.

Ordinal outcomes will be included in the imputation model using ordinal logistic regression models.

The imputation model may be adapted during the analysis if

1. Perfect prediction is observed. This occurs when the variance-covariance matrix is singular. If this occurs then ‘augmenting’ will be attempted, but if this does not resolve the issue then variables may be removed from the imputation model.
2. If the imputation procedure does not converge then it will be necessary to remove variables from the imputation model.
3. If the imputation model includes too many variables then instability in the imputation may occur. In this case it may be required to remove variables from the imputation model.

In order to avoid potential issues in model mis-specification, the imputation may not be attempted for outcomes with more than 50% missing data. Multiple imputation will only be used for the intention-to-treat analysis.

### 10.5 Subgroup analyses

Subgroup analysis for the primary outcome in the Intention-to-treat analysis will be carried out for age (<,65 vs >=65 years of age) gender, deprivation index and BMI. Deprivation index and BMI will be split into quartiles. Subgroup analysis will be conducted by testing for an interaction between the subgroup and the randomised group in the primary outcome only. The estimated effect in each subgroup will be estimated and presented separately for each level of the subgroup.

No subgroup analysis was specified in the protocol, however these were added prior to database locked so are still pre-specified.

### 10.6 Within-group analysis

The change in HbA1c will be modelled in each group separately. If no difference is found between the groups then the groups will be combined. Firstly unadjusted regression will be carried out to assess the association between change in HbA1c and each of the factors in the baseline table. Then an adjusted analysis will be undertaken using a “backwards elimination” approach. This approach will include all determinants and sequentially remove the least associated variable until all variables remaining are significantly associated at the 5% level.

In order to assess for a dose-response relationship with between outcome and intervention an analysis will be undertaken in each intervention group separately. The association between dose and change in HbA1c, fasting glucose and weight loss will be estimated using a linear regression model. For the Lifestyle intervention group the dose will be defined as:

1. LOW: less than 30% attendance at sessions;
2. MODERATE: between 30% and 59% attendance at sessions;
3. HIGH: at least 60% attendance at sessions

For the Lifestyle intervention plus mentor the dose will be defined as:

1. LOW: less than 30% attendance at sessions regardless of calls connected OR less than 30% of calls connected regardless of attendance at sessions;
2. MODERATE: between 30% and 59% attendance at sessions and more than 30% of calls connected OR between 30% and 59% of calls connected and more than 30% attendance at sessions;
3. HIGH: at least 60% attendance at sessions AND at least 60% of calls connected.

As this will be a non-randomised comparison adjusted will be undertaken for potential confounding variables.

# 11.0 Safety analyses

The total number of serious adverse events (SAEs) and adverse events (AE) will be tabulated per treatment group based on the safety population. Additionally, the number of individuals with at least one SAE or AE will be tabulated. No formal hypothesis will be tested.

# 12.0 Interim Analysis

No interim analyses will be undertaken.

# 13.0 Data management issues

The database will either be extracted by the data management team or the statistician. The main database will be frozen by data management and the data recorded. Any data queries that are resolved during the analysis phase will require the database to be unfrozen and re-frozen and the data extracted again. The CI will sign the database off as locked and validated to indicate that it is ready for analysis.

# 14.0 Person responsible for completing the analysis:

Either Dr Allan Clark who is a principal statistician with NCTU or a delegated RA under his supervision. They will use Stata version 14 or higher, other packages such as R or SAS may be used if necessary.

### 14.1 Time frame for completing the analysis:

The analysis will take approximately 3 months upon receipt of a clean database, however this may take longer if the number of data queries is high or the assumptions of the methods are incorrect and/or specialist programming is required.

**References**

1. White, Ian R., Patrick Royston and Angela M. Wood (2011) “Multiple imputation using chained equations: Issues and guidance for practice.” *Statistics in Medicine* 30: 377-399

# 15.0 History or revisions post approval

| **Date** | **Change** | **Authorisation** | **Author** |
| --- | --- | --- | --- |
|  |  |  |  |
