# Supplementary material for: Effects of the Norfolk diabetes prevention lifestyle intervention (NDPS) on glycaemic control in screen-detected type 2 diabetes: a randomised controlled trial
Source: BMC Med. 2021 Aug 19;19:183. doi: 10.1186/s12916-021-02053-x (PMC8375190; doi:10.1186/s12916-021-02053-x)
Supplement: Supplementary file 2 — Additional file 2. Final statistical analysis plan. [file 12916_2021_2053_MOESM2_ESM.docx]

Delivering a Realistic Diabetes Prevention Programme in a UK Community:

The Norfolk Diabetes Prevention Study (Norfolk DPS)

Version 14.0

Date: 15th July 2016

Authorised by: Professor Mike Sampson - Chief Investigator

Consultant Diabetologist Diabetes and Endocrinology, Norfolk and Norwich University Hospital NHS Foundation Trust, Norwich, UK & Hon. Chair, School of Medicine, University of East Anglia (UEA), Norwich, UK

Signature (CI or PI): Date:

Study Team

Diabetiologist/ Chief Investigator Professor Mike Sampson

Senior Programme Manager/ Principal Investigator Dr Melanie Pascale

Senior Intervention Research Associate (DPMs) Nikki Murray

Senior Intervention Research Associate (DPFs) Rebecca Usher

Screening Research Facilitator Clare Ferns

Research Assistant Charlie Orton

Fitness Facilitator James Kennedy & Nicky Stroud

Diabetes Prevention Facilitators Rebecca Semmence, Nicky Stroud, Elysia Young, William Hillier

Health Economics Research Associate Lisa Irvine

Research Administrator/PA to Dr Pascale &

PAB coordinator Lucy Webster

Senior Health Care Research Assistants Debbie Thompson & Sarah Wilford

Clerical Officers Elaine Falconbridge, Amoin Dwyer, Donna Kenyon

Volunteers Angela Colman, Nicola Taylor

CO-INVESTIGATORS:

Professor Max Bachmann, Professor of Health Services Research, School of Medicine, Health Policy and Practice, University of East Anglia, Norwich, UK.

Professor Amanda Howe, Professor of Primary Care, School of Medicine, Health Policy and Practice, University of East Anglia, Norwich, UK.

Professor Garry John, Consultant Clinical Biochemistry, Norfolk and Norwich University Hospital NHS Foundation Trust, Norwich, UK.

Dr Tara Wallace, Consultant Diabetologist /Hon Senior Lecturer, Norfolk and Norwich University Hospital NHS Foundation Trust, Norwich and University of East Anglia, Norwich, UK.

Professor Ian Harvey, Executive Dean, Faculty of Health, University of East Anglia, Norwich, UK.

Dr Allan Clark, Statistician, School of Medicine, Health Policy and Practice, University of East Anglia, Norwich, UK.

Dr Ketan Dhatariya, Consultant Diabetologist and Hon Senior Lecturer, Norfolk and Norwich University Hospital NHS Foundation Trust, Norwich, UK.

Dr Garry Barton, Senior Lecturer in Health Economics Modelling, School of Medicine, Health Policy and Practice, University of East Anglia, Norwich, UK.

Dr Martin Hadley-Brown, General Practitioner and Chair, UK Primary Care Diabetes Society (PCDS), School Lane Surgery , Thetford, Norfolk.

Dr Jeremy Turner, Consultant Endocrinologist /Honorary Senior Lecturer, Elsie Bertram Diabetes Centre, Norfolk and Norwich University Hospital NHS Foundation Trust, Norwich, UK.

Dr Jane Smith, Research Fellow in Psychology Applied to Health, University of Exeter Medical School, St Luke’s campus, Heavitree Road, Exeter, EX1 2LU

Mr Krishna Sethia, Medical Director, Norfolk and Norwich University Hospital NHS Foundation Trust, Norwich, UK.

Mr David Rea, Norfolk Diabetes Patient Champion for type 2 diabetes, Norfolk Integrated Diabetes Management (NIDM).

COLLABORATORS

Dr Colin Greaves, NIHR Senior Research Fellow, Peninsula Medical School, University of Chartered Health, Exeter

Dr Alison Woodcock, Senior Lecturer in Psychology, Royal Holloway University of London

Professor Claire Bradley, Professor of Health Psychology, Royal Holloway University of London

Professor Shirley Reynolds, Co-Director Doctoral Programme Clinical Psychology, University of East Anglia, Norwich, UK

Dr Falko Sniehotta, Senior Lecturer in Psychology, School of Psychology, University of Newcastle

Dr Khin Swe Myint, Consultant Physician and Honorary Diabetes, Endocrinology & General Medicine & Principal Investigator for James Paget University Trust and Principal Investigator for James Paget University Hospital

Dr Gerry Rayman, Consultant Physician and Head of Service at the Diabetes and Endocrine Centre, Ipswich Hospital NHS Trust & Principal Investigator for Ipswich Hospital NHS Trust and Principal Investigator for Ipswich Hospital NHS Trust

NDPS PARTICIPANT ADVISORY BOARD

Mr Dave Rea (Chair)

Karen Warrington (Co-ordinator)

Mrs Rosalinde Bailey

Mr Geoffrey Spittle

Mr Tony Finon

Dr Melanie Pascale (PM & PI)

[PROGRAMME OVERVIEW 8](#_Toc303936907)

[SUMMARY OF A TYPICAL PARTICIPANT EXPERIENCE IN THIS PROGRAMME 10](#_Toc303936908)

[LAY SUMMARY 12](#_Toc303936909)

[Screening an ‘at risk’ population to detect people with impaired fasting glucose (IFG). 13](#_Toc303936910)

[Delivering the lifestyle intervention to participants with IFG. 14](#_Toc303936911)

[Delivering the lifestyle intervention in participants with IFG and T2DM using diabetes prevention mentors (DPMs) 15](#_Toc303936912)

[Screen detected T2DM 16](#_Toc303936913)

[Costs and health economic benefit of the programme 16](#_Toc303936914)

[OBJECTIVE 17](#_Toc303936915)

[BACKGROUND 17](#_Toc303936916)

[Setting & Population 20](#_Toc303936917)

[Theoretical Basis and Development of the Intervention 20](#_Toc303936918)

[AIMS 23](#_Toc303936919)

[SELECTION OF CENTRES AND FACILITIES 24](#_Toc303936920)

[Facility requirements for education sessions 24](#_Toc303936921)

[Facility requirements for maintenance sessions 24](#_Toc303936922)

[Primary care collaboration 25](#_Toc303936923)

[STUDY DESIGN 25](#_Toc303936924)

[PROJECT 1 – Screening an at risk population to identify participants with IFG 25](#_Toc303936925)

[Sample selection and size 25](#_Toc303936926)

[Participant Inclusion Criteria for screening 25](#_Toc303936927)

[Participant Exclusion Criteria: 26](#_Toc303936928)

[Recruitment Strategy 26](#_Toc303936929)

[Recruitment through general practice database searches and mailshot: 27](#_Toc303936930)

[Recruitment through a structured media campaign. 27](#_Toc303936931)

[Recruitment through existing retinal screening programme for participants with T2DM 28](#_Toc303936932)

[Recruitment through pharmacists and emerging NHS diabetes and vascular screening programmes. 28](#_Toc303936933)

[Invitation to participate 29](#_Toc303936934)

[Screening Assessment 30](#_Toc303936935)

[Project 1 related Questionnaires 35](#_Toc303936936)

[Questionnaires and interviews for General Practice Staff 36](#_Toc303936937)

[RANDOMISATION 36](#_Toc303936938)

[PROJECT 2: Delivering the Norfolk-DPS lifestyle intervention in participants with IFG. 38](#_Toc303936939)

[Collection of baseline measures for the intervention 38](#_Toc303936940)

[Duration of follow-up and summary of sample size 39](#_Toc303936941)

[Sample Size 39](#_Toc303936942)

[Lifestyle Intervention 39](#_Toc303936943)

[Primary Goals 42](#_Toc303936944)

[Action Plans 42](#_Toc303936945)

[Evaluation of Action Plans 42](#_Toc303936946)

[Intervention Groups Key Characteristics 43](#_Toc303936947)

[Sampling Points and Follow up 45](#_Toc303936948)

[Intervention Group 46](#_Toc303936949)

[Control Group 47](#_Toc303936950)

[Role and training of Diabetes Prevention Facilitators (DPFs) 49](#_Toc303936951)

[PROJECT 3 - Involving people with T2DM as mentors in delivering the Norfolk DPS intervention. 49](#_Toc303936952)

[DPM role 50](#_Toc303936953)

[Recruitment of DPMs 50](#_Toc303936954)

[Training of DPMs 51](#_Toc303936955)

[Telephone calls 51](#_Toc303936956)

[Call structure 52](#_Toc303936957)

[Supervision of DPMs 53](#_Toc303936958)

[Expenses 54](#_Toc303936959)

[Evaluation of the effectiveness of the DPM role 54](#_Toc303936960)

[Qualitative Assessment 55](#_Toc303936961)

[Quantitative Assessment 56](#_Toc303936962)

[Assessment 58](#_Toc303936963)

[PROCESS EVALUATION 58](#_Toc303936964)

[Higher level process analysis 58](#_Toc303936965)

[Intermediate level process analysis 59](#_Toc303936966)

[Finer-grained process analysis 60](#_Toc303936967)

[Collateral effects of intervention on DPMs 60](#_Toc303936968)

[Summary of process evaluation 61](#_Toc303936969)

[STATISTICAL CONSIDERATIONS AND SAMPLE SIZE 64](#_Toc303936970)

[Project 1 64](#_Toc303936971)

[Projects 2 and 3. 65](#_Toc303936972)

[Data analysis and general statistical analysis for Projects 2 and 3. 65](#_Toc303936973)

[PROJECT 5 Economic evaluation of Projects 1- 4. 66](#_Toc303936974)

[Economic evaluation of IFG screening programme. 66](#_Toc303936975)

[Economic evaluation of Norfolk DPS lifestyle intervention. 66](#_Toc303936976)

[Longer term economic modelling of the Norfolk DPS data. 67](#_Toc303936977)

[ENDPOINTS 68](#_Toc303936978)

[Project 2 and 4 68](#_Toc303936979)

[Project 3 69](#_Toc303936980)

[Project 4 69](#_Toc303936981)

[TRANSITION OF FEASIBILITY PROGRAMME PARTICIPANTS TO FULL PROGRAMME 70](#_Toc303936982)

[ATTAINMENT OF PRIMARY END POINTS 70](#_Toc303936983)

[SCREENING LOG 71](#_Toc303936984)

[PROGRAMME CLOSURE 71](#_Toc303936985)

[PARTICIPANT WITHDRAWAL OR EXCLUSION 71](#_Toc303936986)

[PROGRAMME MONITORING 71](#_Toc303936987)

[Access to data 71](#_Toc303936988)

[Confidentiality 72](#_Toc303936989)

[Quality Assurance/Audits/Quality Control of Documents 72](#_Toc303936990)

[Independent data monitoring committee (DMC). 72](#_Toc303936991)

[Strategic trial steering committee. 72](#_Toc303936992)

[Co-Investigator research team meetings. 72](#_Toc303936993)

[Participant Advisory Board (PAB) (Lay members) 72](#_Toc303936994)

[SAFETY REPORTING 73](#_Toc303936995)

[Risks and benefits. 73](#_Toc303936996)

[Potential Benefits 74](#_Toc303936997)

[ETHICAL CONSIDERATIONS 74](#_Toc303936998)

[Equipoise and potential for harm 74](#_Toc303936999)

[Informed consent 75](#_Toc303937000)

[Confidentiality 75](#_Toc303937001)

[Good Clinical Practice and Research Ethics Committee (GCP and REC) 77](#_Toc303937002)

[INDEMNITY 77](#_Toc303937003)

[FINANCE 77](#_Toc303937004)

[PUBLICATION STRATEGY 77](#_Toc303937005)

[APPENDICES 77](#_Toc303937006)

Key Abbreviations

ADDQoL Audit of Diabetes Dependent Quality of Life Questionnaire

BMI Body Mass Index (kg/m^2)^

BP Blood Pressure

CI Chief Investigator

CLRN Comprehensive Local Research Network,

CRTU Clinical Research and Trials Unit

JRO Joint Research Office

CVD Cardiovascular Disease

CI Chief Investigator

DLRN Diabetes Local Research Network

DMSES Diabetes Mellitus Self Efficacy Scale

DMC Data Monitoring Committee

DPF Diabetes Prevention Facilitator

DPM Diabetes Prevention Mentor

DPP Diabetes Prevention Programme (refs19-23)

DTSQ Diabetes Treatment Satisfaction Questionnaire

FFB Fat-and Fiber-related Behaviour Questionnaire

HbA1c Glycated haemoglobin

HOMA-IR Homeostasis Model Assessment of Insulin Resistance

IFG Impaired Fasting Glucose 6.1<IFG <7.0 mmol/L

IGT Impaired Glucose Tolerance (7.8 – 11.1 mmol/l measured two hours after Oral Glucose Tolerance Test)

IPAQ International Physical Activity Questionnaire

NFG Normal Fasting Glucose

NIHR National Institute ~~of~~ for Health Research

NNUHFT Norfolk and Norwich University Hospitals NHS Foundation

Trust, Norwich

Norfolk DPS Norfolk Diabetes Prevention Study

OGTT Oral Glucose Tolerance Test

PAB Participant Advisory Board

Patient Advisor A Norfolk patient helpline staffed by volunteers with diabetes

PCT Primary Care Trust

PCRN Primary Care Research Network

PI Principal Investigator

PIS Participant Information Sheet

PM Programme Manager

PPI Patient Public Involvement

PPiRes Patient and Public Involvement in Research

QALYS Quality Adjusted Life Year

QOF Quality and Outcomes Framework

QRISK2 Cardiovascular Risk Score Estimate

RCT Randomised Controlled Trial

REC / Research Ethics Committee

RGC Research Governance Committee

SOP Standard Operating Procedure

STC Strategic Trials Committee

T2DM Type 2 Diabetes Mellitus

UEA University of East Anglia

UEA-IFG University of East Anglia-Impaired Fasting Glucose

UKPDS UK Prospective Diabetes Study

WBQ-12 Well-Being Questionnaire. 12 item

PROGRAMME OVERVIEW

This protocol describes the research programme ‘Delivering a Realistic Diabetes Prevention Programme in a UK Community: Norfolk Diabetes Prevention Study (Norfolk DPS)’ which is sponsored by the Norfolk and Norwich University Hospitals NHS Foundation Trusts (NNUHFT), Norwich, UK in collaboration with the University of East Anglia (UEA) and funded by a National Institute of Health Research (NIHR) programme grant (E2029M; contract awarded 09/09/2010; award £2.029M). The operational name for the study will be the Norfolk DPS.

This NIHR programme has one primary overarching hypothesis: that the diet and lifestyle intervention we have developed in a preceding NIHR feasibility programme (Essex 1;REC reference: 08/H0301/102; UEA–IFG or feasibility programme) which focuses on changing diet and physical activity, will reduce the risk of transition to type 2 diabetes (T2DM) in groups at highest risk identified through a mass screening programme, compared to a control group receiving standard care alone. The feasibility programme has confirmed our ability to develop this novel diet and lifestyle diabetes prevention programme, to recruit and retain trial participants, to train intervention staff, to develop large bespoke databases, and to create all programme materials. The present programme tests the value of this piloted diabetes prevention intervention in a large randomised control trial. There are very substantial overlaps in methodology, materials developed, and our approach to participants between the present programme and the previously REC approved feasibility programme (Essex 1; 08/H0301/102), but we feel that an entirely new REC submission for this programme is appropriate.

A central and novel part of this programme is the involvement of people with established T2DM who will act as lifestyle mentors to the participants, and work in parallel with health care professionals (HCPs) to deliver it. The present programme tests the value of a refined version of this intervention in diabetes prevention. The Norfolk DPS will be managed centrally in the Clinical Research and Trials Unit (CRTU) at the University of East Anglia / NNUHFT for recruitment within the NHS Norfolk area, with further NHS and non-NHS sites being recruited throughout Norfolk and Suffolk as the programme progresses. Site-Specific Information Form (SSI) will be completed for applications to NHS R&D offices and to NHS Research Ethics Committees for Site-Specific Assessment, where applicable.

We plan to start this 7 year programme on 28.2.2011, and we will screen approximately 10,000 people at risk of type 2 diabetes based on simple criteria developed during the feasibility programme. These people will largely be identified through GP practice database interrogation as in the feasibility programme, but also through developing NHS screening initiatives such as the national vascular screening ‘health checks’ in primary care. This screening programme will extend over 2 years, and volunteers at most risk of T2DM will be defined by a fasting plasma glucose of ≥ 6.1 IFG <7.0 mmol/l (impaired fasting glucose; IFG) and a blood measure of overall glucose control (HbA1c). We estimate that this screening programme and patients followed through from the feasibility programme will deliver a sample size of about 1000 people for randomisation into a control group (n = 170) or two intervention groups (n = 390 each) described in more detail below. The intervention in these groups will be an enhanced version of the intensive diet and lifestyle intervention we have developed in the feasibility programme.

In the feasibility programme (described in detail below) we detected a small but significant signal of reduced mean fasting plasma glucose (p < 0.05) in the intervention group after a mean follow up of 283 days. The present programme will now test the value of this intervention in preventing the transition to T2DM, the primary end point in this programme, in large well powered randomised controlled clinical trials. For people found by chance at screening with previously undiagnosed type 2 diabetes the primary outcome will be HbA1c as a measure of overall glucose control.

One intervention group (n = 390) will receive the lifestyle intervention with the additional support of mentors with type 2 diabetes themselves, as occurred in the feasibility programme, (Diabetes Prevention Mentors; DPMs; and one intervention group will receive the intervention without the DPMs (n = 390), and a control group (n = 170) will receive standard care alone. People found by chance to have previously undiagnosed type 2 diabetes on screening will also be randomised into these 3 groups (estimated 107 in each group, estimated 107 in each intervention group and 47 in control group), with the primary end point being HbA1c as a measure of overall glucose control. Follow up will be for a mean 40 months in all groups, and intervention participants will be asked to have approximately 10 fasting blood tests, 21 visits for education and maintenance sessions, and receive 18 phone calls from a DPM if in the DPM group over the 40 month intervention. Once randomised, all participants in the intervention groups will also be expected to complete questionnaire packs at 0, 4, 6, 12, 24, 36, 40 and 46 months for health economic analyses and psychological assessment of patient reported health and behavioural outcomes. Once randomised, all participants in the control group will also be expected to complete questionnaire packs at 0, 6, 12, 24, 36, 40 and 46 months for health economic analyses and psychological assessment of patient reported health and behavioural outcomes.

Participants in the Feasibility Study (08/H0301/102) have now completed this study. These participants will also be invited to continue through into the full programme in the same groups (control group or intervention with DPMs) and will be re consented on the basis of receiving a NDPS PIS. Those participants in the feasibility programme who do not wish to continue in the full programme will return to GP care and surveillance. This is discussed in detail below.

SUMMARY OF A TYPICAL PARTICIPANT EXPERIENCE IN THIS PROGRAMME

The following describes a typical experience for the participants confirmed with IFG in the most intensive arm of the programme, where the diet and lifestyle intervention is enhanced by the support of Diabetes Prevention Mentors (DPM)

The participants will receive an invitation letter and Participant Information Sheet (PIS) at their home address inviting them to take part in the Norfolk DPS. The PIS is 12 pages long and explains the programme and the letter of invitation will explain the reasons for invitation, which in most cases will be based on their age (≥40 years) and a body mass index ≥30kg/m^2^ taken from their GP records. The participant will be asked to register via either the Norfolk DPS website or the study office by telephone (contact details will be given on the PIS) to discuss the programme. Some basic questions will be asked in order to confirm eligibility for screening and if eligible, the participant will be asked to make a screening appointment. The participants will travel to a nearby facility for screening between 07.45 and 11.15am which is most likely to be the CRTU at UEA. Parking is available and a map and contact details will be provided. The participants will need to fast from 10.00 pm the night before and details about fasting are given with the confirmation of appointment letter. On arrival for screening the participant will be seen by a Healthcare Professional, either a Senior Health Care Assistant (sHCA) or Senior Research Nurse (sRGN). Written informed consent for screening will be obtained from the participant. The healthcare professional will collect screening data, measure height, weight, waist circumference, body fat mass, body fat percentage, visceral fat and take a 15 ml blood sample. The screening should take about 30 minutes. All activities will be oversee my the sRGN.

Patients with raised glucose results will be invited back within 2 weeks for either a repeat fasting plasma glucose (FPG) test, or, if they had a raised fasting result plus an HbA1c result of ≥42mmol/l, an oral glucose tolerance test (OGTT). A diagnosis of T2DM and/or the classification of IFG will be confirmed by the Chief Investigator in all cases, following satisfactory testing. The classification of an impaired fasting glucose (IFG) will be defined as a confirmed fasting plasma glucose (FPG) of ≥ 6.1 - ≤ 6.9 mmol/l, Prediabetes based on the HbA1c analysis will be defined as confirmed normal fasting glucose (NFG) ≤ 5.5 mmol/l plus an HbA1c ≥42 – 47 mmol/mol, and Prediabetes, based on the American Diabetes Association (ADA) will be defined as an FPG ≥5.6 – 6.0 mmol/L plus an HbA1c ≥42 – 47 mmol/mol; this classification will be referred to as IFG_ADA_ from hereon. T2DM diagnosis will be defined as confrimed FPG of ≥7 mmol/l regardless of HbAc1 result and/or a two hour oral glucose tolerence (OGTT) test result of ≥11.1 mmol/l regardless of HbA1c result or confirmed HbA1c results of ≥48 mmol/mol regardless of FPG result. Refer to table below:

| Diagnosis | Confirmed Results | Randomised to Project(s) | Size of project |
| --- | --- | --- | --- |
| T2DM | FPG of ≥7 mmol/l regardless of HbAc1 result and/or a two hour oral glucose tolerance (OGTT) test result of ≥11.1 mmol/l regardless of HbA1c | Main NDPS Trial  Project 4 | N= 300 |
| T2DM | ≥48 mmol/mol & NFG ≤ 5.5 mmol/l | No Trial | N/A |
| Prediabetes | ≥ 6.1 - ≤ 6.9 mmol/l HbA1c <48 mmol/mol | Main NDPS Trial  Projects 2 & 3 | n= 950 in total |
| Prediabetes | FPG ≥ 5.6 – 6.0 mmol/l plus an HbA1c ≥42 – 47 mmol/mol | Main NDPS Trial  Projects 2 & 3 | n= 950 in total |
| Prediabetes | (NFG) ≤ 5.5 mmol/l plus an HbA1c ≥42 – 47 mmol/mol | HbA1c Observational Trial  Projects 2 & 3 | n=200 |
| Normal result | (NFG) ≤ 5.5 mmol/l plus an HbA1c <42 mmol/mol | No Trial | N/A |

Patients who do not fit the above criteria will receive a letter informing them of a normal result. Those with confirmed IFG will be sent a letter inviting them to take part in the next phase of the study (intervention; randomisation into projects 2 &3). Those with confirmed T2DM will be sent a letter inviting them to take part in the next phase of the study (intervention; randomisation into projects 4 n=300). Those with confirmed prediabetes (FPG ≤ 5.5 mmol/l + HbA1c ≥ 42 – 47 mmol/mol) will be sent a letter inviting them to take part in the next phase of the study (intervention into the HbA1c observational trial; randomisation into project 2 n=200) and those patients with confirmed IFG based on the ADA guidelines will be sent a letter inviting them to take part in the next phase of the study (intervention; randomisation into projects 2 &3). If no response from the participant is received after 5 days a member of the study team will contact the participant to ask if they would like to take part in the Intervention.

If the participant wishes to be involved they will attend an appointment to be randomised and for those who are diagnosed as T2DM can also meet the study clinician if requested. After being randomised into the intervention arm by a member of the study team, participants are asked to state a preference for a location to attend the education and maintenance session. They are also asked to provide a time preference for sessions, they are given information about their first session and what will be included in the intervention. They will also be asked to state a preference for days and times of day to receive their motivational phone call from their DPM if randomised into the DPM arm. After the randomisation appointment the participant’s next contact with the team is at their first Education session. Five further education sessions will follow in the participant’s first 12 weeks. After these, participants attend maintenance and physical activity group sessions every 8 weeks to set and review their action plans and goal setting. Between these sessions, participants randomised into the DPM arm will receive motivational phone calls (every 8 weeks) from their assigned DPM.

LAY SUMMARY^^[[1]](#footnote-1)^^

Type 2 diabetes leads to a high concentration of glucose (sugar) in the blood which may over time increase the risk of heart disease and damage to the blood vessels, nerves, eyes and kidneys. There are two types of diabetes ~~-~~ type 1 (which usually develops before the age of 40) and type 2 (adult onset, which is usually diagnosed over the age of 40). Type 2 diabetes is a major and growing health problem in the UK, affecting over 2.4 million people and results in a lower quality of life. This programme is about adult onset type 2 diabetes.

Research studies show that intense efforts to improve diet and physical activity levels halves the risk of developing type 2 diabetes in those at increased risk with ‘pre-diabetes~~’~~. Pre-diabetes is usually defined as a glucose level between 7.8 and 11.1mmol/l after a standard glucose drink or a fasting glucose between 6.1 and 6.9 mmol/l or a normal fasting plasma glucose (NFG; < 6.1 mmol/l), but who fall into the new diagnostic category of prediabetes based on an elevated HbA1c value of ≥42 - 47 mmol/mol inclusive.The first is called impaired glucose tolerance (IGT), the second impaired fasting glucose (IFG) and the latter HbA1c Prediabetes, however all of the above can be referred to as Prediabetes. However, the staffing and level of patient contact required for delivering the interventions in these studies is extraordinarily high and not available in the NHS and in UK primary care. This means that the research studies suggest that the risk of diabetes can be reduced by this type of intense research intervention, but this does not seem to be applicable in the NHS or in general practice. We need a realistic diabetes prevention programme that can be used in the real world of primary care and NHS. We want to test what we think might be an intervention that does this, using group intervention and using lay volunteers to help.

This type of programme has been piloted in our NIHR feasibility study (REC ref:08/H0301/102), known as the UEA-IFG Study, which ended in 2010. This study provided information on participant recruitment, recruitment of lay mentors, GP practice involvement , staff training and IT issues, leading to the creation of a new database and the development of study materials. In the present proposal which leads on from this, we aim to reduce the risk of type 2 diabetes in people at most risk of developing the condition through improved diet and physical activity, and test if the intervention could be delivered cost-effectively in the NHS. The Norfolk DPS contains five related elements.

Screening an ‘at risk’ population to detect people with impaired fasting glucose (IFG).

Before people develop T2DM there is a phase where their blood glucose levels are higher than normal. This phase is known as impaired fasting glucose (IFG) and a blood test, taken after a period of not eating (fasting) can identify whether a person has IFG, which is a blood glucose of ≥ 6.1 IFG <7.0, while diabetes is diagnosed when fasting glucose is above 7.0 mmol/l. In this screening programme we aim to take a blood sample and measure fasting plasma glucose (FPG) and HbA1c (Haemaglobin A1c) as the initial tests to identify people with IFG without adding to the workload on GP practices. We will use the simplest criteria available based on age (≥ 40 years) and body mass index (BMI ~~>~~≥ 30 kg/m^2^) which are universally collected in primary care, to identify ‘at risk’ participants for screening. Over the duration of the study we will screen approximately 10,000 people at risk of developing T2DM. In order to recruit this number of people we will use a process of staggered recruitment from general practices in NHS Norfolk as the main route of recruitment as was done in the feasibility programme, and also media campaigns and self referral, existing retinal screening programmes, pharmacy checks and NHS vascular risk assessment programmes. The retinal screening programme checks the eyes of all people with diabetes in Norfolk every year using digital imaging, and each patient is written to with their results. These people and their families tend to be very diabetes aware, and we plan to add an information sheet about the study to the retinal screening results letter asking people with diabetes to pass this information on to family members and friends. The pharmacy and vascular screening checks programme are national NHS strategies where pharmacy staff and practices are checking peoples’ blood glucose to see if they may be at risk, and we will link this research programme to these NHS services to identify more people with IFG. Anyone identified with IFG through this screening programme will be invited to participate in the trial described below. We expect to find about 1 in 9 people with IFG (using all these strategies) and one in 25 people with undiagnosed T2DM. Anyone found to have IFG has about a 1 in 12 chance of developing T2DM each year, although this figure is variable.

Delivering the lifestyle intervention to participants with IFG.

In this programme (Norfolk DPS) we will measure how successful the intervention is by measuring changes in blood glucose levels and the risk of developing T2DM in people who receive this new intervention (n = 390) compared to controls receiving standard care (n = 170). We do not currently know if this intervention reduces the risk of devloping T2DM, but the Norfolk DPS is large enough and powerful enough to answer this question.

Participants randomised in the intervention group will initially attend 6 x 2 hour education sessions run by one Diabetes Prevention Faciliator (DPF). These 6 sessions will be run as group sessions with randomised participants and will include:

- an introduction to the programme, and to introduce the definitions of IFG and T2DM
- the importance of diet and physical activity
- information session about exercise
- behavioural change
- a review and evaluation of what participants have achieved, and whether their expectations have been met
- how to plan to go forward after the programme ends.
- Explanation of future intervention sessions

Each education session will last 2 hours (12 hours total) and will take place every two weeks at the University of East Anglia (UEA) or at an alternative assessed site.

After completing the education sessions, participants will attend up to 15 less intensive maintenance sessions which include exercise classes run by one DPF and one physiotherapist, over a follow-up period of up to 3.25 years. Each maintenance session will last for 2.5 hours and will take place every 8 weeks (total 37.5hrs) at the UEA or an alternative assessed site. Staff called ‘Diabetes Prevention Facilitators’ (DPFs), will be recruited from multidisciplinary backgrounds to run the lifestyle part of the intervention and will work alongside specialised physical activity falcilitators who will lead exercise sessions. More detailed information on the content of these sessions is given below, but overall we aim to encourage people to lose up to 7% of their body weight through a reduced fat intake and increased physical activity (largely walking).

The control group will receive a single dietary and lifestyle education session which will last for 2 hours and which will take place at the UEA, NNUHFT or an alternative assessed site.

The participants in both intervention groups will have blood samples taken for FPG and HbA1c at: baseline (0) , 6, 12, 24, 36, 40 and exit (46 months).

We will record the views of all participants in the Norfolk DPS using qualitative and quantitative methods on the value of this programme which will be used as part of the evaluation process.

Delivering the lifestyle intervention in participants with IFG and T2DM using diabetes prevention mentors (DPMs)

A unique part of the Norfolk DPS will involve the recruiting and training of members of the public already diagnosed with T2DM to be lifestyle mentors, known as Diabetes Prevention Mentors (DPMs). We will test the additional value of the role of DPMs in further reducing the risk of T2DM in a group with IFG (n= 390) compared to the intervention group without DPM (n = 390) and the control group (n = 170) .

Each participant will receive a 15 minute semi-structured telephone call from the DPM every 4 weeks during the education session phase (first twelve weeks of programme) and then every 8 weeks in between their maintenance and exercise sessions. Based on 390 participants being recruited and each DPM being assigned an average of 8 participants this will need a team of 50 DPMs. We have already successfully recruited, trained and supported 26 DPMs in the feasibility programme, of which 8 will continue through to the Norfolk Diabetes Prevention Study. Furthermore, we have good data on recruitment rates, interview process, and retention in the programme and on DPM group management.

Before the newly recruited DPMs are offered their role they will be:

- formally interviewed by the Senior Intervention Research Associate and the Programme Manager, with possible assistance from a Diabetes Patient Champion (a lay person with diabetes who represents the diabetes community) and a DPM who has continued into the Norfolk DPS from the feasibility study
- subjected to a DBS, refences and occupational health check.

If successful (and they accept the role) the DPMs will be given an NHS honorary contract and receive specific training for their role. Once the programme ends the role of the DPM will finish, but it is hoped that the experience they gain from their role means that they may be able to be used in a similar role within the NHS in the future. We estimate that we will need to recruit and train a further 30 DPMs

Screen detected T2DM

By chance the screening study will also identify people with previously undiagnosed T2DM. These participants (n=240) will be randomised into the three intervention groups described above: a) a control group b) the lifestyle intervention without DPM input c) the lifestyle intervention with DPM input. All of these randomised participants will also receive standard best practice diabetes care through their general practice, and the programme will not interfere with any element of their normal diabetes care. However, if people are found to have diabetes, study clinician will be available via appointment to discuss the diagnosis and implications if the participants wish to do so. However, in all cases the participant will be encouraged to discuss the diagnosis with their GP.

Participants will receive the same frequency of blood testing, education and maintenance sessions as described above. However, in this element of the programme the end point is different – we will ask the question of whether the intervention improves overall blood glucose control (monitored by the results of their HbA1c) which measures average blood glucose levels in the blood over the previous 8 – 10 weeks compared to control treatment. This is the most valuable way overall diabetes control is assessed.

We will use questionnaires and qualitative assessment to measure the impact of the programme on their quality of life compared to standard care only. We will obtain qualitative data by recording the intervention sessions and from interviews, focus groups,conducted with a smaller sample of the intervention group after the intervention sessions.

Costs and health economic benefit of the programme

We will assess the costs of the Norfolk DPS (to NHS and participants) and the cost-effectiveness of the lifestyle intervention with and without DPM input compared to the control group. Using questionnaires we will estimate the cost for each participant screened, assess how taking part may improve health outcomes compared to standard care in the short and long term and ask a random selection of participants for their opinion of the value of the programme using a ‘willingness to pay’ technique. This technique was developed in the feasibility study in an open ended format and this will be used to apply a ‘close ended’ format based on a payment scale questionnaire for the Norfolk DPS.

OBJECTIVE

The primary hypothesis which this programme will test is that the novel diet and lifestyle intervention programme that we have developed will significantly reduce the risk of developing T2DM compared to standard care in participants with IFG.

BACKGROUND

There are now more than 2 million people in the UK with T2DM [^1^](#_ENREF_1) and the associated personal and NHS costs are well described [^2^](#_ENREF_2)^,^ [^3^](#_ENREF_3) with an estimated annual diabetes spend of £9 billion, and 150–200,000 new diagnoses each year [^4^](#_ENREF_4). T2DM is associated with lower quality of life (8-10)[^5-7^](#_ENREF_5) and is a dominant contributor to end-stage renal failure, working-age blindness, vascular medicine activity, and excess bed occupancy in the NHS [^6^](#_ENREF_6)^,^ [^8^](#_ENREF_8)^,^ [^9^](#_ENREF_9). Landmark clinical trials suggest that lifestyle intervention reduces the risk of T2DM in high risk groups. The Diabetes National Strategic Framework stressed that the NHS ‘will develop, implement and monitor strategies to reduce the risk of developing T2DM in the population as a whole' [^2^](#_ENREF_2). This has not been attempted in any structured or commissioned way - we need a realistic structured diet and lifestyle diabetes prevention programme rooted in NHS realities.

People with T2DM may pass through a ‘pre-diabetes~~’~~ stage of glucose intolerance where the fasting plasma glucose is elevated (impaired fasting glucose; IFG) and/or they have an elevated plasma glucose two hours after taking an oral 75g glucose tolerance test (impaired glucose tolerance; IGT ≥ 7.8 IGT <11.1 mmol/l) and/or a prediabetes HbA1c of ≥ 42 – 47 mmol/mol. The definition of IFG developed by the American Diabetes Association, originally ≥ 6.1 IFG <7.0 mmol/l, was lowered to a threshold of 5.6 mmol/l [^10^](#_ENREF_10), the combination of IFG 5.6 – 6.0 mmol/l and a prediabetes HbA1c (42 – 47 47 mmol/mol ) also has a high predictive value for T2DM risk. There is substantial literature on the epidemiological relationships between the risk of vascular disease and the risk of progression to T2DM in these groups, and the metabolic characterisation of each glucose intolerance category .[^10-15^](#_ENREF_10) IFG and IGT are important, as they identify groups at highest risk of progressing to T2DM. Clinical trials of lifestyle interventions in T2DM prevention have generally studied groups with IGT (requiring an oral glucose tolerance test). Meta analysis of 21 trials (up until July 2006) found 17 of adequate quality (8,084 participants). The range of lifestyle interventions were aimed at weight loss and enhanced exercise,and reduced the risk of T2DM to 0.51 (0.44–0.6) with a number needed to benefit of 6.4 (5–8.4). The lifestyle interventions were more effective in those with a higher BMI [^16^](#_ENREF_16). This effect size has been reproduced .[^16^](#_ENREF_16)^,^ [^17^](#_ENREF_17)

The dominant diabetes prevention RCT using a lifestyle intervention is the US Diabetes Prevention Programme (DPP) [^18^](#_ENREF_18), conducted in 27 centres with 3,234 IGT participants, 1079 randomised to a lifestyle intervention for a mean of 2.8 years. The lifestyle intervention had a target weight loss of 7% delivered largely through reduced fat intake and increased walking, underpinned with intense structured contact and interventions [^19^](#_ENREF_19)^,^ [^20^](#_ENREF_20). The cumulative incidence of T2DM was 28.9% (control group) and 14.4% (lifestyle group), a 58% (CI 48-66; p=0.001) reduction. The number needed to benefit was 6.9 (CI 5.4-9.5) for 3 years to prevent one case of T2DM [^19^](#_ENREF_19)^,^ [^20^](#_ENREF_20). The DPP was exceptionally well resourced with one-to-one coaching and intensive telephone contact from healthcare professionals [^19^](#_ENREF_19). The staffing resources required to achieve this result would be an estimated 188 salaried full time lifestyle trainers per 100,000 UK adults, an unlikely level of investment in the current NHS. The translational costs from a research setting to the USA health care system has also been recognised as unaffordable, and the DPP investigators recognised their intervention would need to be delivered in groups of participants [^20^](#_ENREF_20), perhaps by ‘community health workers’, and that this could decrease costs > 70% [^21^](#_ENREF_21). This is an important point, as there is no available validated diet and lifestyle intervention for the prevention of diabetes available in the UK that has been tested in controlled trials, and the research interventions that have been used elswhere to reduce risk can not be applied in a general UK population at risk.

The primary hypothesis and outputs in our programme will be novel, and will offer supporting or validating data for other programme work.

The cultural changes in the NHS emphasise self care as a component of primary and secondary prevention in many chronic conditions. With the increase in the number of diagnosed cases of T2DM and the emerging evidence on expert patient impacts [^24^](#_ENREF_24), a logical extension is to use patient expertise to deliver motivational support to those at risk of the same disease. Wanless et al have suggested that lay involvement in NHS healthcare could be justified in terms of cost containment and skill mix [^25^](#_ENREF_25), and the DPP investigators suggested ‘community health workers’ as a group could deliver their intervention, but it was unclear what background these staff would have [^19^](#_ENREF_19). The role of peer mentors has been investigated with older adults participating in a physical fitness intervention [^26^](#_ENREF_26) and the concept of lay trainers for CVD risk reduction is examined through MRC NPRI funding (with UEA applicants Harvey/Barton as collaborators) but this model has not been examined in disease prevention where the trainers have the disease in question. There are also trial data on the role of such workers in lifestyle interventions working through repeated sessional teaching of self care skills to participants with a range of chronic conditions resulting in enhanced self efficacy and energy and better health related QoL and some limited reduction of costs [^27^](#_ENREF_27). Kennedy et al (2008) have reviewed these roles in the UK and proposed the term ‘Lay Food and Health Worker’ for a role in improving community food and health awareness [^28^](#_ENREF_28). People with T2DM are the obvious choice to train in this role in this programme as they are demographically similar to those with glucose intolerance, are a large enough element in any UK population to develop sufficient mentors, share a common experience of being diabetes aware, and face identical lifestyle challenges. This unique lived and shared experience is absent in generic lay trainer programmes.

Estimates of progression rates from IFG (6.1 ≥ IFG < 7.0 mmol/l) to T2DM in Northern European white populations are available. In the ADDITION study [^29^](#_ENREF_29)annual transition rates were 11.8% in 1821 individuals with IFG (mean age 60yrs; mean BMI 29.1 kg/m2 and similar to our population) [^29^](#_ENREF_29), with a transition rate highest in the first year. In the Inter99 study [^30^](#_ENREF_30), the transition rate from IFG to T2DM was 3.7% for isolated IFG and 10.4% for those with IFG–IGT combined in younger and slimmer participants (age 42.7yrs; BMI 27.7 kg/m2) than in our data and 50% had already had diet and lifestyle interventions. Furthermore, the combination of a prediabetes HbA1c (≥42 – 47 mmol/mol) and an IFG category (IFG_ADA_ 5.6 – 6.9 mmol/l) has very significant additional predictive power for incident T2DM compared to either category alone

The transition rate to T2DM is substantially higher in those with IFG–IGT combined rather than isolated IFG in low risk (11.2%) (18;66) and high risk (10.4%) groups [^30^](#_ENREF_30). About 20% of the IFG participants in this proposal would have combined IFG–IGT and are at higher risk of transition and for power calculations an annual IFG to T2DM incidence rate of 8% is reasonable. Assuming an 8% incidence rate of T2DM per annum (22% cumulative incidence over three years allowing for incident cases) in the control arm and a 4% rate in the Norfolk-DPS arm (12% cumulative incidence in 3 years), then 170 controls and 390 intervention subjects gives 80% power at 5% significance level to detect this difference in proportion progressing (Project 2). Assuming a further reduction in the incidence rate in the Norfolk-DPS with DPM intervention arm to 2% per annum (6% cumulative incidence over 3 years), then 390 additional IFG subjects (Project 3) will provide 80% power at 5% significance level to detect this difference in proportions progressing, compared to the Norfolk-DPS intervention alone (Project 2). Although not a primary end point this intervention will also have 80% power to detect a 12% cumulative incidence compared to controls.

Setting & Population

The population in central Norfolk is 552,658 (25.6% aged 50-74 years; 96.8% white ethnic origin) in 60 main general practices, 36 of these practices have already contributed to the feasibility study. There are also 21 practices and a further population of 145,000 in West Norfolk and 14 practices and a population of 210,000 in East Norfolk. The additional population is 355,000 in West and East Norfolk, and an estimated 221,000 in the untapped 24 central Norfolk practices making a total of 576,000. In addition, extended age criteria (40–45 and 70+ yrs) will increase the population by 20% in the original 36 practices.

Theoretical Basis and Development of the Intervention

The intervention was developed in line with the MRC framework for the development and evaluation of Randomised Controlled Trials for complex health interventions (figure 1). Existing evidence on diabetes prevention programmes and relevant theories guided initial development of the intervention content, structure and techniques. There has been considerable work and feasibility testing of the intervention and evaluation within the pilot study (UEA-IFG), including quantitative and qualitative process evaluations and preliminary cost-effectiveness analyses.

Figure 1 – MRC Framework for Developing, Evaluating and Implementing Complex Interventions (Craig *et al*, 2008)

The intervention maps well onto newly published evidence based guidelines for the prevention of type 2 diabetes [^31^](#_ENREF_31) and the IMAGE toolkit for the prevention of type 2 diabetes in Europe [^32^](#_ENREF_32). This programme and the IMAGE toolkit rely on facilitating behavioural change through participants taking responsibility for changing their behaviour, empowerment of the individual, allowing participants to make choices, and providing a support network.

The principles outlined in the IMAGE toolkit such as FITT (Frequency, Intensity, Time and Type) for physical activity, and Eat Clever (Estimation of the dietary pattern, Aims in short and long term, Tools, Guidance and support, Composition of the diet, Lifestyle for the whole life, Energy, Variety, Evaluation and Risks management) are applied within the intervention materials. An example of how the intervention fits into the process for supporting behaviour change in adults at risk of developing diabetes, and the core philosophy is given in Figure 2.


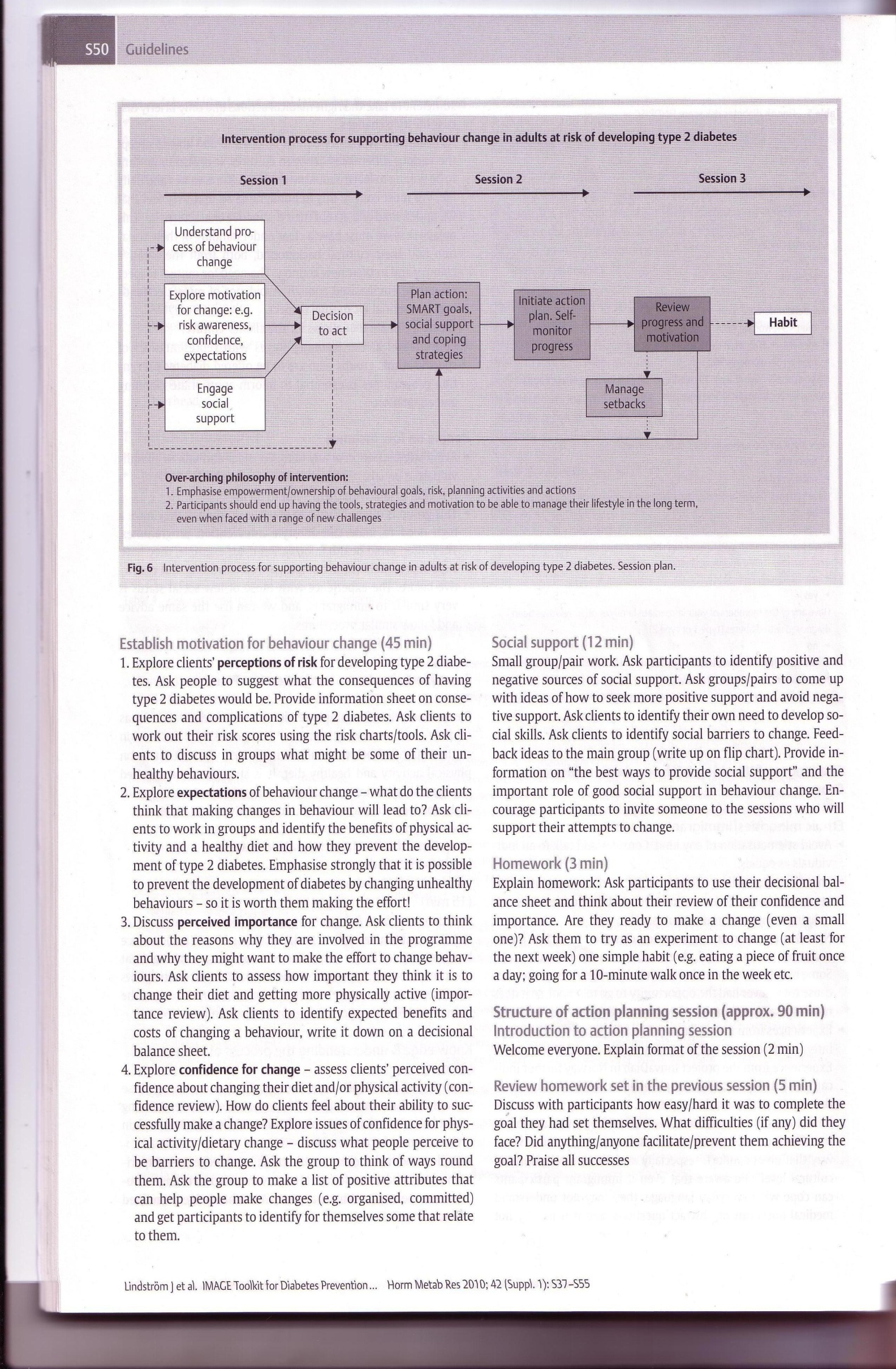


**Maintenance sessions**

**Education sessions**

Figure 2 - How the intervention maps into a process for supporting behaviour change (Lindstrom *et al,* 2010)

The Norfolk-DPS intervention provides many strategies and tools for facilitating initial behaviour change and supporting maintenance of change and addresses the need for individual tailoring. The approach throughout has always been interactive rather than didactic and all the sessions and one to one feedback components allow for interaction and discussion. Reviews suggest the use of psychological theories and techniques in the development of effective interventions [^33^](#_ENREF_33).

Recently published literature on diabetes prevention programmes such as the Greater Green Triangle programme [^34^](#_ENREF_34) and the GOAL programme [^35^](#_ENREF_35), highlight the (HAPA) Health Action Process Approach [^36^](#_ENREF_36) as a basis for these interventions. With this in mind, greater health psychology input will be drawn on to ensure the Norfolk~~-~~DPS Study intervention adopts a similar theoretical underpinning. The 2 stage HAPA model has been found to be successful when focusing on initial behaviour change and also maintenance of behaviour change. [^32^](#_ENREF_32) [^31^](#_ENREF_31) The two stages comprise a motivation stage and a volition (action) stage, with the importance of maintaining behavioural changes and are shown in Figure 3.


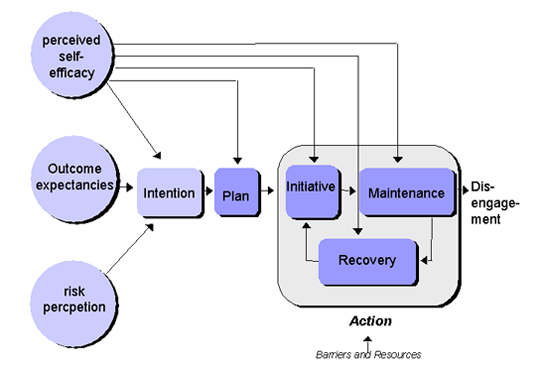


Motivation Stage Volition Stage

Figure 3 - Health Action Process Approach (Schwarzer, 1992)

Many successful physical activity and multi-factor intervention programmes in individuals with IGT and diabetes~~,~~ have consistently used methods that are central to Bandura’s Social Cognitive Theory [^37^](#_ENREF_37), . With the aim of providing a successful intervention, the Norfolk~~-~~DPS intervention also utilises concepts from Social Cognitive Theory such as targeting barriers, increasing self-efficacy and the use of self-regulatory skills. Self-efficacy is targeted in all stages of the intervention, which is again consistent with the HAPA model.

Basing the structure of the education sessions on the HAPA model (Figure 2), the Norfolk DPS education sessions also cover participants’ ideas about risks and progression to T2DM and perceptions and attitudes towards diabetes and pre-diabetes. The sessions further address participants’ outcome expectancies related to the intervention and what participants feel are their main goals. Implementation intentions [^38^](#_ENREF_38) are further utilised to translate decisions and motivation to change behaviours into actions by providing participants with the knowledge to set themselves ‘if-then’ action plans i.e. in line with the planning phase of the HAPA. ‘‘If-then’ plans specify when, where and how a person will perform goal-directed behaviours. ‘If situation y arises then I will perform behaviour X [^39^](#_ENREF_39). Subsequent education sessions combine the use of health education models to provide information relating to nutrition and physical activity; with an emphasis on enhancing individual self-efficiency, goal planning, self-monitoring in the form of diaries and increasing skills to perform health behaviours.

Cognitive Behavioural Therapy principles are acknowledged by recognising the importance of interactions between thoughts, emotions, behaviours and identifying automatic negative thoughts as potentially important barriers to behaviour change [^40^](#_ENREF_40). Final education sessions will focus more heavily on the maintenance of health behaviours. The programme’s intervention also encompasses elements from Empowerment Theory [^41^](#_ENREF_41)^,^ [^42^](#_ENREF_42) with the use of volunteers as DPMs to provide motivational phone calls to the participants throughout the intervention to further assist in the participants’ maintenance of behaviour. This is in line with the modelling technique advocated in Social Cognitive Theory.

AIMS

The overarching aim of this programme is to test the value of a novel diabetes prevention programme in reducing the risk of developing T2DM in a high-risk population, using methods that limit impact on general practice resources.

The following aims are to be tested:

Aim 1: Can we develop a screening programme, based on fasting plasma glucose measurements (IFG/IGT) or the combination of fasting plasma glucose measurements and a with raised HbA1c measrements (IFG_ADA)_ to detect impaired fasting glucose (IFG) in participants at risk of T2DM, without substantial impact on GP practices, using existing NHS infrastructure?

Aim 2: Does our diabetes prevention programme (Norfolk DPS) reduce progression from IFG to T2DM over a 3.25 year intervention compared to controls receiving standard care?

Aim 3: Does using lay mentors (Diabetes Prevention Mentors) with existing T2DM in addition to the Norfolk DPS intervention further reduce the 3.25 year risk of progression from IFG to T2DM compared to the Norfolk DPS intervention alone and to a control group?

Aim 4: Does the Norfolk DPS , with or without DPM, applied to screen detected T2DM, improve glycaemic outcomes and other secondary end points after 3 years compared to standard care?

Aim 5: What are the costs, cost effectiveness and other health economic implications of each aspect of this programme?

Aim 6: Can we detect reliable and informative epigenetic markers that distinguish subjects with normal glucose regulation (NGR) , from those at various degrees of impaired glucose regulation (IGR) including screen detected Type 2 diabetes (T2DM), impaired fasting glucose (IFG) and those with elevated glycosylated haemoglobin levels (HbA1c) in the new WHO pre diabetes range.

SELECTION OF CENTRES AND FACILITIES

For the education sessions and maintenance sessions we will use facilities risk assessed using the standard NNUHFT risk assessment form (Appendix 2) prior to use. The primary locations in first phase will be the CRTU facilities and NNUHFT, and future locations will be largely NHS or healthcare associated facilities

Facility requirements for education sessions

- Availability – day and evening options
- Access to kitchen facility for tea/coffee making (or machine/cafe)
- Wall space for educational material suitable for projection of presentation
- Access to laptop or projector (not essential as programme has their own) or flip chart
- Available chairs (up to 20) and table for educational materials
- Access to toilets
- Disabled access
- An area where participants can be weighed in private

Facility requirements for maintenance sessions

- Availability – day and evening options
- Temperature control, lighting and flooring appropriate for exercise
- Space for up to 15 people to exercise safely
- Access to first aid equipment (and first aider). Programme staff undertake mandatory basic life support training
- Access to changing rooms (not essential). Participants are advised to attend suitably dressed for exercise
- Storage space for some equipment
- Use of some small equipment such as small weights, steps (not essential)
- Access to kitchen facility for tea/coffee making (or machine/cafe)
- Access to drinking water
- Access to toilets
- Disabled access
- An area where participants can be weighed in private

Primary care collaboration

A primary care collaborator whose role will be to liaise between GP surgeries and the research team has been allocated for the duration of the programme, and there has been and will be a close relationship between programme staff and the Primary Care Research Network (PCRN). The feasibility programme launch and close was highlighted in large GP meetings attended by programme staff, and this programme will be launched at a large GP meeting on 20^th^ January 2011, and all NHS Norfolk area practices invited to participate. Potential volunteer surgeries who agree to collaborate on the programme will be allocated a diabetes prevention facilitator (DPF) to conduct database searches. A selection of practice managers, lead GPs or practice nurses will be asked for views in semi structured questionnaire during the screening programme and our approach to practices will be guided by data we have obtained in the feasibility programme.

All participant first contact for screening through GP practices will be undertaken through the practice with no transmission of patient data or materials outside the practice. Our collaboration with primary care will be informed by in-depth quantitative questionnaires and feedback from practices with guidance from primary care co applicant colleagues (Howe; Hadley – Brown). By mid programme, PCT organisations will have ceased and we will ensure good relationships with the successor GP consortia and any new research governance structures that develop.

STUDY DESIGN

Mean follow up in these programmes will be 3.25 years. The overall programme is shown in Figure 5.

PROJECT 1 – Screening an at risk population to identify participants with IFG

Sample selection and size

Over the duration of the Project, we will screen 10,000 people at increased risk of T2DM. To deliver these sample sizes we will use a staggered recruitment of sites to engage general practices. We will randomise 950 people with IFG (including those from the feasibility study) into a randomised controlled trial (RCT) of our intervention: 390 intervention with DPM, 390 Intervention without DPM, and 170 controls receiving standard care.

Participant Inclusion Criteria for screening

Age 40 years or over and one of the below risk factors:

And at least one of the following:

- 1. BMI ≥ 30kg/ m^2^
  2. Parent, sibling or child with T2DM
  3. Personal history of coronary disease
  4. Previous history of gestational diabetes
  5. Known IFG, IGT, IFG_ADA_ and prediabetes

Participant Exclusion Criteria:

1. Not able to provide GP details i.e. not registered with a GP or unwilling for their GP to be contacted
2. Unable to give informed consent due to lack of capacity through severe mental health, learning difficulties or significant cognitive impairment
3. Self-reported conditions which could adversely affect the trial results or patient clinical well being such as:
   1. Terminal illness.
   2. Antipsychotic medication, which may affect glucose tolerance
   3. High dose oral steroids (> 4 weeks or > 7.5 mg)
   4. Active treatment for malignancy
   5. Stage IV renal impairment or ongoing renal dialysis
   6. Pregnant or lactating
   7. Stage IV NYHA cardiac failure
4. Taking part in any research study which involves a dietary or lifestyle change intervention (exceptions are participants in observational research studies and EPIC*) Participation in other research studies will be assessed on an individual basis.
5. Inability to attend or comply with the interventions or follow-up scheduling
6. Living with or related to someone in the programme team
7. GP advice on health grounds that participant should not take part or be contacted

......................................................................................................

*European Prospective Investigation into Cancer (EPIC)

Recruitment Strategy

We will use established recruitment strategies and new recruitment strategies that take advantage of recent NHS developments, to identify participants with IFG

1) General practice database searches and mailshots

2) A structured media campaign and self referral

3) Use existing NHS retinal screening programmes to recruit the friends and family of

patients with T2DM.

4) Pharmacist and NHS Health Checks.

Recruitment through general practice database searches and mailshot:

With a screening capacity of a maximum 10,000 (approximately 100 per week) based on data from our feasibility study), we estimate IFG prevalence to be 5.7% in participants age 40–70 yrs with a BMI ≥30kg/m² therefore, we aim to detect impaired fasting glucose in 570 participants recruited through GP mailshots using these criteria. Potential surgeries will be invited to a Primary Care launch in January 2011 to learn more about the Norfolk DPS. Once a surgery agrees to ‘host’ the study, a member of the research team will be assigned to assist with the conduct of database searches and study related workload. In addition, in one year at this Trust (2009), 36,882 fasting plasma glucose analyses were undertaken at GP and clinician request, of which 12,722 were in the IFG range (6.1 – 7.0mmol/l). Of these, 1,330 were aged > 40 years and did not have diabetes. However , these results were not identified to the requesting practices as abnormal at the time of request. This data from the 2 years previous to study commencement will now be identified to the practice as in the IFG range and accessed through the practice mailshot. In addition, all prospective fasting plasma glucose requests in NNUHFT from primary care and other clinicians will be identified now as abnormal to the GP if they are IFG with a link to the study website and contact details for GPs to access or direct participants towards for participation and screening. Lastly, we will also identify participants with previously undetected T2DM. Administration staff at the practices will undertake database searches (on EMIS, Vision, Systemone, Torex, Synergy, Heathlysoft) and provide a mailing list of potential participants who fit the inclusion criteria. An example of the database search instructions for Vision is included (Appendix 3). Similar manuals will be developed for all other systems. A GP in the practice will check the mailing list for any unsuitable potential participants. A member of the Norfolk DPS team will visit the practice and will send an invitation letter (Document S4) and the Patient Information Sheet (PIS) for the Norfolk DPS (Document S1) to the identified potential participants on the mailing list. Existing data suggests that there are over 120,000 people in Norfolk who have not been contacted and who meet these simple criteria. These people will be the primary target in this recruitment strategy.

We will also place the Norfolk DPS advertisement in the waiting areas of GP surgeries and in the Elsie Bertram Centre at the NNUHFT (Document S2).

Recruitment through a structured media campaign.

The structured media campaign which was used in the feasibility study will be maintained in the Norfolk DPS to raise public awareness of the programme. The media compaign will be based on recruitment criteria described above and involve:

- local and national radio stations and television
- University, Acute Trust, Diabetes UK, Norfolk DPS, Diabtes Research Network and other networks
- advertisements and articles placed in Norfolk and Suffolk newspapers and magazines

This will be a rolling strategy directed at:

- constantly raising public awareness of the programme throughout the recruitment area
- highlighting access to the programme
- distribution of business cards (Document S3) to the family and friends of screened participants

We plan to ask the programme’s Participant Advisory Board to assist in the management of the media strategy within year 1 with support from the PAB representative for the research team

Recruitment through existing retinal screening programme for participants with T2DM

In the fasting hyperglycaemia study [^43^](#_ENREF_43), the most common reason for volunteering for glucose screening was having a relative with diabetes. Norfolk and the surrouinding area has well established community based NHS retinal screening programmes with 90% of the 30,000 people with T2DM in Norfolk receiving retinal screening every 12 months. Everyone in Norfolk with T2DM receives a standard clinical letter summarising their retinal screening findings. In this standard letter we will include an information letter (Document S5) which offers partners, family members and friends who may be intersted, the opportunity to be screened for diabetes. In this way we will contact all families with a family member with diabetes in Norfolk.

Recruitment through pharmacists and emerging NHS diabetes and vascular screening programmes.

The NHS Health Checks [^44^](#_ENREF_44) programme commits health commissioners in primary care trusts (PCT) to developing a primary care screening programme for people at risk of vascular disease from April 2009, and there is substantial commissioning activity for this programme in all PCTs (30). It proposes screening for diabetes in all those aged 40-74 above a BMI threshold ≥ 30kg/^2^ with a fasting plasma glucose or HbA1c. The proposed diabetes pathway is shown in Appendix 4 and the Norfolk DPS recruitment criteria are entirely concordant with this NHS screening programme.

It is unclear what the next steps would be for those found to have impaired fasting glucose, or what the workload implications would be, and concerns have been raised about commissioning, costs and workload impact on practices [^45^](#_ENREF_45). In many areas, pharmacists will be commissioned to deliver this programme. Our proposal fits well with these evolving initiatives which overlaps with our screening programme (2010-2012). At present the plans for NHS Health Checks in Norfolk are in development and will involve a fasting plasma glucose and HbA1c in line with DH advice to all PCTs and may involve pharmacy teams.

These developments are cost neutral (to the Norfolk DPS) and are population wide NHS

developments that will detect large numbers of people with IFG and will be captured through GP database interrogation as above. Throughout the UK, leading pharmacy chains now offer opportunistic patient driven diabetes screening programmes. In collaboration with the Norfolk Local Pharmaceutical Committee we surveyed all 116 pharmacies in Norfolk (July 2009) and found that 11 out of 51 that replied, offer this service, and 30 out of 51 now developing their services (October 2010). Pharmacy screening most commonly involves a random finger prick capillary glucose then, if the random glucose level is above 7.0mmol/l it is followed by a fasting capillary glucose test. Pharmacies currently providing the service in 2009 screened 2,600 per year, with an estimated IFG prevalence of 9.8%. Those with screen detected T2DM are referred to their general practitioners. People with identified IFG (≥ 6.1 - ≤ 6.9 mmol/l) or IFG_ADA_ ( ≥ 5.6 - 6.0 mmol/l plus a HbAc ≥ 42 – 47 mmol/mol) are at present not followed up. The pathway between the pharmacy screening and the Norfolk DPS will involve pharamacists recommending potential glucose intolernate patients to disucss their results with their GP (the current standard pathway). In addition, the participants will be given a study introduction letter from their Pharmacist and a study summary card, with the option of contacting the study team after dsicussion with GP.

These NHS developments offer real economies for the screening strategy and in addition the Norfolk DPS offers an added value to these NHS programmes as at present, people found to have IFG do not have any structured or further intervention.

Invitation to participate

Invitation to participate will be based on piloted Participant Information Sheets (PIS) which have been developed and reviewed by the PPI representatives. Self referrals will be made via the NDPS website or a central study telephone line where programme administrators will check study eligibility with the potential participant via a detailed screening telephone interview prior to booking the screening appointment. Consent will be obtained from each participant prior to recording details on the study database.

In response to participant feedback from the pilot study, we will offer participants the option of screening at fixed satellite primary and secondary care sites across Norfolk and Suffolk "close to home" as well as in central Norwich. This is a well established approach which has been used in other large volume DRN supported studies and will target improving access and recruitment in both urban and rural areas with higher deprivation.

In order to assess eligibility and aid stratification of questionnaires the following information will be collected during the telephone screening interview (refer to Screening Assessment):

- Date of birth
- Gender
- Self reported height and weight (BMI to be calculated: NB. Where participants are eligible to be included for screening based upon self-reported BMI, staff will make them aware that an inaccurate estimate resulting in a BMI < 30kg/m^2^  will result in exclusion from screening if the BMI is the only inclusion criteria)
- risk factors

1. First degree relative with T2DM
2. Personal History of CHD
3. Gestational diabetes
4. Participant identifies themselves as having known IFG or IGT or prediabetes

- GP surgery address

- Participant’s address

- Recruitment route

- Screening site chosen

- Reasons for volunteering and source(s) of information about the programme

Other demographics are taken at screening 1.

Screening Assessment

All procedures will be fully explained by a Senior Health Care Assistant (HCA) or Senior Research Nurse (sRGN) and written informed consent obtained from the participant prior to any procedures being undertaken. All screening activities will be oversee by the sRGN. Following written informed consent the screening assessment will include recording to secure electronic trial database and on the Case Report Form (CRF):

- Date of Birth
- Ethnicity
- Smoking status
- Gender
- BMI
- Waist circumference measurements
- Weight
- Height
- Body fat mass, visceral fat and body fat percentage. This will be measured using a Tanita body fat composition analyser

NDPS Study blood samples – 1^st^ screen

At the first screening appointment two venous blood samples will be taken; one 2ml sample for fasting plasma glucose (FPG) testing and one 4ml sample for HbA1c testing. Blood samples will be sent to the laboratory at the NNUHFT or Ipswich Hospital Trust (dependent on screening location) and will be tested FPG and HbA1C. The laboratories have substantial annual EQA reviews to ensure quality control.

Episwitch Study blood samples

The participant will be asked to consent (consent form S82) for a further 6ml blood sample to be taken at either this first screening appointment or subsequent screening appointments for the Episwitch study. This is entirely voluntary and will not effect their eligiblity to enter the Norfolk Diabetes Prevention Study. In this pilot proposal we will examine the possibility that we can detect reliable and informative epigenetic markers that distinguish subjects with normal glucose regulation (NGR) , from those at various degrees of impaired glucose regulation (IGR) including screen detected Type 2 diabetes (T2DM), impaired fasting glucose (IFG) and those with elevated glycosylated haemoglobin levels (HbA1c) in the new WHO pre diabetes range. We propose to take 6 mls EDTA blood from 300 consecutive participants (approximately 4 weeks) during normal trial recruitment in addition to normal NDPS sample collection. Consented participants will be invited to provide one further blood sample for follow up. This sample will be taken to identify dynamic changes occurring in chromosome conformation signatures during the course of the condition. Dynamic and multi-layered chromosomal loop interactions are powerful regulators of gene expression, determining gene signatures during pathogenic processes. We hypothesise that these chromosomal loop interactions may undergo dynamic changes reflecting remission, stabilisation or relapse. Our aim is to obtain a second sample from consented trial patients to identify stable vs unstable signatures, especially with relevance to their progression or regressio. We propose to take one 6ml EDTA sample at the participant’s next NDPS time point clinic appointment, therefore no additional appointment would be required.

As previously, samples will be stored at –80C in the NHS Clinical Research and Trials Unit for a maximum of 4 weeks and then delivered to Oxford Biodynamics for analysis. The sample will not be allowed to be freeze thaw and will be shipped in an excess of dry ice in an insulated polystyrene box. Samples will be entirely anonymised with no patient identifiers.

Prediabetes Observational Study blood samples

At the first screening appointment for the prediabetes observational study, written informed consent for this observational study will be obtained from the participant prior to any procedures being undertaken. This consent is a separate consent form from the NDPS consent form. All screening activities will be oversee by the sRGN. Following written informed consent the screening assessment will include recording to secure electronic trial database and on the CRF:

• Date of Birth

• Ethnicity

• Smoking status

• Gender

• BMI

• Waist circumference measurements

• Weight

• Height

• Body fat mass, visceral fat and body fat percentage. This will be measured using a Tanita body fat composition analyser

Three venous blood samples will be taken; one 2ml sample for fasting plasma glucose (FPG) testing, one 4ml sample for HbA1c testing plus full blood count (FBC) testing and 4ml clotted sample taken for Ferritin analysis. Blood samples will be sent to the laboratory at the NNUHFT or Ipswich Hospital Trust (dependent on screening location) and will be tested for plasma glucose (FPG) and HbA1C, FBC and Ferritin. The analysis for Haemoglobin via the a full blood count and the Ferritin level will be carried out for these participants of the prediabetes observational study. The HbA1c fraction is abnormally elevated in chronic hyperglycemic diabetic patients and correlates positively with glycemic control. Previous studies suggest that iron deficiency anaemia affects the levels of HbA1c. Serum Ferritin is a marker of iron stores in the body. It has been suggested that iron deficiency must be identified and corrected before any diagnostic decision is made based on HbA1c.

The laboratories have substantial annual EQA reviews to ensure quality control. In 2012 the diagnostic criteria for diabetes were changed, and these changes are now adopted in the UK and internationally. These new criteria are based on measurement of the HBA1c (a blood test that gives an estimate of the average glucose level over the previous 8 – 12 weeks). These new HbA1c criteria (which do not exclude a glucose based diagnosis), indicate a diagnosis of diabetes when the HbA1c is ≥ 48 mmol/l , and also now create a entirely new group of people with ‘pre diabetes’ based on having an HbA1c level of ≥ 42 – 47 mmol/mol (inclusive), who are at increased risk of progressing to diabetes. In the main NDPS study, we have found so far about 350 people of the 5000 screened have this new category of ‘pre diabetes’ but also have had a normal fasting plasma glucose . These people have been told that they have a normal fasting glucose in line with REC approved materials, but this new diagnostic criteria (2012) for diabetes now retrospectively puts these people , unexpectedly, into a prediabetes category. The unusual issue with this group (raised HbA1c but normal fasting glucose) is that it is entirely unknown what their chance of progression to Type 2 diabetes is, or how diet and lifestyle interventions could influence this. This would have very significant research value in answering these questions, and also offer participants in this new category the chance to enter the programme. If participants decline to be screened or randomised we will ask them to make contact with their GP for repeat screening and we will communicate this with the GP.

There are no data that would allow power calculations for this group, and we would like to randomise these subjects (we estimate perhaps 200 would consent) into an observational cohort within NDPS randomised to a control or intervention group, and would like to approach those already screened , and those found to have this abnormality. Once this trial population is complete (n = 200), participants will continue to be offered screening to confirm their dianosis (T2DM) or category (prediabetes). This cohort of participants have a higher prevalence of IFG (10.1%) than participants recruited via the standard NDPS methods (5.7%) and as such in research terms and ethically, it is appropriate to offer these higher risk people access to the main trial if they have screen detected IFG. Therefore the new diagnostic category of prediabetes based on an elevated HbA1c value is incorporated into the main GP search criteria (from 03/2014).

Observational HbA1c research questions

i) What are the progression (or regression) rates of these glycaemic measures (fasting plasma glucose and HbA1c), and transition to T2DM over 3.25 years in this unique population.?

ii) What are the prospective relationships between fasting plasma glucose and HbA1c in this population – is this discordance maintained?

iii) Are this population phenotypically different from the main intervention group (with IFG/IGT)?

iv) What impact does a validated 3 year diet and lifestyle intervention have on the primary end points (diagnostic category, HbA1c and fasting plasma glucose) and secondary end points (weight loss, exercise, anthropometrics etc.) compared to an observation comparator group?

v) What are the downstream health economic and public health implications of these data in UK primary care in relation to new NICE guidance?

vi) Is the HbA1c fraction significantly raised in patients with iron deficiency anaemia whilst correlating positively with glycemic control?

Finally, a small number of people we have screened have had a normal fasting glucose and yet have an elevated HbA1c in the diabetes range (≥ 48 mmol/mol) . We will send letters to these participants offering to repeat these measurements or ask GP practices to do this.

All results will be electronically imported onto a secure electronic trial database.

NDPS Study blood samples – subsequent screening to confirm diagnosis

In addition to the first screening, repeated screenings will also include recording blood pressure measurement and fasting lipids and plasma insulin analysis. Those identified with IFG (6.1 < IFG < 7.0 mmol/l) and an HbA1c ≥42 – 47 mmol/l will be offered a standard oral glucose tolerance test (OGTT) (Appendix 5) to clarify glycaemic status and exclude T2DM [^46^](#_ENREF_46). It is estimated that 56% will require OGTT based on these criteria. This is necessary as a small proportion of people with IFG and an HbA1c ≥42 - 47 mmol/l will have T2DM based on a 2 hour glucose result and it is important to have clarity of diagnosis. If a participant chooses not to have an OGTT we will continue screening using FPG tests until we obtain a confirmed result. Refusal of an OGTT will not exclude the participant from the programme however, if they participate we will offer and encourage them to have an OGTT at a later sampling point if future test results suggest this is necessary

The screening and OGTT protocols are shown in appendix 5 and appendix 6

a) All participants with screen detected IFG will be offered randomisation into the Norfolk DPS and all participants with newly screen detected T2DM will be offered randomisation into the same groups (project 4 n= 300). All participants with screen detected prediabetes (based on normal FPG and raised HbA1c) will be offered randomisation into the NDPS HbA1c observational study until trial is complete (n=200).

b) A randomly selected sample of 200 participants with normal fasting glucose (NFG) will be invited to return for a fasting plasma glucose test at 46 months with repeated weight and BMI measurements.

d) The Epigenetic samples n=300 (based on current data from about 2500 patients) would generate group sizes of approximately

1) 45 subjects with normal fasting glucose, but ‘pre diabetes’ glycated haemoglobin HbA1c (42 – 48 mmol/mol)

2) 15 subjects with impaired fasting glucose (6.1 – 6.9 mmol/l)

3) 15 subjects with T2DM ( based on HbA1c > 48 mmol/mol or fasting plasma glucose > 7 mmol/l)

4) 225 subjects with normal fasting glucose (< 6.1 mmol/l) and HbA1c (< 42 mmol/mol)

We will analyse groups 1 – 3 above and 60 randomly selected age matched controls from the normal glucose regulation group (group 4).

All participants’ blood test results will be sent to their GPs which the participant agrees to when they sign the consent form. If a participant is diagnosed with T2DM, they will receive standard practice care from their GP. Participants will be sent their blood results by letter and may request physiology results. If a participant is found to have iron deficient anaemia their GP will be contacted by a member of the NDPS. Any further testing and patients on going care will be provided by their GP. Results of the Episwitch study will not be sent to their GP.

Project 1 related Questionnaires

A random selection of 600 volunteers entering the screening programme will be asked to complete a study specific pre-screening questionnaire (Document Q15) which will ask for their reasons for recruitment, expectations of screening, and also the validated WBQ-12, EQ-5D, item 1 from the ADDQoL, and study specific questionnaires measuring psychological variables. Particpants will also be asked to complete a study specific Willingness to Pay (WTP), health economic questionnaire.

Once glycaemic status is known we will repeat the procedure using a post-screening trial specific questionnaire (Document Q16); in screen detected NFG, IFG, or T2DM participants (200 in each group). The post-screening questionnaire and a pre-paid envelope in which to return the completed questionnaire will be posted to participants with their screening result letter. It is anticipated that the pre- and post- screening questionnaires will take approximately 10 minutes to complete. We will measure psychological measures, QoL and well-being using study specific questionnaires in screen detected normal fasting glucose (NFG), IFG, or T2DM participants (200 in each group) and baseline dietary and physical actviity data using the FFB, WBQ-12, EQ-5D and item 1 of the ADDQoL questionnaire.

Questionnaires and interviews for General Practice Staff

Practice managers/lead GPs or lead practice nurses will be asked for their views in semi-structured questionnaires during the screening programme.

*Questionnaires*

Study specific pre- and post-screening questionnaires will be sent to all surgeries taking part (92 predicted recruits) and based on findings from feasibility study analysis. Pre-screening questionnaires will be sent to surgeries in the post to be completed prior to the database searches being conducted. The surgery will post the completed questionnaire back to a member of the study team staff. Post screening questionnaires will be handed to the surgery on visit 2 when the mail merge is sent out and surgeries will be handed a SAE to return the questionnaire in within 14 days of the study team’s visit. Questions will include contact information; basic surgery details e.g. number of registered patients, diabetes services provided, and Norfolk DPS participation and workload, practicalities, recruitment and value.

From the 92 predicted recruits, 10-15 practices will be asked to complete detailed questionnaires at 24 months on practice perspectives of the value and practicality of their input to the programme. General Practice selection will be purposive, to ensure a wide demographic cross-section of surgeries, based on pre- and post-screening questionnaires. The questionnaires will be based on the pilot to reflect the key components of the programme and its outcomes, exploring specific issues around feasibility and implications for NHS and patients.

RANDOMISATION

The progress of the participants through the programme to the point of randomisation with points of collection/analysis for the screening tests and measurements is shown at Appendix 10

Participants with IFG or T2DM will be invited by letter to be randomised into the study when they receive their blood results. If they have not contacted the Norfolk DPS team within 5 working days of their results being sent out we will contact them by telephone to offer a randomisation appointment. In addition, participants with T2DM will be offered the opportunity to make an appointment with a study clinician to discuss their diagnosis. We would aim to have appointments confirmed within 28 days of receiving their blood results. Participants who are unable to attend a randomisation appointment within 28 days will be asked to provide another sample for further baseline testing. During the randomisation appointment the interventions will be fully explained to participants verbally by a Diabetes Prevention Facilitator and time taken to answer any queries or concerns. If participants wish to be randomised, written consent will be taken before randomisation to either one of the two intervention groups or the control group.

Randomisation of participants will be conducted using a dedicated function in the trial data management system. The randomisation mechanism consists of a pre-prepared random list of codes (for the Intervention and Control groups) that are stored in a table in the trial database. To reduce the risk of predicting the next allocation while maintaining a reasonable even spread of intervention and control patients, the list will be constructed of blocks of 17 codes (3 Control, 7 intervention and 7 Intervention + DPM) to approximate the proportions of 170, 390 and 390 respectively.

The list will be built prior to the programme start using standard Microsoft tools. Randomisation will be asymmetric to deliver sample sizes described and allow for transition of asymmetric feasibility groups. The last sampling point for randomisation of participants will be 54 months prior to intervention end.

At the randomisation appointment the DPFs will take measurements of: waist, weight, height, BMI (body mass index) and bio-impedance using a Tanita Body Composition Analyser. Bio-impedance is an electronic measurement of the body’s composition (tissue and fluid). The procedure is non-invasive and the measurement is taken using a machine which the participant stands on. BMI is a measurement based on height and weight which calculates how healthy a person’s weight is for their height.

Participants will be informed:

- where to attend (this will be the CRTU at UEA at the start of the programme and will then follow the clinic structure as sites are recruited)
- the length of the appointment (30 minutes)
- that they will be unable to swap groups once randomised

Participants will have to indicate 2 of the following options for availability to receive telephone calls from the DPM if randomised into this group:

- Day of the week
- Time of day (for example: morning, afternoon or evening).

Participants in both the intervention and control groups will be given an accelerometer and asked to wear this for 7 days after randomisation (where accelerometer are avaliable). The accelerometer is a device which records steps taken and minutes of activity and rest per day. The accelerometers are sealed and participants will be unable to see the information recorded. At the end of the 7 day period, participants are asked to return the accelerometer to the study team using a pre-paid envelope. Participants in the intervention groups will be asked to wear an accelerometer at 0, 4, 6, 12, 24, 36, 40 and 46 months.

Participants in the control group will be asked to wear an accelerometer at 0, ~~4~~, 6, 12, 24, 36, 40 and 46 months.

Participants in the two intervention groups will additionally be given a pedometer which shows daily steps taken. The pedometer is intended as a motivational tool to encourage an increase in activity. Pedometers will be given to participants at the first education session and they may retain these throughout the study. The data from pedometers will not be collected for analysis.

Control participants will not be given a pedometer.

PROJECT 2: Delivering the Norfolk-DPS lifestyle intervention in participants with IFG.

Collection of baseline measures for the intervention

Prior to randomisation an appointment with a study staff member (time point 0 months), at the clinic location, all participants eligible for the intervention phase will be required to complete validated questionnaires (EQ-5D, WBQ-12, DBQ, IPAQ 7 day self-administered format, item 1 from the ADDQoL, and study specific questionnaires measuring psychological variables and process analysis measures followed by a health economic Health Service Use questionnaire. After time point 0 months these questionnaires are to be completed at 4 months (Intervention groups and half of the control group), 6 months and annually at participant blood follow-up appointments. Questionnaires will address participant well-being and perceptions of key components of the programme which have or have not yet had an impact. This will provide evidence of reasons for effectiveness /ineffectiveness of the programme and will aid the understanding of the process individuals go through during behaviour change. The IPAQ questionnaire will assess physical activity levels and the DBQ questionnaire dietary component as part of the intervention programme.

Once randomisation group is known, for participants in the intervention group, a pre-participation health questionnaire (approximately 10 minutes) will also need to be completed once at randomisation and returned to the study physical activity facilitator for review prior to the exercise sessions and referred to participant’s GP if required.

Duration of follow-up and summary of sample size

Randomisation of participants will be into a 46 month intervention with last randomisation point at month 60 in the 66 month programme. Participants randomised between months 21 and 24 of screening (121 or 16.5% of new entrants) will have follow up at 36-40 months, but 66.8 % of new entrants (n = 489) will complete 40 months, as will 100% of feasibility study participants (n = 177) giving a mean follow up of 3.25 years. We anticipate that 75% of these people will come into the Norfolk DPS from the feasibility study to form the nested cohort.

Sample Size

The maximum duration of the intervention will be ~~40~~ 46 months with last randomisation point at month 60 in the 66 month programme. NHS premises~~,~~ General Practices and Community locations will be used to deliver the intervention.

The estimated recruitment rate based on feasibility data for screening capacity, IFG prevalance, and consent rate suggests a recruitment rate of 30 - 40 participants per month. Those participants screen detected with IFG or T2DM will be invited to be randomised into the study.

Lifestyle Intervention

The intervention comprises of:

6 x 2 hour education sessions of varying content for the first 12 weeks of the intervention which provide information to:

- encourage decision making about behaviour change
- increase motivation to change
- support lifestyle changes in relation to physical activity and diet
- aid individual goal setting, action planning and self-monitoring

and up to 15 maintenance sessions held 8 weeks apart from month 4 until intervention end which include facilitated discussion and physical activity circuits.

Participants who are identified as having IFG at screening but go on to develop T2DM whilst in the intervention will be offered the opportunity to continue attending the maintenance sessions and to receive DPM calls but will be referred to their GP for their standard diabetes care. These participants will have no further blood tests or clinical measurements taken for the programme but we will ask all participants at randomisation to allow us access to their GP notes data relating to their diabetes management from leaving the programme until programme end. We will also collect health economic and resource use information for the secondary outcomes of this programme, and we will ask participants to complete questionnaires at 6, 12, 24, 36, 40 and 46 months.

Education session plans (Document INT16-21) provide the staff delivering the sessions with a timetable for each session with the rationale for each section. (Document INT 49) provides details of the content of the education and maintenance intervention sessions.

Figure 4 - Flow Diagram of the Norfolk DPS Participant Intervention Pathway

Education Session 1 – Introduction and welcome

Education Session 2 – Fats and healthy eating

Education Session 3 – Getting active

Education Session 6 – Maintenance of goals

Maintenance Session 1 – Energy balance & weight loss

Maintenance Session 2 – Fat busting

Maintenance Session 3 – Managing eating out

Maintenance Session 4 – Health walk

Maintenance Session 5 – Social Support

Maintenance Session 6 – Ways to stay motivated

Maintenance Session 7 – Progression for physical activity

Maintenance Session 8 – Food and mood

Maintenance Session 9 – Health walk

Maintenance Session 10 – Bad days and problem solving

Maintenance Session 11 – Meal planning

Maintenance Sessions 12 – Eat well, Fell well

Randomised into groups

Completion of questionnaires

Receive Mentor phone call

6 month Clinic Appointment & blood tests

12 month Clinic Appointment & blood tests

Maintenance Session 13 – Health walk

Maintenance Session 14 – Stress management

Maintenance Sessions 15 – Reviewing lifestyle changes

24 month clinic appointment & blood tests

**6 Education Sessions over 4 months**

- 2 hour sessions
- groups of 10-15
- 2 weeks apart

Maximum 4 weeks gap

End of education sessions maximum 4 week gap

**Up to 15 Maintenance Sessions**

2.5 hour sessions (groups of 10-15)

**8 weeks apart**

- Body measurements
- Group discussion (DPF)
- Physical activity

40 month clinic appointment & blood tests

Individual targets set using mini action plans before the start of the maintenance sessions.

Action plans used to set new individual goals throughout the maintenance sessions to programme end.

Education Session 5 – Getting stronger

Education Session 4 – Portion control and labels

Participants in the intervention group with mentor will receive telephone calls every 8 weeks from randomisation until the end of the programme

46 month clinic appointment & blood tests

Primary Goals

Participants randomised to the intervention arms will have 4 primary goals to achieve.

1. Where initial BMI ≥30kg/m², 7% weight loss within first 6 months and maintain to study end.

2. Eat more healthily by eating less fat.

3.Build up to 30 mins of moderate physical activity on 5 days per week. This can be achieved by

increasing step count building up to 10,000 steps per day.

4. Perform resistance training 2-3 times per week.

The DPFs and physical activity facilitators will work together with participants to assist them in setting individually tailored goals for them to achieve based on the above study targets. Individual targets will be set by the participant during the maintenance sessions (every 8 weeks) and recorded in the participant’s Action Plans. These action plans will be reviewed, piloted and revised as necessary. The goals will be reinforced by the DPMs during the motivational telephone calls.

Action Plans

Action plans will be used by each participant in the intervention groups (DPM and no DPM) as a measure of setting and achieving physical activity/dietary goals. At the first Education session participants will be taken through the process of completion of their 2 Action Plans (one for physical activity and one for healthy eating) by the DPF facilitating the session. At each maintenance session each participant will also complete their Action Plans (one for physical activity and one for diet). They will be asked to set themselves specific goals in relation to the areas of diet and physical activity which they feel they can meet over the next 8 weeks. At maintenance sessions the first 20 minutes will be spent discussing the groups action plans and if targets were met. If goals were met, reinforcement will be given by the DPF and the group members. If a participant’s goals are not met, the DPF facilitating the session will enquire as to possible barriers that prevented the goals being met and employ relapse prevention techniques. Toward the end of the maintenance session, particpants will be asked to complete new Action Plans for the next 8 weeks and re-set their goals for that time period. This process is reviewed and repeated at every maintenance session. The newly completed Action Plans will be scanned by the study team at the end of each session and handed to the Senior Intervention Research Associate (DPMs) for data entry and analysis. Particpants will retain their original copies at the end of each session as this will aid behaviour change throughout the next 8 weeks before the next maintenance session.

Evaluation of Action Plans

Qualitative and quantitative analysis will be used to evaluate the Action Plans. Analysis will investigate motivation, setting goals (Action Plan), support from significant others (Social support plan), If – Then Plans (Coping Plan), behavioural importance and confidence in carrying out their ch~~o~~osen behaviour.

Qualitative analysis will take place towards the end of the Intervention in the form of focus groups. Participants from the intervention groups with DPM and without DPM will be asked to attend. The focus groups will be run by the Senior Intervention RA (DPM).

Progress Review Sheets

Participants are asked to complete a Progress review sheet at the beginning of each maintenance session. There are two sheets (one for healthy eating and one for physical activity). The sheets contain questions on how many plans did the participant make on their last action plan, did they meet their previous goals set, how satisfied do they feel with their progress to date and did they find completing an Action Plan helping in meeting their goals. The newly completed sheets will be scanned by the study team at the end of each session and handed to the Senior Intervention Research Associate (DPMs) for data entry and analysis. Participants will retain their original copies at the end of each session as this will aid behaviour change throughout the next 8 weeks before the next maintenance session.

Evaluation of Progress review Sheets

Qualitative and quantitative analysis will be used to evaluate the Progress review Sheets. In addition to the mentioned above, analysis will investigate places and events, people, thoughts and feelings on helpful and unhelpful aspects that affect eating and physical activity behaviour.

The area of Action Plans and Progress review Sheets within the Norfolk DPS Intervention will form part of a PhD for the Senior Intervention Research Associate (DPMs)

Intervention Groups Key Characteristics

- Allocation to an education group.
- Assignment to a Diabetes Prevention Facilitator (DPF) as a personal contact throughout the study.
- Attendance at 6 education sessions over 12 weeks.
- Attendance at up to 15 maintenance sessions (a combination of facilitated discussion and physical activity).
- Monitoring of body weight, waist circumference, body fat percentage and visceral fat at maintenance sessions (every 8 weeks). Additionally participants will be encouraged to self monitor their weight at home on a weekly basis.
- Clearly defined weight loss (if appropriate), healthy eating and physical activity goals given verbally, sustained follow up and feedback on study targets for every 8 weeks for up to 3 years.
- The use of Action Plans at every maintenance and education session to help set, review and meet study goals.
- Use of Progress review Sheets at every maintenance session to help review past progress in relation to goal setting.
- An intervention manual has been developed which contains materials and strategies to help with motivation, action planning and maintenance of changes related to dietary and physical activity issues.
- Daily step goals and personalised activity goals.
- To engage in 30 minutes of moderate physcial activity on 5 days of the week.
- Resistance training 2-3 times per week in addition to 30 minutes of physical activity.
- Accelorometers will be used for one week at 0, 4, 6, 12, 24, 36 and 40 month time points to objectively measure detailed physical activity levels.
- Fat and calorie counter booklet and personalised goals for reducing fat intake in the diet.
- Food and activity diaries (as a motivational tool to help meet study targets).
- WBQ-12, EQ-5D, DBQ, IPAQ, item 1 from the ADDQoL and study specific questionnaires including Health Economic and Process Evaluation questionnaires at 0,4, 6, 12, 24, 36 and 40 months to aid monitoring of diet and physical activity and well being.
- Physical Activity Facilitator led group exercise sessions within the maintenance sessions, organised health walks, signposting to local amenities and Government schemes such as the ‘Move More’ campaign to encourage increased physical activity levels.
- For those participating in this arm of the study, allocation to a Diabetes Prevention Mentor (DPM) as an individual peer support for motivation and reinforcement of study and personal goals.
- Regular phone calls at 8 week intervals (DPM intervention arm only).

Participant perspectives on key areas of the intervention sessions, programme positives and negatives will be collated using feedback information from the sessions.

Each intervention group will be asked to complete an evaluation form (Document INT54 and INT55) for one education session and one maintenance session. This is to collate feedback from each participant attending the intervention sessions. Each education and maintenance session will be evaluated by a different group to enable all sessions to be evaluated. The reason evaluation forms will not be completed on a monthly or sessional basis is to avoid over burdening participants with questionnaires.

These will be designed to cover:

- organisational arrangements
- session content
- staff training style
- recommendations

All intervention participants will be asked to complete an end of programme evaluation form (Document INT56) ranking the different content throughout the intervention sessions.

Session feedback will be in the form of a summary (as has already used in the feasibility study) and will be used for qualitative analysis in year 5.

Standard information leaflets from validated external organisations such as Norfolk PCT, Diabetes UK, British Heart Foundation, British Dietetic Association and the British Psychological Society will be given to participants randomised into the intervention and control groups.  These resources are freely available to the public. The participants will maintain the same access to publicly available information leaflets, as if they were not in the study

Sampling Points and Follow up

Participants in both intervention groups will have clinic appointments for body measurements at baseline (0 months) and at 6m, 12m, 24m, 36m, 40m & 46m for blood tests and body measurements and at 0m, 4m, 6m, 12m, 24m, 36m, 40m & 46m for questionnaire measures. Blood samples and blood pressure will not be taken at baseline as the participants will have had these taken at their last screening appointment within 28 days of randomisation. The following tables (#1 Intervention and #2 control) summarise data collection at each follow up visit for both the intervention groups and control group.

Intervention Group

| Outcome Measure | Baseline | 4m | 6m | 12m | 24m | 36m | 40m | 46m |
| --- | --- | --- | --- | --- | --- | --- | --- | --- |
| Fasting plasma glucose | * |  | ✓ | ✓ | ✓ | ✓ | ✓ | ✓ |
| HbA1c | * |  | ✓ | ✓ | ✓ | ✓ | ✓ | ✓ |
| Insulin | * |  | ✓ | ✓ | ✓ | ✓ | ✓ | ✓ |
| Lipid profile | * |  | ✓ | ✓ | ✓ | ✓ | ✓ | ✓ |
| Blood Pressure | * | *✓* | ✓ | ✓ | ✓ | ✓ | ✓ | ✓ |
|  |  |  |  |  |  |  |  |  |
| Height | ✓ | *✓* | ✓ | ✓ | ✓ | ✓ | ✓ | ✓ |
| Weight | ✓ | *✓* | ✓ | ✓ | ✓ | ✓ | ✓ | ✓ |
| BMI | ✓ | *✓* | ✓ | ✓ | ✓ | ✓ | ✓ | ✓ |
| Waist circumference | ✓ | *✓* | ✓ | ✓ | ✓ | ✓ | ✓ | ✓ |
| Body fat mass | ✓ | *✓* | ✓ | ✓ | ✓ | ✓ | ✓ | ✓ |
| Body fat % | ✓ | *✓* | ✓ | ✓ | ✓ | ✓ | ✓ | ✓ |
| Visceral fat | ✓ | *✓* | ✓ | ✓ | ✓ | ✓ | ✓ | ✓ |
| Accelerometer | ✓ | *✓* | ✓ | ✓ | ✓ | ✓ | ✓ | ✓ |
|  |  |  |  |  |  |  |  |  |
| Health Service Use | ✓ |  | ✓ | ✓ | ✓ | ✓ | ✓ | ✓ |
| ADDQoL item 1 | ✓ |  | *✓* | ✓ | ✓ | ✓ | ✓ | ✓ |
| EQ-5D | ✓ |  | *✓* | ✓ | ✓ | ✓ | ✓ | ✓ |
| WBQ-12 | ✓ |  | *✓* | ✓ | ✓ | ✓ | ✓ | ✓ |
|  |  |  |  |  |  |  |  |  |
| DBQ questionnaire | ✓ | *✓* | *✓* | ✓ | ✓ | ✓ | ✓ | ✓ |
| IPAQ | ✓ | *✓* | *✓* | ✓ | ✓ | ✓ | ✓ | ✓ |
| Process Analysis Questionnaire | ✓ |  | *✓* | ✓ | ✓ |  |  |  |
| Q18 (resistance training questionnaire) | ✓ | *✓* | *✓* | ✓ | ✓ | ✓ | ✓ | ✓ |
| Number of study targets met |  |  |  |  |  | ✓ | ✓ | ✓ |
|  |  |  |  |  |  |  |  |  |
| Project 4 only (in addition to above) –  DMSES |  |  | *✓* | *✓* | *✓* | ✓ | ✓ | ✓ |
| ADDQoL |  |  | *✓* | *✓* | *✓* | ✓ | ✓ | ✓ |
| DTSQs |  |  | *✓* |  | *✓* | ✓ | ✓ | ✓ |

*These will be measured at final screening appointment within 28 days of randomisation

Table 1. Sampling and follow up for participants assigned to intervention groups

Control Group

The control group in this programme will be followed annually. Sampling points and data collection is summarised in Table 2 below. The control group will receive standard local best practice in primary care, written NHS information on diet and lifestyle changes [^47^](#_ENREF_47), a Participant Information Sheet and at randomisation will have a discussion with the programme team on the implications of IFG and T2DM. The DPF will deliver the sessions for the control group.

Table 2. Sampling and follow up for participants assigned to the control group

| Outcome Measure | Baseline | 6m | 12m | 24m | 36m | 40m | 46m |
| --- | --- | --- | --- | --- | --- | --- | --- |
| Fasting plasma glucose | * | ✓ | ✓ | ✓ | ✓ | ✓ | ✓ |
| HbA1c | * | ✓ | ✓ | ✓ | ✓ | ✓ | ✓ |
| Insulin | * | ✓ | ✓ | ✓ | ✓ | ✓ | ✓ |
| Lipid profile | * | ✓ | ✓ | ✓ | ✓ | ✓ | ✓ |
| Blood Pressure | * | ✓ | ✓ | ✓ | ✓ | ✓ | ✓ |
|  |  |  |  |  |  |  |  |
| Height | ✓ | ✓ | ✓ | ✓ | ✓ | ✓ | ✓ |
| Weight | ✓ | ✓ | ✓ | ✓ | ✓ | ✓ | ✓ |
| BMI | ✓ | ✓ | ✓ | ✓ | ✓ | ✓ | ✓ |
| Waist circumference | ✓ | ✓ | ✓ | ✓ | ✓ | ✓ | ✓ |
| Body fat mass | ✓ | ✓ | ✓ | ✓ | ✓ | ✓ | ✓ |
| Body fat % | ✓ | ✓ | ✓ | ✓ | ✓ | ✓ | ✓ |
| Visceral fat | ✓ | ✓ | ✓ | ✓ | ✓ | ✓ | ✓ |
| Acceler  ometer | ✓ | ✓ | ✓ | ✓ | ✓ | ✓ | ✓ |
|  |  |  |  |  |  |  |  |
| Health Service Use | ✓ | ✓ | ✓ | ✓ | ✓ | ✓ | ✓ |
| ADDQoL item 1 | ✓ | ✓ | ✓ | ✓ | ✓ | ✓ | ✓ |
| EQ-5D | ✓ | ✓ | ✓ | ✓ | ✓ | ✓ | ✓ |
| WBQ-12 | ✓ | ✓ | ✓ | ✓ | ✓ | ✓ | ✓ |
|  |  |  |  |  |  |  |  |
| ~~FFB~~ DBQ questionnaire | ✓ | ✓ | ✓ | ✓ | ✓ | ✓ | ✓ |
| IPAQ | ✓ | ✓ | ✓ | ✓ | ✓ | ✓ | ✓ |
| Q18 (resistance training questionnaire) | ✓ | ✓ | ✓ | ✓ | ✓ |  |  |
| Process Analysis Questionnaire *(HALF CONTROL GROUP ONLY)* | ✓ |  | ✓ | ✓ |  | ✓ | ✓ |
|  |  |  |  |  |  | ✓ | ✓ |
| Project 4 only (in addition to above) –  DMSES |  | *✓* | *✓* | *✓* | ✓ |  |  |
| ADDQoL |  | *✓* | *✓* | *✓* | ✓ | ✓ | ✓ |
| DSTQs |  | *✓* |  | *✓* | ✓ | ✓ | ✓ |
|  |  |  |  |  |  | ✓ | ✓ |

*These will be measured at final screening appointment within 28 days of randomisation

Participants in all groups will be given an accelerometer to measure step count and physical activity for one week at each time point in order to investigate the efficacy of the intervention. Accelerometers provide an objective measure of physical activity. Participants will be asked to return their accelerometer at the end of the week and will be supplied with a stamped addressed envelope in which to return them to the Norfolk DPS office.

Role and training of Diabetes Prevention Facilitators (DPFs)

DPFs will be recruited from multidisciplinary backgrounds and assigned to groups of intervention participants for the duration of the study. The role of the DPF is to:

- support and encourage the participants throughout the programme.
- deliver the education sessions and the single session for the control group.
- provide feedback to participants.
- work alongside physical activity facilitators who will deliver the physical activity sessions during the maintenance sessions and will be managed by the Senior Intervention DPF RA with access to, the Senior Intervention DPM RA and health psychologists as and when necessary.
- Undertake adminstrative tasks e.g. related to recruitment, distribution/collection of questionnaires/action plans.

PROJECT 3 - Involving people with T2DM as mentors in delivering the Norfolk DPS intervention.

In this 40 month project we will test the hypothesis that the involvement of people with T2DM as lay Diabetes Prevention Mentors (DPM) in the Norfolk DPS will further significantly reduce the incidence of T2DM in the IFG population, compared to participants receiving the intervention alone (without DPM). Participants joining the Norfolk DPS from the feasibility study have already received this intervention and will form a nested cohort within the Norfolk DPS intervention group with DPM.

We will asymmetrically randomise screen detected IFG participants (Project 1) to the intervention group with DPM (n=390), the intervention group alone (n=390) or the control group (n=170). We will select, interview and train up to a maximum of 40 new DPMs to join the Norfolk DPS team if required, in addition to those who choose to continue through from the feasibility study.

Inclusion Criteria

- Diagnosed with T2DM for at least 2 years
- Be over the age of 18 years

Exclusion Criteria

1. Not able to provide GP details i.e. not registered with a GP or unwilling for their GP to be contacted
2. Unable to give informed consent due to lack of capacity through severe mental health, learning difficulties or significant cognitive impairment
3. Taking part in any research study which involves a dietary or lifestyle change intervention (exceptions are participants in observational research studies and EPIC*). DPMs in other research studies will be assessed on an individual basis.
4. Inability to attend or comply with the follow-up scheduling
5. Living with or related to someone in the programme team
6. GP advice on health grounds that participant should not take part or be contacted
7. Sever hearing loss

DPM role

The DPMs will:

- act as mentors to support participants in the Norfolk DPS through direct phone contact to encourage weight loss, healthy eating and physical activity targets
- aid training of newly recruited DPMs if required
- represent the Norfolk DPS in the role of a lay member

Recruitment of DPMs

Recruitment will be achieved via media advertisement (Document D19), poster advertisements at the Elsie Bertram Diabetes Centre at the NNUH (Document D17) and through mail shot from pre-selected GP surgeries. Prior to interviews, if eligible, potential DPMs will be required to complete a pre-recruitment questionnaire (Document Q3). DPM interviews will be conducted by the Senior Intervention RA leading this area, a NNUHFT Diabetes Patient Champion who is a lay member representing the diabetic community and a DPM who has continued through from the feasibility study.

Based on 390 participants being recruited and each DPM being assigned 8 participants this will equal a need for a team of 50 DPMs. We will endeavour to recruit, interview and train more lay members throughout the duration of this programme. We will employ a staged recruitment strategy of DPMs which will run in parallel with participant randomisation rates. The number of new DPMs recruited will be dependent on the number of lay mentors that continue from the feasibility programme. Therefore, recruitment of DPMs will not commence at month one of the study. If during the programme, withdrawal of DPMs is experienced, the recruitment will continue on a rolling programme to allow for cover of the withdrawals.

Training of DPMs

Newly recruited DPMs will receive 7 training seminars over a 7 week period prior to commencing their role. Training will be delivered by health care professionals of the Norfolk DPS team which has been piloted in the feasibility study. All training materials and session content have been developed and evaluated positively in the feasibility study by T2Trainers and staff members and will also be based on IMAGE toolkit and the US DPP materials. On successful completion of their training the DPMs will receive a Certificate of Attendance which is recognised by the University of East Anglia.

DPMs will be asked to provide two options:

- Time of day (for example: morning, afternoon or evening) for which they would be prepared and available to make telephone calls to the participants every week.

Prior to making their first telephone call to a participant each DPM will make a ‘practice’ phone call to either the Senior Intervention RA or a DPM Champion who will act as the participant. Feedback will then be given to the DPM based on this call.

Refresher training and further ‘practice’ calls will be given on a yearly basis to each DPM if the need arises. Practice calls will also be made using the DPM champion in replace of the DPM research associate if the need arises

NB. DPMs who have completed their contract in the Feasibility study and who continue into this RCT will receive 3 condensed specific refresher training sessions. This will ensure that they are familiar with any changes to the intervention since the feasibility study e.g. action plans and progress review sheets.

Telephone calls

DPMs will be assigned approximately 8 participants each based on 390 participants being randomised into the intervention (Project 2) and Project 4 (see below). The assignment of participants to DPMs will be completed by the Senior Intervention RA for the DPM area and the DPF team. Participants and DPMs will be matched initially according to gender followed by practicality factors such as days/times which are best for making/receiving calls as specified by each party.

Each participant will receive a 15 minute semi-structured telephone call from the DPM every 4 weeks during the education session phase (first twelve weeks) and then every 8 weeks in between their maintenance and physical activity sessions. When a telephone call is due the DPM will be sent their participant’s name, telephone number and latest diagnosis by the Senior Intervention DPM RA or assistant and they will also receive the participant’s latest action plan completed at their last Maintenance session. This action plan will form the basis of the phone call, with appropriate emphasis on physical activity and dietary behaviour.

NB. This action plan may be the same used for the previous call if the participant has not completed a recent action plan or attended their previous maintenance session.

Call structure

The first telephone call to a participant will be made after the participant has been randomised into the DPM arm of the study but before they attend their first Education session. Telephone call 2 will be made at the start of the education phase and subsequent calls will then be made every 4 weeks during the education session phase (first twelve weeks).

Following this, telephone calls will be made every 8 weeks in line with maintenance sessions. Taking into account the frequency of the telephone calls between the Education and Maintenace sessions, each DPM will make approximately 8 telephone calls per year/per participant and 6 calls per following years/per participant therefore, the DPM will make 22 phone calls per participant in the 40 month intervention. If a DPM is assigned the maximum of 8 participants this will equal approximately 176 calls in the 40 month intervention.

The Senior Intervention RA will mentor all DPMs involved through regular telephone calls and bi-monthly meetings. DPMs will have access to confidential phone numbers and follow the code of practice for confidentiality bound by their honorary contract with the Norfolk and Norwich University Hospital NHS Foundation Trust.

Each DPM will be given a BT charge card, which will be used for all phone calls to participants. DPMs will be asked to record the content of each phone call made using a standard telephone call record form (Document D22) and return it to the Senior Intervention DPM RA. They will be asked to record the following details:

- length
- time and date of phone call
- advice sought by caller and response given by DPM
- any concerns DPMs feel they need to identify
- if they require a supervision call from the Senior Intervention RA to discuss any aspect of the call (noted using a tick box)

The DPMs will only receive a supervision telephone call from the RA if they have ticked the appropriate box on the telephone call record.

Participants consent to any feedback from the Mentor call being passed back to the study team.

The Senior Intervention RA for the DPM area will receive itemised accounts for each charge card. DPMs will not incur any charges for calls made. DPMs will also be encouraged to use their BT charge cards when making any calls to the Senior Intervention RA.

DPMs will make a minimum of 3 attempts at contacting each of their participants. If, at any of the 3 attempts only an answer phone is available the DPM will leave a message stating when they will next call (based on their availability of 2 days given). Unanswered calls will be known as ‘blank calls’ and if no contact has been made with the participant after 3 of these blank calls the DPM will contact the Senior Intervention RA to inform them that they have been unable to contact the participant. The RA will make the decision to cancel the call and the participant will have to wait until their next scheduled call in 8 weeks.

Before completing each call the DPM will confirm the approximate date (week commencing) and time of the next call

Supervision of DPMs

The Senior Intervention DPM RA will mentor all DPMs through regular telephone contact and bi-monthly meetings.

Retention to the DPM Role

DPM volunteer retention in this arm of the trial is unlikely to differ from the retention level observed in the feasibility study which was recorded at 90%, 20 months after recruitment commenced.

DPMs will be awarded with a Certificate of Attendance from the University of East Anglia upon completion of their training seminars. A second Certificate will be awarded when they leave the study recording how many hours they volunteered in the role of a DPM.

Expenses

Any expenses incurred by the DPM relating to travel, parking or any other approved business in relation to the Norfolk DPS will be covered once authorised by the Senior Intervention RA and the DPMs will then be reimbursed by cheque. We recognise that the DPM role requires commitment and incurs some inconvenience therefore the DPMs will receive an honorarium payment of £350 pro rata at the end of the programme or as they leave.

Evaluation of the effectiveness of the DPM role

The DPM evaluation model will be based on the elements of the ‘national evaluation for the Expert Patients Programme’ and on a model already used in patient involvement in other curricula by Co-Investigator Professor Howe.

The model includes:

- profiling of applicants and those selected for training the feasibility and effectiveness of their training and outputs, measured by retention/dropout, hours delivered, and costs to the patient trainers.
- self-reported psychological outcomes measured by the WBQ-12, DMSES, DTSQ, (ADDQoL, DBQ, IPAQ, and various study specific questionnaires at months 0, 6, 12, 24, 36, 40 and 46
- Accelerometer measurements at 0, 6, 12, 24, 36, 40 and 46
- changes to physiological, clinical and biochemical measurements at months 0, 6, 12, 24, 36, 40 and 46 qualitative measures of acceptability, by focus groups with a selected number of DPMs and with participants at the intervention end measuring DPM role.

Qualitative Assessment

As for the feasibility study, the DPM role will be assessed qualitatively towards the programmes’ end by a series of focus groups with each party involved: up to 6 DPFs; DPMs (2 groups of 8); and participants (2 groups of 8).

The selection of DPM and participant groups will be purposive to ensure a broad cross-section of individuals.

To select DPMs to attend the focus group - the pre-recruitment questionnaire will form the basis of DPM selection.

For participants – we will apply a mixture of socio-demographics which were provided at registration (e.g. age, gender, and ethnicity) together with the latest fasting plasma glucose status as T2DM or IFG, plus any other emergent factors which need exploration. We cannot yet be confident about what these might be but the purposive sampling will allow for this.

The focus groups will last for approximately 60 minutes. In order to manage the focus groups effectively they will be run with one Research Associate and one Transcription Assistant. Structured prompts will be derived from the framework of Project 3, to gain an in-depth insight into the general views of DPMs; their training; their effectiveness within the intervention; and role satisfaction. Focus group interviews will be fully transcribed from the original recording. Framework analysis will be applied and discussed amongst the participating Research Associates [^48^](#_ENREF_48)^,^ [^49^](#_ENREF_49), and Professor Howe.

Quantitative Assessment

The effectiveness of volunteering as a DPM in the Norfolk DPS will also be measured quantitatively. Throughout their involvement in the study, DPMs will complete questionnaires (WBQ-12, ADDQoL, DMSES and DTSQ) and record body and biochemical measurements (BMI, weight, waist circumference, body fat mass, body fat percentage, HbA1c) to establish if these measurements alter in a positive way whilst acting in a mentoring capacity.

Blood samples will be taken by Senior Health Care Assistants (HCAs) or equivalent study staff member. The body measurements will be taken by HCAs and / or trained study staff at 0, 6, 12, 24 and 36 40 & 46 (exit) months.

Sampling Points

DPMs will have clinic appointments for body measurements at baseline (0 months) and at 6, 12, 24 and 36 40 & 46 (exit) months for blood tests and body measurements. The table below summarises data collection points.

| Outcome Measure | Baseline | 6m | 12m | 24m | 36 | 40 | 46 |
| --- | --- | --- | --- | --- | --- | --- | --- |
| HbA1c | ✓ | ✓ | ✓ | ✓ | ✓ | ✓ | ✓ |
| BP | ✓ | ✓ | ✓ | ✓ | ✓ | ✓ | ✓ |
| Height | ✓ | ✓ | ✓ | ✓ | ✓ | ✓ | ✓ |
| Weight | ✓ | ✓ | ✓ | ✓ | ✓ | ✓ | ✓ |
| BMI | ✓ | ✓ | ✓ | ✓ | ✓ | ✓ | ✓ |
| Waist circumference | ✓ | ✓ | ✓ | ✓ | ✓ | ✓ | ✓ |
| Body fat mass | ✓ | ✓ | ✓ | ✓ | ✓ | ✓ | ✓ |
| Body fat % | ✓ | ✓ | ✓ | ✓ | ✓ | ✓ | ✓ |
| ADDQoL | ✓ | ✓ | ✓ | ✓ | ✓ | ✓ | ✓ |
| WBQ-12 | ✓ | ✓ | ✓ | ✓ | ✓ | ✓ | ✓ |
| DSTQs | *✓* | *✓* | *✓* | *✓* | ✓ | ✓ | ✓ |
|  |  |  |  |  |  | ✓ | ✓ |
| DMSES | ✓ | ✓ | ✓ | ✓ | ✓ | ✓ | ✓ |
|  |  |  |  |  |  | ✓ | ✓ |
| IPAQ | ✓ | ✓ | ✓ | ✓ | ✓ | ✓ | ✓ |
| Q18 (resistance training questionnaire) | ✓ | ✓ | ✓ | ✓ | ✓ | ✓ | ✓ |
| Accelerometer | ✓ | ✓ | ✓ | ✓ | ✓ | ✓ | ✓ |

PROJECT 4. Intervention in screen detected T2DM.

In this 40 month project we hypothesise that the Norfolk DPS intervention in screen detected T2DM participants will improve glycaemic control (HbA1c) compared to a control group, and that using a DPM will further significantly improve this outcome (if randomised to the intervention + DPM).

We will randomise 240 screen detected participants from Project 1 to; DPS intervention without DPM input, DPS intervention with DPM input or a control group. In addition to the programme’s intervention all participants will receive standard local best practice care (independent of the Norfolk DPS) for newly diagnosed T2DM patients which includes; sessions with a senior diabetes specialist nurse, podiatrist and community dietician.

A letter including their blood results will be sent to the participant’s GP (Document S27). This letter will also ask the GP to book them into the standard diabetes care pathway and consider referral into the newly diagnosed T2DM education programme.

The intervention sessions will be provided in mixed groups of IFG and T2DM participants (Projects 2 and 3), these groups will also be split by randomisation group with those receiving DPM support enrolled in speared sessions to those without DPM support. This project is largely cost neutral and tests the value of our intervention in screen detected T2DM as an off the shelf lifestyle programme.

Confirmed T2DM

**320**

256 assuming 80% consent to intervention - **290**

+ 34 from pilot

Control one off education session

**107**

Norfolk DPS intervention with DPM

**107**

Norfolk DPS Intervention without DPM

**107**

Attendance at group education session and standard care for T2DM

Norfolk DPS intervention without DPM and standard care

Norfolk DPS intervention with DPM and standard care.

Figure 5 – Flow diagram showing the study design and interventions for participants with T2DM.

Assessment

Participants randomised into Project 4 will also be asked to complete the DTSQ and DMSES at month ~~40~~ 46 (exit point)

PROCESS EVALUATION

Process evaluation will be an integral part of the trial to give greater explanatory power to the outcome measures and to provide a better understanding of the mechanisms of action and likely generalisability of the intervention. A detailed protocol for collection and analysis of quantitative and qualitative data for the process evaluation is being developed by the study’s behavioural science team. The main elements of this are outlined below. The process evaluation will follow the recommendations of the MRC framework for evaluation of complex interventions [^50^](#_ENREF_50). Based on the theoretical model and approaches underpinning the intervention as described above, we will identify key components of the behaviour change processes targeted by the intervention to provide a basis for a) assessing intervention fidelity (the extent to which the intervention is delivered as intended) and b) measuring explanatory variables to demonstrate the proposed mechanisms in action and increase understanding of the processes which underpin behaviour change and their links to clinical outcomes in this type of intervention.

Higher level process analysis

Change in diet

Change in activity

Diabetes incidence

Changes in risk factors: weight, cholesterol, BP, insulin sensitivity

Figure 6 - Physiological/causal model for diabetes prevention

At the highest level changes in dietary and physical activity behaviours will be related to outcomes as per figure 6 shows. We will use the study’s clinical outcome measures and measures of physical activity (accelerometer data, IPAQ) and diet (the DBQ Questionnaire) to assess the relative contribution of these behaviours to diabetes risk factors and incidence. It will be difficult to accurately assess the contribution of dietary change but this could be estimated by assuming that the amount of risk factor change not accounted for by physical activity is accounted for by dietary change (this will provide an upper limit at least). The importance of different aspects of physical activity and dietary behaviour will also be explored.

Intermediate level process analysis

**Intervention Fidelity**

**Change in diet**

**Change in activity**

**Attendance Rates**

Figure 7 - Intermediate level process model for behaviour change

Intervention fidelity (i.e. the quality of intervention delivery) is theorised to cause changes in diet and physical activity, but this effect will be mediated by participant attendance at intervention sessions, which will be recorded (Figure 7). Intervention fidelity will be accessed via a) recording and transcribing a sample of DPF sessions to provide qualitative data for analysis; and b) an observer quantitatively scoring delivery quality (both content and style) across a range of DPF intervention sessions using a checklist which will be adapted from existing sources by the behavioural science team. These approaches will also allow important aspects of interactions between DPFs and participants, and group dynamics which may affect delivery, participant engagement and intervention effectiveness, to be captured.

Assessing the fidelity of the intervention is of particular relevance given that the ‘same’ intervention will be delivered by multiple DPFs across a number of sites. At a basic level, subgroup analyses will examine differences in the effectiveness of the intervention on primary and secondary outcomes across DPF group and intervention site. Variations in intervention delivery accounting for these will then be explored. Intervention attendance and retention rates for participants and measures of intervention fidelity will be modelled (e.g. using regression analyses) against clinical and behavioural outcomes to test the proposed mechanisms underlying efficacy of the intervention.

Further insight into aspects of intervention fidelity, and in particular, ways in which this might be enhanced for future delivery (e.g. how the training and intervention delivery can be improved) will also be gained via focus groups with DPFs, and participants with IFG and diabetes and selected individual interviews with participants. These will allow qualitative exploration of, for example, views on barriers to/facilitators of intervention delivery and what works best/worst about the intervention.

Finer-grained process analysis

The validity of the HAPA model outlined previously as the theoretical basis for behaviour change in the intervention will be tested by measuring (a) intra-individual variables, such as changes in cognitions related to diabetes risk, diet and physical activity, and (b) intervention-process variables related to the degree of engagement of participants with intervention processes, such as action planning and self-monitoring activities. The variables to be assessed will directly represent the key components of the HAPA model postulated to underpin behaviour change (e.g. perceived risk, self-efficacy, intention). With expert input from one of the originators of the HAPA model (Falko Sniehotta) the behavioural science team is developing a questionnaire to assess many of these variables using existing standard items where available. Relevant quantitative and qualitative data will also be extracted from participant’s action plans completed as part of the intervention. Quantitative measures will be used to model the extent to which the intended cognitive variables and intervention processes mediate behavioural outcomes, using either regression analyses or structural equation modelling if sample sizes allow. Focus groups with participants (those with IFG and diabetes), DPMs and DPFs and interviews with participants, in addition to exploring intervention fidelity as outlined above, will also be used to explore experiences of delivering and receiving the intervention and to shed further light on the proposed mechanisms of behaviour change. As well as using the focus group and interview data to further test the pre-defined model, analyses will be undertaken to allow for the possibility of novel insights from the qualitative data.

Collateral effects of intervention on DPMs

Finally, another process of interest is the possible co-lateral effect of the intervention on the DPMs. Data from other studies suggests that volunteering in health promotion projects confers benefits on the volunteer as well as the recipient. In this case, we have hypothesised that the DPMs may use the intervention materials and processes to change their own diet and physical activity behaviours (as these are relevant self-care issues for people with type 2 diabetes). We will explore the processes by which such co-lateral benefits may occur through the use of focus groups with DPMs and the use of selected quantitative process questionnaires (e.g. to measure changes in behaviour and self-efficacy about diabetes management), and clinical measurements (e.g. BMI, weight, body fat mass, body fat percentage and waist circumference) assessed at baseline and follow up assessment points. These will be related to the health outcomes measured for this group (see primary outcomes section).

Summary of process evaluation

The various components of the process evaluation will shed light on *how* the intervention works, will enhance understanding of *how* changes in diet and ~~.~~or physical activity can best be supported and will help to explain variation in outcomes between individuals irrespective of whether the intervention is found to be effective overall. The bulk of the data on process variables will be collected from participants assigned to the intervention groups in studies 2, 3 and 4. Quantitative data will be obtained from a brief questionnaire (e.g. to assess cognitions related to diet and physical activity) as well as from forms completed as part of the intervention itself (e.g. covering confidence about achieving changes in behaviour, participants’ action planning). Versions of the questionnaire will be administered at baseline, at the end of the last core education session months, 12 months, and at 24 months.

Furthermore, qualitative data will be derived from the recording and transcribing of intervention sessions, from focus groups with participants, DPFs and DPMS, and face-to-face interviews with a sub-sample of intervention participants. All participants will be asked at the consent stage if they are willing to have sessions in which they are participating recorded and to be approached for focus groups and qualitative interviews. We will then purposively select a sample to approach based on maximising variation in demographics and weight loss achieved. Interview and focus group topic guides will be developed to allow exploration of processes of behaviour in general, and in light of the particular model being tested. Questions about the participants’ experiences of taking part in the study/receiving the intervention, and DPFs’ and DPMs’ views on delivering the intervention will also be included. Analysis of the qualitative data will aim to provide a longitudinal narrative of change processes, using template analysis (a form of Framework analysis) or similar methods to examine the extent to which the data fit with or deviate from the proposed theoretical model (King, 1998). In particular, participant data will be compared and contrasted between participants who achieve or do not achieve behaviour change targets for diet and /or physical activity. Data from the intervention providers (DPFs and DPMs) will be used to compare different perspectives about how the intervention works (and how it could be improved) and (along with the data from referring GPs) to acquire further information about barriers to and facilitators of intervention delivery from the providers’ perspective.

A mixed methods study of processes of behaviour change initiation in adults at risk of developing type 2 diabetes

Little qualitative research has been conducted to find out exactly what does and does not motivate people to initiate a change in their health behaviour and what barriers they face at an individual level, as well as whether these differ between different groups in society. It is this information that is needed to pass on to public health professionals so that interventions can be effectively designed to meet the needs of the target population. There is a lack of research in general about the barriers and motivations for behaviour change in populations identified as at risk of a chronic disease (such as T2DM). These are likely to differ from a general population due to the more immediate risk to their health, which is also likely to impact on cognitive and particularly emotional processes involved in behaviour change. However, in a study on diabetes risk perception and intention to adopt a healthy lifestyle, participants who recognised that they were at high risk for diabetes did not have greater intentions to change their behaviours than participants with a low perceived risk (Hivert, Warner, Schrader, Grant & Meigs, 2009). Fisher and colleagues (2002) recommended that more research on the impacts of the 'at risk' for diabetes notification on psychological, behavioural and social factors is needed. Most of the studies that have investigated barriers and motivations for behaviour change have focused largely on physical activity changes, rather than diet alone or diet and physical activity. Recommendations in a paper by Wing and colleagues (2001) included research to increase the understanding of motivation for the initiation of dietary and physical activity behaviour change. There are benefits of both quantitative and qualitative methods for examining motivations and barriers to behaviour change, but as yet there are no studies using mixed methods to investigate this in adults with prediabetes. In addition, some literature has noted a difference between self-efficacy and level of behaviour change for different health behaviours (such as physical activity and diet) (Absetz, Valve, Oldenburg et al, 2007; Leventhal, Weinman, Leventhal & Phillips, 2008; Sniehotta, Dombrowski, Avenell et al, 2011; Ziebland, Thorogood, Yudkin, Jones & Coulter, 1998) and between men and women (Absetz et al, 2007; Sherwood & Jeffery, 2000; Ziebland et al, 1998), but this has not been examined with regards to physical activity and dietary behaviour change in a population identified as having an increased risk of developing T2DM. Working alongside the Norfolk DPS, this PhD project will help to fill these gaps in the literature. This PhD project will have a dedicated Participant information sheet, consent form and participant invitation letter.

A random sample of intervention participants with prediabetes will be invited to take part in an interview looking at the initial processes of behaviour change in people at risk of developing T2DM. This will include an in-depth exploration of the motivation phase of the theoretical model used and will also investigate the participants' views of the Norfolk DPS core education sessions, which will be used to supplement the evaluation of this aspect of the programme. Specifically, the main topics to be questioned will cover participants' motivations and barriers to making the lifestyle changes recommended in the intervention sessions; confidence in their ability to change their behaviour; which behaviours they found the easiest and most challenging to change and reasons why; and their understanding and use of the model of behaviour change being used in the intervention sessions. All potential participants will be given a participant information sheet and a consent form specific to the interviews during a selected number of Education Session 5s and the PhD student will telephone all attending participants after 72 hours to give further information and gain verbal consent for taking part for those who wish to. Consent forms will be signed in person on the day of the interview before it begins. Approximately 20 interviews will be held, with recruitment continuing until a saturation point in terms of the ideas being discussed appears to be reached. Both males and females will be invited to take part in order to specifically explore any gender differences in the behaviour change process. The interviews will run in the time between the end of the core education sessions and the beginning of the maintenance sessions. The PhD project will also use the quantitative questionnaire data from participants with prediabetes in the intervention groups to explore the predictors of initial behaviour change and the validity of the theoretical model.

Exploring participant perspectives on key areas on the intervention sessions, programme positives and negatives.

It would be beneficial to explore the interpersonal interaction and group processes in more detail, as this seems to be an important factor mediating health related behaviour change and one that is currently poorly understood. In particular, we will try to identify group processes that occur in the groups and explore how they mediate the use of behaviour change techniques that comprise the NDPS intervention.

This additional work will allow us to

a) Better understand the influence of group setting on intervention outcomes

b) Identify successful intervention components and change techniques

c) Understand the challenges related to the group mode of delivery, and contribute to future training and courses for the group facilitators.

We will to extend our understanding of the intervention delivery process, in particular the role of the interactions between participants and between participants and the Diabetes Prevention Facilitators (DPFs). Interviews with group session participants and facilitators will provide us with valuable feedback about the experiences and perceptions of participating in and facilitating the groups, and how the group setting itself might influence behavioural change and intervention outcomes.

25 participants from the two intervention groups (i.e. with and without Diabetes Prevention Mentors) and all of the DPFs will be recruited for semi-structured interviews. They will be recruited as soon as possible after completing the six educational group sessions. Their facilitator will give the participant a letter with an invitation to the interview, Participant Information Sheet, Consent Form, and a Reply Slip with a pre-stamped and pre-addressed return envelope. Participants who have already been interviewed as part of the existing process evaluation will not be interviewed again. We aim to recruit 25 participants from different groups, and will attempt to recruit approximately equal numbers of men and women. Those participants who agree to participate, by return of the reply slip, will be contacted by the PhD student to discuss the study, and will be given the opportunity to ask questions. Prior to the interview the participant will be asked to sign a consent form. In the first instance, the participants will be interviewed via the telephone. If the quality of the data collected via the telephone interview is deemed insufficient quality, the participant will be interviewed face-to-face at a convenient venue. The interviews will take about 60 minutes, and will be audio recorded and transcribed. The data will be anonymised, confidential, and will be stored securely on a password protected laptop and external hard drive. The content of the interviews with the participants will include their experiences of the groups, what was useful or not, interaction with other participants and with the facilitators, group activities, and materials used during the sessions. Interviews with the facilitators will focus on their experiences of working with groups, facilitating group sessions, and training in group facilitation skills. Participants’ and facilitators’ understanding of how groups work and how they can be used effectively to influence lifestyle changes, such as changes in diet and physical activity, will be also explored. This PhD project will have a dedicated Participant information sheet, consent form and participant invitation letter.

STATISTICAL CONSIDERATIONS AND SAMPLE SIZE

Project 1

Our average screening capacity over 2 years is approximatley 100 per week for 50 weeks per annum allowing for internal cover. We will identify 570 IFG participants (estimated IFG prevalance 5.7% in those aged 40-74 with BMI ≥ 30) based on GP mailshots. The pathways between pharmacy screening and the programme will identify a further minimum 509 potential IFG participants based on current activity alone over two years in 11 large pharmacies, and laboratory confirmed clinically detected IFG participants during screening period (excluding those detected previously). We will assume that 15% of these participants (who have already self-volunteered for glucose testing or have confirmed IFG and where 80% consented in our feasibility study) will consent to screening in this programme. This will identify 475 IFG participants over 2 years, which together with the general screening sample (n= 570), gives 1045 IFG participants. Assuming 80% progress to randomisation (as in feasibility) this gives 836 randomised subjects. In addition 75% of 177 will have been randomised in the feasibility programme, giving a maximum potential randomised sample of 1055. This in turn will give a randomised sample size of 950 if we assume a 5% drop out as in the feasibility programme and 10% of those with HbA1c ≥48 mmols/l have T2DM from an OGTT (52).

Projects 2 and 3.

Effect sizes for Project 2 are based on a Meta analysis effect size of 0.51 (95% CI 0.44-0.66) of diet and lifestyle interventions in IGT (19-23). The further Project 3 effect size is made on the assumption that the added DPM intervention will enhance weight loss, weight loss maintenance and exercise adherence, which was poor at DPS end [^16-20^](#_ENREF_16), and where each 1 kg weight loss was equivalent to an 18% reduction in T2DM incidence [^16-20^](#_ENREF_16). The lowest quartile dietary fat intake subjects in the Finnish Diabetes Prevention study [^51^](#_ENREF_51) had half the T2DM incidence compared to highest quartile, with an overall mean 58% reduction. It is reasonable to test our assumption that the Project 3 effect size will further reduce IFG transition rate to that described in a white IFG moderate risk population at 2.5% (1.9-3.3) after some lifestyle intervention [^52^](#_ENREF_52).

Mean patient follow-up is 3.25 years (newly recruited plus feasibility) - this combined with the fact that we will using a more powerful analysis technique (using the time-to-onset of diabetes) indicates this to be a conservative estimate of power for Projects 2 and 3.

Data analysis and general statistical analysis for Projects 2 and 3.

For Projects 2 and 3 we will use an intention-to-treat approach. For binary outcomes we will use the chi-squared test or logistic regression if adjustment for baseline imbalances is required; for continuous outcomes we will use the t-test or analysis of covariance.

For time-to-event outcomes the proportional hazards model will be used with time measured from randomisation until the end of the study or drop-out (censoring) or diabetes is diagnosed.

Secondary analyses will include a per-protocol analysis and analysis of secondary outcome measures. For determinants of progression we will use logistic regression and the proportion hazards models with the time of the onset of T2DM as the outcome measure in each treatment arm separately as well as compliance with the intervention. If necessary we will use multiple

imputation [^53^](#_ENREF_53)to assess sensitivity to missing data. All analysis will be performed in STATA 11.

*Project 4* has 80% power to detect a 0.4 SD difference in HbA1c (estimated 0.7% between groups), which is biologically and clinically significant (68) and will require minimum size of 71 participants in each arm and we will randomise 80 to each group to maintain power for 5% drop out.

PROJECT 5 Economic evaluation of Projects 1- 4.

There will be three components to the health economic analyses in this programme: i) efficiency of different IFG screening methods, ii) the cost-effectiveness of the lifestyle intervention with and without DPM support, and iii) economic modelling of long term cost impact of the Norfolk DPS intervention.

Economic evaluation of IFG screening programme.

The economic analysis will estimate the overall cost associated with the IFG screening programme. A detailed cost breakdown for each recruitment strategy will be calculated with identification of relevant resources used (time inputs by practice staff, consumables etc), measurement of the quantity of these resources, and valuation. The cost per participant screened will be estimated for each recruitment method. The value of providing a targeted screening programme for IFG/T2DM will also be explored via the willingness to pay technique (WTP). The short WTP questionnaire in the feasibility study was framed in an open-ended format, and will be used to inform suitable anchors used to apply a closed-ended (payment scale) format. This questionnaire will be administered to all participants

Economic evaluation of Norfolk DPS lifestyle intervention.

We will estimate the cost-effectiveness of the Norfolk DPS (with and without DPM support), compared to the control arm, for individuals identified as having IFG, or T2DM. To estimate NHS costs, we will monitor resource use in the programme.

Resource use associated with the intervention includes:

- the provision of advice to controls (standard care)
- DPF contact time with participants (including preparation time, leaflet/other material, sites, contact time, number in group)
- costs associated with DPM (derived from British Telecom Charge cards; volunteer time, and staff costs for the Senior Intervention DPM RA).

These will be monitored in conjunction with other services that are potentially related to the intervention (e.g. referrals to dietician and GP visits, hospital stays, medication use). Appropriate unit costs will be assigned to these resource items and participant borne costs will also be estimated. The economic evaluation will use the EQ-5D, a generic measure of health status as the main measure of effectiveness [^54^](#_ENREF_54)^,^ [^55^](#_ENREF_55).[^56^](#_ENREF_56)

All participants will be asked to complete the EQ-5D questionnaire at 0, ~~4~~, 6, 12, 24, 36, 40 and 46 months, enabling the QALY (quality adjusted life year) gain to be estimated.

Costs incurred in future years will be discounted. Subsequently, the Incremental Cost-Effectiveness Ratio will be calculated from a health service (base-case) and a societal perspective, and compared to a range of cost-effectiveness thresholds. In order to characterise the level of uncertainty the cost-effectiveness acceptability curve and cost-effectiveness acceptability frontier will be presented.

Sensitivity analysis will also be undertaken to assess the robustness of conclusions to key assumptions that are made within the economics analysis.

Longer term economic modelling of the Norfolk DPS data.

An economic model of T2DM prevention will be developed to extrapolate cost and effects on a remaining lifetime basis. The model will be data-driven from the Norfolk DPS programme and past trials and performed by an NIHR Research Methods Fellow (commenced August 2010) under the

direction of applicants Niessen, Bachmann and Sampson. We will construct a multi-dimensional probabilistic life table-based model, by one year age groups and sex, which will include glycaemic state (no diabetes, IFG, T2DM), and disease states including diabetes micro- and macro- complications over the remainder of each participant’s lives, grouped by programme arm. The short-term model parameters of the life table will be estimated from the trial itself (prevalance of complications and existing disease, progression rates, intervention costs), while the remaining will be obtained from elsewhere (e.g. diabetes-related mortality, excess mortality rates, utilities in each state, and cost of complications).

The various risk functions such as the UK Prospective Diabetes Study (UKPDS) for long term and cardiovascular outcomes equations will be compared, tested, and calibrated against key model parameters (diabetes mortality, incidence and prevalence) [^56^](#_ENREF_56).

We will use life tables to describe the East Anglia diabetes population and these will be validated against other external parameters available, such as overall mortality by age and sex, prevalence of diabetes complications and health care utilisation and cost estimates.

Individual data for each trial participant will be entered in a Monte Carlo simulation, generating a new data set of patient records with extrapolated data on patient survival (life expectancy, health-adjusted life expectancy, lifetime health care costs). These model outcomes will be used to estimate the long-term cost-effectiveness of the intervention programme (incremental cost-effectiveness ratios and confidence intervals, acceptibility curves and 90% confidence ellipses). This will be done through multi-variate regression analyses, accounting for possible cluster-effects, cluster characteristics, and uncertainty in the long-term outcomes. This element will be lead by applicant Niessen.

ENDPOINTS

The primary end point for particpants diagnosed wi~~r~~th IFG and IFG_ADA_ will be:

- progression from IFG/ IFG_ADA_ to T2DM in the Norfolk DPS intervention group, compared to the control intervention at study end.

The diagnosis of T2DM will be made on paired fasting glucose measurements both ≥7.0mmol/l, or a 2 hr OGTT ≥ 11.1 mmol/l undertaken if HbA1c ≥42mmols/l in IFG participants.

Secondary end points:

Project 2 and 4

The six prespecified secondary end points in this project are:

- changes in Homeostasis Model Assessment (HOMA) estimates of insulin sensitivity and B cell function (60) based on FPG and insulin levels,
- changes in exercise levels, based on; accelerometer data, self reported activity and IPAQ questionnaire
- changes in dietary intake based on self reported DBQ questionnaire
- changes in weight, body fat mass, visceral fat, BMI and waist circumference,
- changes in EQ–5D, WBQ-12, item 1 from the ADDQoL, and study specific questionnaire data.
- development of a weighted composite lifestyle change score based on changes in weight, BMI, waist circumference and exercise levels.

Those participants randomised into Project 4 will also complete the DTSQ and DMSES questionnaire at 6, 12, 24, 36, 40 and month 46 (exit) time points

These end points will be analysed between and within groups at the end of the programme and determinants of these changes analysed.

Project 3

For Participants

The primary end point will be the progression of IFG to T2DM in the Norfolk DPS intervention with DPM group (n = 390), compared to the Norfolk DPS intervention alone (n = 390).

The six pre-specified secondary end points are as in Project 2 and 4 above.

For DPMs

Primary end points are:

- changes in HbA1c levels

Secondary end points are:

- DPM biometrics (weight, BMI and body fat, at specified time points) as DPM themselves may experience better outcomes in terms of HbA1c and weight loss
- changes in exercise levels, based on; accelerometer data, self reported activity and IPAQ questionnaire
- changes in dietary intake based on self reported DBQ questionnaire
- DPM quality of life and psychological well-being measured by WBQ-12, DMSES, ADDQoL and DTSQ as individuals who provide social support through volunteering experience less depression, heightened self-esteem and self-efficacy, and improved quality of life, and improved health outcomes [^57^](#_ENREF_57), the DPMs perceptions of their capacity to deliver the Norfolk DPS intervention (months 48-54),as measured by focus groups .
- DPMs confidential views on DPM intervention efficacy in a study specific questionnaire and focus groups

Project 4

The primary end point is the difference in mean HbA1c at 46 months between groups, and Quality and Outcomes Framework (QOF) scores for diabetes based on the diabetes QoF dataset between and within groups at study end. The pre-specified secondary end points are those descibed above. Questionnaire measures are the same as for project 2 but in addition, DBQ, DTSQ, DMSES, and ADDQoL, EQ-5D and WBQ-12. The DTSQ, ADDQoL, and DMSES will be measured at 0, ~~4~~, 6, 12, 24, 36, 40 and 46 month timepoints and will not be measured at baseline (0 months).

TRANSITION OF FEASIBILITY PROGRAMME PARTICIPANTS TO FULL PROGRAMME

Existing participants with IFG or T2DM in the feasibility programme; 118 (n = 90 IFG, n = 20 T2DM) intervention, and 59 (n = 39 IFG, n = 20 T2DMs) control participants will be written to with a PIS inviting confirmation into the Norfolk DPS after re-consenting. If they agree to participate, those participants who in the UEA-IFG Study were randomised into the intervention group will receive maintenance sessions and motivational phone calls only. Nested cohort intervention+DPM participants will not be asked to participate in the education sessions. These participants will attend up to 15 maintenance sessions and DPM calls will commence after the first maintenance session. Those participants who, in the UEA-IFG Study were randomised to the control arm will receive a one off education session. Feasibility participants will be required to remain in their same groups.

Participants who continue into the Norfolk DPS will have clinic appointments at 0, ~~4~~, 6, 12, 24, 36, 40 and 46 months where fasting plasma glucose, HbA1c, Insulin and lipid profiles will be tested. IFG Participants (without a previous confirmed T2DM result in the feasibility study) who develop T2DM at baseline (0 months) will continue in their original group. IFG participants who develop T2DM after baseline (0, ~~4~~, 6, 12, 24, 36, 40 and 46 months) will be given the choice to continue in their original group but will not be required to attend further Norfolk DPS clinic appointments. Instead, we will ask partcipants’ GPs to provide us with details of annual blood tests. The final data for these particpants will not be analysed with the Norfolk DPS data as the cohorts are not comparable.

ATTAINMENT OF PRIMARY END POINTS

Participants in the IFG intervention study (390 with intervention and DPM, 390 intervention and without DPM and 170 controls) are at risk of developing the primary end point of type 2 diabetes. This is estimated as approximately 24% over 3 years in the control group (n = 41), 12% in the intervention arm without DPM (n = 47) , and 6% in the intervention arm with DPM (n = 23). This is a total of 110 primary end points of type 2 diabetes. Participants with IFG who develop the primary endpoint of type 2 diabetes will be able to continue to attend maintenance sessions and clinical data will obtained from their GP practice. All participants entering the programme give consent on the understanding that if they develop the primary endpoint we would have consent to obtain routine diabetes care data from their practice. We will also continue to collect observational data and will ask participants to complete annual questionnaires capturing self-reported health outcomes and health resource data.

SCREENING LOG

A screening log for participants recruited at sites will be held securely and confidentially. The log will include a list of the reasons for non-inclusion.

PROGRAMME CLOSURE

The programme will cease at the end of the commissioned period. A summary of the findings will be disseminated to all the participants and include post-programme guidance on next steps. This guidance will be determined by programme outcomes, and we will ask for REC advice and review of proposed communications. If the Norfolk DPS intervention is successful, we plan to seek additional funding and ethical approval to enable the participants in the control group to benefit. We will communicate the results of the Norfolk DPS to all participants, GP practices and to local media.

PARTICIPANT WITHDRAWAL OR EXCLUSION

Participants or DPMs are free to withdraw from the programme at any time and do not have to give a reason. At any point during the programme the Chief Investigator and/or Principal Investigator may withdraw a participant or DPM if it is felt the programme is no longer of benefit to them or it is no longer in their best interest to take part. During the programme, if any participant develops a condition which would have initially excluded them, this will be discussed with the Principal Investigator/Programme Manager in the first instance and a decision by the Chief Investigator will be sort.

PROGRAMME MONITORING

Access to data

The programme database will be password protected and any personal data held on computer will be kept confidentially and only be accessible to the members of the Norfolk DPS team. Any hard copies of documentation including personal data will be kept in locked filing cabinets in the Clinical Trials and Research Unit (CRTU) at the University of East Anglia (UEA) or an alternatively assessed site and will only be accessible to the research team. The emergency de-coding procedure for the Norfolk DPS will be conducted in accordance with GCP and the JRO SOP 835 (Emergency Code breaking procedures).

Confidentiality

Where possible, personal data of participants will be made anonymous and will otherwise only be accessible to relevant personnel in the research team involved in delivering the intervention. Any personal identifiers will be removed prior to publication.

Quality Assurance/Audits/Quality Control of Documents

The study will be fully GCP compliant and will be audited by the Research and Development Department at the Norfolk and Norwich University Hospital NHS Foundation Trust. The programme will receive formal quality assurance support from the Norwich CTU including the documentation of all quality procedures in a Quality Management Plan and assistance to set up the TSC and DMC with roles and responsibilities of these committees documented in line with established guidelines.

Independent data monitoring committee (DMC).

An independent data monitoring committee will meet regularly to review study data. This committee will determine the form of data analysis, end point adjudication and interim analyses to be undertaken.

Strategic trial steering committee.

This will be chaired by an independent external chair. This committee will be responsible for overseeing quarterly activity and activity Work Package summaries from 4 operational groups (screening group, Norfolk DPS intervention group, DPM Intervention group and health economics group).

Co-Investigator research team meetings.

Will be held at bi-monthly intervals.

Participant Advisory Board (PAB) (Lay members)

The views of the public and patients are essential in the success of research studies and it is important that these views are represented during the design and conduct of a study. Lay members on the PAB for the Norfolk DPS will play a key role representing the participants in the programme and will be involved in reading, reviewing and providing feedback on any documentation that will be given to participants in the programme. Lay members appointed to the PAB will be required to sign a consent form agreeing to become a member of the Board.

The expected duration of this role is until programme end. PAB meetings will take place every 3 months and the chair of the Norfolk DPS Participant Advisory Board (PAB) will be asked to attend the bi-monthly co-investigator research team meetings. The Board will be chaired by Co-investigator Rea the Norfolk Diabetes Patient Champion for type 2 diabetes, Norfolk Integrated Diabetes Management (NIDM) and initially two lay members will be recruited eventually recruiting up to eight members. These lay members will be DPMs and participants from the UEA/IFG Feasibility Study. The chairperson will receive an honorarium payment of £350 paid pro rata at programme end or on leaving the programme.

SAFETY REPORTING

We have robust mechanisms and pathways for Adverse Event reporting which will be reviewed as a standard item by the programme operational group, with the CI and PI.

All staff working on the Norfolk DPS will be trained to record participant adverse events, defined as:

Serious Adverse Events (SAE) – An untoward and unexpected outcome that:

- results in death
- is life threatening
- requires hospitalisation or prolongation of existing hospitalisation
- results in persistent or significant disability or incapacity
- is otherwise considered medically significant by the Chief Investigator (CI) or Medical Advisor.

Adverse Event (AE) – An untoward and unexpected outcome, which does not fall within the definition of a Serious Adverse Event, but is considered to be medically significant or having an impact on participant role within the Norfolk-DPS programme e.g. extreme and concerning weight loss; bouts of headaches; or general illness e.g. flu-like bugs; vomiting etc., and any illness which is felt by the participant to be related to the Norfolk DPS.

Events will be recorded on study adverse event forms and database. For SAEs, additionally, details will be forwarded to the sponsors following standard procedures.

Risks and benefits.

Potential Risks

1. Potential discomfort and bruising due to venepuncture which are minimised by using staff trained to take blood.
2. It is recognised that some people will be shocked to receive a diagnosis of either IFG or more especially T2DM and therefore these people with newly diagnosed T2DM will be offered the opportunity to discuss their results with medical staff (research GP or Consultant).

4 As with all physical exercise there is a risk of common injury, this will be minimsed by

encouraging gradual increase in exercise levels which will be supervised and monitored

by a trained and suitably qualified physical activity falcilitator in the maintenance sessions. Following completion of the pre-participation health questionnaire, if required this will be referred to the participant’s GP for advice prior to their participation in the physical exercise

sessions.

Potential Benefits

1. Participants who adhere to recommended lifestyle changes may accrue significant physical benefits in terms of overall health awareness.
2. For the intervention group, access to DPFs, physical activity falcilitator, DPMs and other participants has potential to act as a social network support system to aid lifestyle changes for the duration of the programme and possibly beyond.
3. Early detection of IFG and T2DM will allow early treatment intervention.

ETHICAL CONSIDERATIONS

Equipoise and potential for harm

a) Although we hope that the interventions will improve outcomes, we do not know this, and we need to undertake large randomised controlled trials to test this. Participants assigned to control groups, (not assigned to receive the novel interventions being evaluated) will receive standard best care through the NHS. It is important to stress that there is no validated tested diabetes prevention programme available for application in the UK outside intense research settings. In addition, there is no validated structured diet and lifestyle programme for screen detected T2DM, and no commissioned structured diet and lifestyle education programme for people with type 2 diabetes in Norfolk, but all screen detected T2DM patients will have standard best practice diabetes management and education. All interventions being evaluated are based on health promotion principles and do not entail changes in medical care, which will continue to be provided by their usual health professionals. It is therefore ethical to maintain a control group in these projects while testing this intervention.

b) One potential harm to participants is the permissive effect of a normal fasting glucose, and the incorrect assumption by participants and Health Care Professionals that this risk ceases or is diminished [^58^](#_ENREF_58)^,^ [^59^](#_ENREF_59). We will ensure that the PIS and all materials stress and quantify this continued risk.

c) Biomedical approaches to screen detected T2DM emphasise health benefits of early detection, and screen detected T2DM may be associated with *less* psychological morbidity, but the potential adverse consequences of screen detected T2DM have been well reviewed [^58^](#_ENREF_58). We will underpin all approaches to all populations with standard assessments of QoL and psychological morbidity, and all screen detected T2DM patients will be offered the opportunity to discuss their diagnosis with programme clinicians and will be entered into standard local best practice pathways.Those with IFG who decline to participate in the programme will be advised to have an annual screening at their GP practice.

d) Data relating to ethnicity will be collected on a voluntary basis at initial screening. This

information will be required for prevalence.

Informed consent

Participants will be provided with the Participant Information Sheet for the Norfolk DPS or the HbA1c Observational study and will have had the study explained by a member of the team and had the opportunity to ask questions prior to written informed consent being

taken. Informed consent will be taken from potential participants prior to the screening

procedures being undertaken and prior to randomisation into the intervention or control

groups. Written informed consent will also be obtained from the Diabetes Prevention Mentors

(DPMs) prior to them commencing their role.

Confidentiality

a) We are aware of the confidentiality issues which occur in group working, particularly when

delivered by members of the public. The DPMs will all have honorary contracts with Norfolk

and Norwich University Hospitals NHS Foundation Trust (Directorate Diabetes and

Endocrinology), DBS checks, and training in confidentiality issues. All DPMs will be asked to

sign a consent form to take part in the Norfolk DPS and the NNUHFT policy for confidentiality

will be included in their induction pack.

b) All research staff will have received training in (GCP) in conjunction with all NHS mandatory training, and will be aware of issues of confidentiality and the appropriate storage of study data. Where possible participant information will be anonymised by code number. Within the participant information sheet it will be made clear that data will be shared with the participant’s GP where appropriate. Additionally, within the participant information sheet and at the start of sessions it will be made clear that some elements of confidentiality cannot be guaranteed during group sessions since participants could potentially discuss data outside of the group. During group sessions, the use of a facilitator will assist all participants in making a contribution to discussions if desired and the DPFs will be trained to encourage participant views and avoid the use of leading questions.

c) Data will be stored in a secure database on the CTU server at the University of East Anglia.

The database uses Microsoft's SQL Server system. Access to the server is restricted absolutely to

CRTU data management staff.

d) Access to the database is protected by usernames and passwords. The server and its contents are covered by the UEA IT regulations and policies. Data on the server is backed up nightly and regularly archived to off-site storage. In addition, other 'quick-access' backups will be taken by the CRTU Database Manager and held on-line to facilitate rapid recovery of recently changed data.

e) Data entry and day-to-day data management will be performed by Norfolk-DPS employees via a dedicated secure website. Each user will have an individual username and password without

which they will not be able to access the system (any attempt to access pages 'deeper' into the
Norfolk-DPS data entry system will automatically be re-routed to the login page). Traffic between

the user's PC and the server is secured using standard SSL (Secure Sockets Layer) technology.

Unattended PCs left logged onto the system will 'timeout' after a period to be agreed with trial

management (30 minutes by default).

f) One-to-one sessions will be provided to enable more private discussions with DPFs if requested.

g) For focus groups and interviews, transcriptions will be labelled by code number only and stored securely; electronically on a password protected secure system and hard copy in a locked filing cabinet in the CRTU at the UEA. Where participant quotes are used as research data, these will be fully anonymised.

Good Clinical Practice and Research Ethics Committee (GCP and REC)

a) All members of the research team will receive GCP training. The DPMs will be formally mentored by the Senior Intervention DPM RA.

b) Participants are informed in the Participant Information Sheet that they are entitled to leave the study at any time without giving a reason.

c) In the event of any amendments being made (and approved by REC and all relevant R+D offices) to this document the new amended version including new version number and amended date, will be distributed to any registered participating sites. All amendments will be submitted for NIHR, REC and R+D approval before being implemented.

d) Compliance with this protocol will be in accordance with the (REC) approval, NHS Research Governance, (GCP) and the Data Protection Act.

e) The Norfolk DPS has been registered with UKCRN for portfolio inclusion.

INDEMNITY

Indemnity is provided through the sponsor, Norfolk and Norwich University Hospitals NHS Foundation Trust.

FINANCE

Funded through the National Institute for Health Research.

PUBLICATION STRATEGY

All outputs from this programme will be submitted to peer review journals and no participant identifiable materials will be used

APPENDICES

Appendix 1 Appendix 2 NNUH Risk assessment form

Appendix 3 Example of Database Search Instruction Manual

Appendix 4 Diabetes Pathway

Appendix 5 OGTT protocol

Appendix 6 Screening protocol

Appendix 7 Committee and sub group structure Diagram

Appendix 8 Staff Line Management Diagram

Appendix 9 Vascular Screening Pathway

Appenidx 10 Method and timepoints of collection/analysis for screening

tests and measurements

Appendix 1. GANNT Chart for the Norfolk DPS

| Appendix 2 - NNUH Risk Assessment | | | | | | | | | | | | | | | | | | | | | | | | | | | | | | | | | | | | | | | |
| --- | --- | --- | --- | --- | --- | --- | --- | --- | --- | --- | --- | --- | --- | --- | --- | --- | --- | --- | --- | --- | --- | --- | --- | --- | --- | --- | --- | --- | --- | --- | --- | --- | --- | --- | --- | --- | --- | --- | --- |
| NORFOLK AND NORWICH UNIVERSITY HOSPITAL - RISK ASSESSMENT FORM | | | | | | | | | | | | | | | | | | | | | | | | | | | | | | | | | | | | | | | |
| Ward / Department: | |  | | | | | | | | | | | | | | | | | | | | | | | | | Date of Assessment: | | | |  | | | | | | | | |
| Category of person(s) affected: | | | | | | | Staff | | | |  | | | Patients |  | | | | | Visitors | | | | |  | | Contractors | | |  | Other | | | | | |  | | |
| Number of persons affected: (Tick Box) | | | | | | | 1 | | | |  | | Up to 10 | | |  | | | | 10 to 50 | | | | |  | | 50 to 100 | | |  | Trust Wide | | | | | |  | | |
| Risk Assessment of: |  | | | | | | | | | | | | | | | | | | | | | | | | | | | | | | | | | | | | | | |
| Category of Risk: | ENVIRONMENTAL | | | |  | EQUIPMENT | | | | | |  | SUBSTANCES | | | |  | HUMAN FACTORS | | | | | |  | | BIOLOGICAL | |  | ERGONOMIC incl. MANUAL HANDLING | | | | |  | | CLINICAL | | |  |
| SECTION A | | | | | | | | | SECTION B | | | | | | | | | | | | | | | | | SECTION C | | | | | | | | | | | | Date Action to be reviewed or date completed | |
| Description of Hazard.  L x C =RR | | | | | | | | | Existing Controls and Workplace Precautions in place.  L x C =RR | | | | | | | | | | | | | | | | | Recommendation and / or Action Required to further reduce or eliminate the risk. L x C =RR | | | | | | | | | | | |  |  |
| Ref. No. | | | L | C | | | | RR |  | | | | | | | | | | L | | C | | RR | | |  | | | | | | L | C | | RR | | |  |  |
|  | | |  |  | | | |  |  |  |  |  |  |  |  |  |  |  |  | |  | |  | | |  |  |  |  |  |  |  |  | |  | | |  | |
| Management Comments: | | | | | | | | | | | | | | | | | | | | | | | | | | | | | | | | | | | | | | | |
|  |  |  |  |  |  |  |  |  |  |  |  |  |  |  |  |  |  |  |  |  |  |  |  |  |  |  |  |  |  |  |  |  |  |  |  |  |  |  |  |
| Risk Assessment Completed by: | | | | | | | | | |  | | | | | | | | | | | | Signed: | | | | | | | | | | | | | | | | | |
| Service Manager/Head of Department : | | | | | | | | | |  | | | | | | | | | | | | Signed: | | | | | | | | | | | | | | | | | |

| L = Likelihood of event occurring | Rare =1 | Unlikely = 2 | Possible =3 | Likely = 4 | Almost certain = 5 |
| --- | --- | --- | --- | --- | --- |
| C = Consequences of event occurring | Insignificant =1 | Minor = 2 | Moderate =3 | Major = 4 | Catastrophic = 5 |
| RR = Risk Rating |  |  |  |  |  |

Appendix 3 - Example of GP Practice database search from feasibility study

Example of Vision search for UEA-IFG Study

Search on: permanent and applied

Date of birth age range from 45 – 70, inclusive

This seems to be possible by adding an entity and having this in a range format using: After T-70 and before T-44

Exclude from the age range all those with diabetes (type 1 or type 2):

Exclude all CI0 (or those on the diabetic register)

Run this search and save it. This search is the original working cohort. All the other searches to be conducted as separate individual searches.

BMI > 25

Using the original cohort, this depends a little on where the information has been entered. E.g. most people will be weighed and their height measured, but the system automatically works out the BMI, so it is not entered as a specific read code.

Suggest, Add entity, weight/BMI then search in the range >25.

Save and print this search. Clear search, this is search 1.

Fasting plasma glucose

Using the original cohort

Remove the BMI search criteria

Add entity, biochemistry, fasting glucose, end of normal range, range = 6.1-7

It may be necessary to untick the hierarchy box for these boxes as you are searching on a specific range rather than the specific read codes.

Save and print this search. Clear search, this is search 2.

Family history of diabetes

Using the original cohort

Remove fasting glucose search criteria

Add entity/family history/endocrine/diabetes

Possible read codes 1252/1253

Save and print this search. Clear search, this is search 3.

Personal history of ischaemic heart disease

Using the original cohort

Remove family history search criteria

Search all those on heart disease register

Or possible read code G3… IHD

Save and print this search. Clear search, this is search 4.

Gestational diabetes

Using the original cohort

Remove the heart disease search criteria

Add entity other clinical data

Possible read code L1808 or L180811 depending on how it has been entered

Save and print this search. Clear search, this is search 5

Add together totals from searches 1-5

Search 6 should remove those patients with a BMI > 25 - 26.9

This is the total for mail shot

Check the list manually to exclude duplicate entries

Check the list for additional exclusions, e.g. learning difficulties, malignancies, social reasons for exclusion by the practitioner.

The final list is the number of patients suitable to enter into our study.

We will prepare packs for this number; we ask that you have the mail merged letters ready for us to add to the pack at visit 2.

The practice needs to provide stamps/postage and then invoice Heather Leishman for reimbursement. We will aim to post the packs the same day.

As a guide, for a practice population size of 7,500, the number of packs would be in the region of 700.


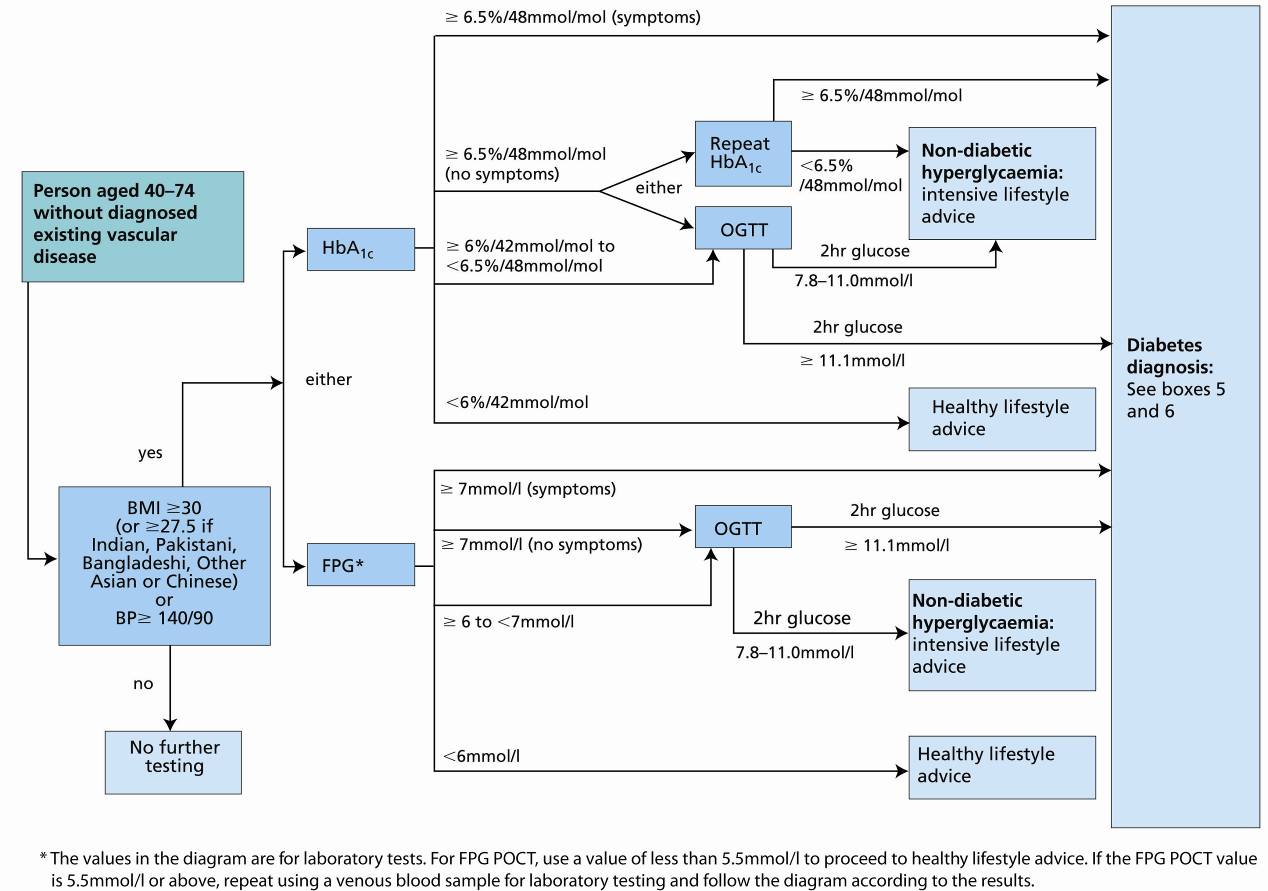
Appendix 4 - Diabetes Pathway

Appendix 5 - Norfolk DPS Oral Glucose Tolerance Test Protocol

Oral Glucose Tolerance Test (OGTT)

Dose: 75 grams of anhydrous glucose (82.5 grams of glucose monohydrate).

Obtained as: a pre-weighed powder from NNUH pharmacy. Supplied in: sealed white plastic tubs with standard pharmacy labelling.

Storage: Store tubs of glucose in locked drugs cupboard at CRTU, UEA (room temperature).

Retain a log of supply within CRF.

Participant Instructions: Participant sent information leaflet instructing: i) consumption of a normal diet 3 days prior to test; ii) fasting from 10 pm the previous evening (except sips of water); iii) the need to stay at the unit for 2.5 hours for the test.

Procedure for diabetes test (OGTT)

Record procedure in participant’s CRF including: batch number and expiry date of anhydrous glucose and signature of person administering dose.

*Preparation of dose*: dissolve 75 grams of anhydrous glucose (82.5 grams of glucose monohydrate) in a small amount of warm water to dissolve the powder and then make up to 150 mls with cold water.

*Check participant has fasted:* The participant must have fasted from 10pm the night before the appointment (sips of water only).

Perform venepuncture and take 2mls of blood; test a small sample using a Medisense Optium Meter. Place the remainder of the blood sample into fluoride oxalate (grey cap) blood tube; label with patient identification; date and time: ‘0’ minutes.

If result on Medisense Optium Meter is greater than or equal to 11mmol/l do not continue with OGTT.

Sample Time

Two hours after dose given: collect blood sample in fluoride oxalate (grey cap) blood tube. Label tube with: patient identification, date and time ‘120’ minutes sample.

Completion of blood tube label/form:

Ensure blood form and tubes are correctly labelled with: participant ID; Date of Birth (DoB); time of sample and ‘0’ or ‘120’ minutes; OGTT test and time of test; signature of person taking blood sample.

Use the same blood form for both samples and send both samples and form to lab together.

Information to participants:

The participant should be advised that they will receive their results by letter as soon as possible and that a copy will be sent to their GP.

Remind participants to take any prescribed medication they may have missed due to fasting and advise where they can obtain refreshments.

Give details of a contact if they have any concerns or further questions. Thank them for their time and for taking part in the trial.

Recording information

Details of the test should be recorded accurately and legibly using a black, ball point pen in the participant’s confidential file and on the trial database. Cross out any changes or corrections using a single line that does not obscure the original entry and initial and date. Include: any untoward events such as faints or bruising. All documents should be stored in the trial office within a locked cabinet.

*Minimising risks to participant:* Record and note any:

1. Given history of problems during blood tests e.g. fainting/ bruising and offer option to lie down.

2. Avoid the use of plasters/micropore/transpore tape if participant states allergic.

3. Medication that would interfere with blood clotting, i.e. aspirin or warfarin: apply prolonged direct

pressure to the venepuncture site until bleeding ceases.

Source Document for OGTT protocol : NNUHFT Trust Protocol for the Performance of Dynamic Endocrine Function Tests in Adults (CIU 3 version 2 April 2009)

Appendix 6 - Norfolk DPS Oral Glucose Tolerance Test Protocol

SCREENING PROTOCOL

Preparation

Participant sent information leaflet instructing: fasting from 10 pm the night before the screening appointment (except sips of water).

Procedure

Conducted by Senior Health Care Assistants/trained phlebotomists.

Use CRF to record procedure direct to trial database.

Participant eligibility for screening checked.

Screening procedure explained to participant and any questions answered.

Informed written consent obtained for screening

One copy of the signed consent form will be retained for the TMF and a copy given to the participant to keep. If the participant declines to provide written consent for the screening they will be excluded.

1. Measure and record: Height; weight; body fat mass; BMI; waist circumference and, if following

a first screening take blood pressure (in accordance with Norfolk-DPS SOPs 3 and 4).

2. Take a blood sample (in accordance with NNUHFT policy for venepuncture) to determine:

- Fasted Plasma Glucose (FPG);
- HbA1c

This should take approximately 10minutes.

At second and subsequent screenings; fasting lipids and plasma insulin will also be checked.

(10 minutes).

3. Give participants business cards to distribute to family and friends (voluntary).

4. Advise participant that they will receive their results by letter as soon as possible after

the blood test and that a copy will be sent to their GP.

5. Remind participants to take any prescribed medication they may have missed due to fasting and

advise where they can obtain refreshments.

6. Give details of person whom they can contact if they have any concerns or further questions.

Thank them for their time and for taking part in the trial.

Samples

Samples must be sent for laboratory testing on the morning of taking as whole blood.

Blood samples must be in the correct tubes (FPG -grey cap; HbA1c - lavender cap; lipids and insulin - gold cap).

Blood tubes must be labelled with: participant details, date and time of sample and signature of phlebotomist.

Recording Information

The screening must be: accurately recorded; legible; written in black ink; recorded in participant CRF and programme database and include any untoward events such as faints or bruising. Any changes or corrections should be crossed through using a single line that does not obscure the original entry. The correction should then be dated and initialled.

Storage

All documents should be stored in the Norfolk DPS office within a locked cabinet.

*Detail of Medications Taken and Medications Not Permitted/ Precautions*

Prior to recording body fat analysis, confirm that the participant is not fitted with a cardiac pacemaker or other internal bioelectric device. This is an absolute contra-indication to undertaking body fat analysis.

*Minimising risks to participants:*

1. Record and note any given history of problems during blood tests e.g. fainting/ bruising and offer

option to lie down.

2. Avoid plasters/ micropore if participant states allergic.

3. Medication that would interfere with blood clotting, i.e. aspirin or warfarin: apply prolonged direct

pressure to the venepuncture site until bleeding ceases.

**Appendix 7 - Norfolk DPS Committee and Sub Group Structure**

**Strategic Trials Committee (STC)**

Chair

**Data Monitoring Committee (DMC):**

Chair

CI

Chair: Professor Mike Sampson

**Intervention**

**DPM RA (NM)**

**DPF RA (SA)**

**MJS**

**MP**

DPFs

physical activity facilitator

Amanda Howe

Alison Woodcock

Colin Greaves

Imogen Hobbis

Jane Smith

Clare Bradley

Shirley Reynolds

Admin

**Screening**

**RA**

**MJS**

**MP**

HCAs

Louise Jones (DRN)

Ketan Dhatariya

Tara Wallace

Garry John

Jeremy Turner

Jane Ewing

Admin

**Health Psych**

**DPM RA (NM)**

**MJS**

**MP**

Colin Greaves

Alison Woodcock

Claire Bradley

Falko Sneihotta

Jane Smith

**Health Economics**

**Garry Barton**

**MJS**

**MP**

Lisa Irvine

Darren Burns

Max Bachmann

**PAB**

**Dave Rea**

**MJS**

**MP**

Karen Warrington

DPM Champion

**Qualitative**

**Amanda Howe**

**MJS**

**MP**

Claire Bradley

DPFs

**Co-investigators**

**MJS**

**MP**

Max Bachmann

Dave Rea

Allan Clark

Tara Wallace

Amanda Howe

Jane Smith

Imogen Hobbis

Garry John

Ketan Dhataryia

Ian Harvey

Garry Barton

Plus RAs

Appendix 7 – Key Norfolk DPS Committee and Sub Group Structure

**KEY**

Admin Administration team

CI Chief Investigator

PI Principal Investigator

DMC Data Monitoring Committee

DPF Diabetes Prevention Facilitator

DPM Diabetes Prevention Mentor

DRN Diabetes Research Network

Mgr Manager

MJS Prof Mike J Sampson (CI)

MP Dr Melanie Pascale *(nee Dunk)*

Norfolk DPS Norfolk Diabetes Prevention Study

PAB Participant Advisory Board

Pract. Practitioner

Psych Psychology

RA Research Associate

STC Strategic Trials Committee

**DPFs**

Rebbecca Semence

Nicky Stroud

**Elysia Young**

**Senior Health Care Assistants**

Debbie Thompson

Sarah Wilford

**DPMs (Volunteers)**

**Screening Research Facilitator**

Clare Ferns

**Senior Intervention RA (DPF)**

Dr Sara Auckland

**Senior Intervention RA (DPM)**

Kalman France

**Fitness Facilitator**

James Kennedy

Nicky Stroud

**Senior Programme Manager & Principal Investigator**

Dr Melanie Pascale *(nee) Dunk)*

**Health Economics RA**

Dr Garry Barton

**Senior Research Administrator/PA to Melanie Pascale**

Karen Warrington

**Chief Investigator & Consultant Endocrinologist**: Professor Mike Sampson

**Appendix 8 - Norfolk DPS - Staff Line Management Structure**

**Clerical assistant**

TBC

**Clerical assistant**

Elaine Falconbridge

**Research Assistant**

Lee Toney

**CRN Senior Research Nurse Louise Jones**

**TBC**

**Lead DPF**

Rebecca Usher

**Volunteers**

Appendix 9 - National Vascular Screening Pathway


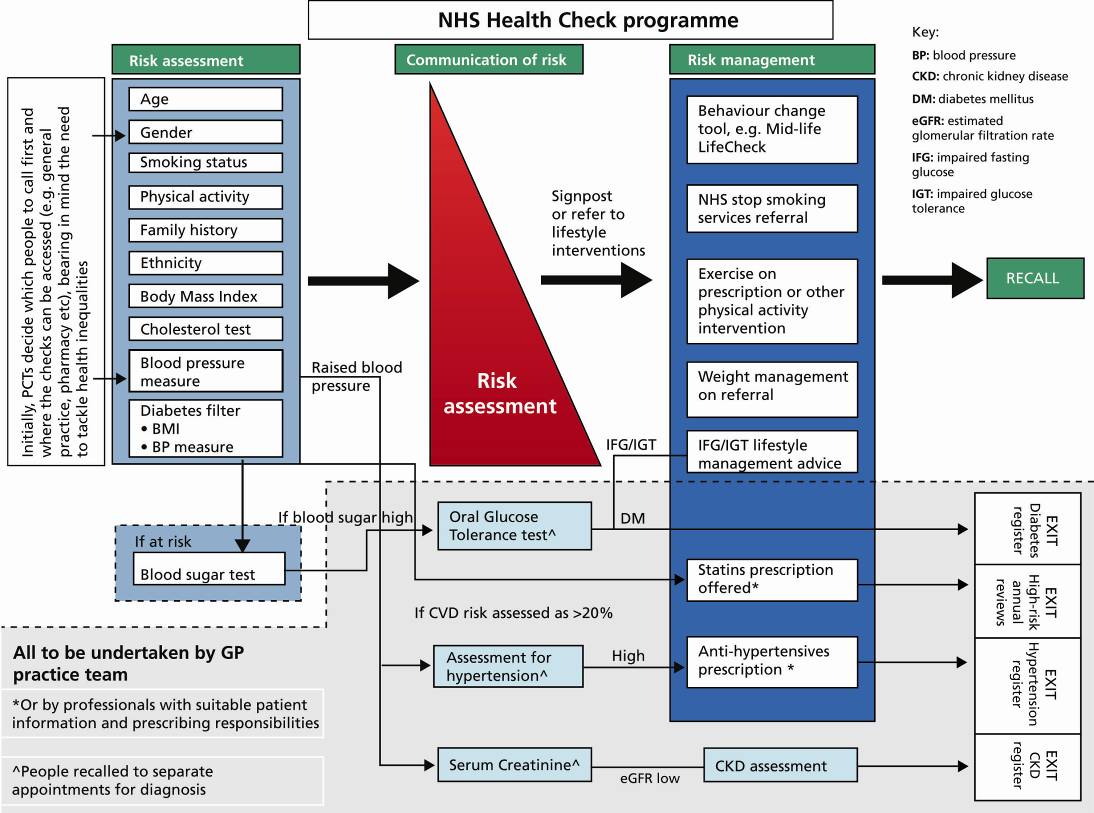


Appendix 10: Method and time points of collection/analysis for the screening tests and

Measurements

| Test | First Screening | Subsequent Screenings | Method |
| --- | --- | --- | --- |
| Fasting Plasma Glucose | Yes | Yes | Enzymatic hexokinase |
| HbA1c | Yes | No | Cation exchange HPLC (Menarini Diagnostics HA8160 analyser) |
| Fasting Lipids | No | Yes | Cholesterol and Triglyceride and LDL measured using enzymatic methods; HDL utilising detergent and enzymatic cholesterol method |
| Plasma Insulin | No | Yes | Immunoassay (DPL) |
| OGTT | No | Yes, if IFG ≥ 6.1 to <7.0 mmol/l) and an HbA1c  ≥6.0%(42 mmols/l) | Enzymatic hexokinase |
| BMI | Yes | No | Weight (Kg) divided by height (m) squared |
| Blood pressure | No | Yes | BHS approved automated instruments (Omron BPM) |
| Waist circumference | Yes | No | Standard tape measure (cm) halfway between the lowest point of ribcage and the anterior superior iliac crests when standing |
| Hip measurement | Yes | No | Standard tape measure (cm) Widest part of hip |
| Body fat mass | Yes | No | Bio-impedance (Tanita BC-420MA analyser, Tokyo, Japan) |
| Visceral fat | Yes | No | Bio-impedance (Tanita BC-420MA analyser, Tokyo, Japan) |
| Fat percentage | Yes | No | Bio-impedance (Tanita BC-420MA analyser, Tokyo, Japan) |

.

References:

1. DH. About Diabetes. 2009.

2. DH. Diabetes National Service Framework : standards. *Health Service circular*: NHS Executive, 2001.

3. NICE. Type 2 Diabetes National clinical guideline for management

in primary and secondary care (update). The Royal College of Physicians: London, 2008.

4. Diabetes U. K. Key Statistics on Diabetes. Diabetes U.K: London, 2009.

5. Wexler DJ, Grant RW, Wittenberg E, et al. Correlates of health-related quality of life in type 2 diabetes. *Diabetologia*. 2006; 49: 1489-97.

6. Williams R, Van Gaal L, Lucioni C. Assessing the impact of complications on the costs of Type II diabetes. *Diabetologia*. 2002; 45: S13-7.

7. Koopmanschap M. Coping with Type II diabetes: the patient's perspective. *Diabetologia*. 2002; 45: S18-22.

8. Currie CJ, Peters JR. The demand and financial cost of hospital care for diabetes mellitus and its related complications. *Diabet Med*. 1998; 15: 449-51.

9. Sampson MJ, Crowle T, Dhatariya K, et al. Trends in bed occupancy for inpatients with diabetes before and after the introduction of a diabetes inpatient specialist nurse service. *Diabet Med*. 2006; 23: 1008-15.

10. Forouhi NG, Balkau B, Borch-Johnsen K, et al. The threshold for diagnosing impaired fasting glucose: a position statement by the European Diabetes Epidemiology Group. *Diabetologia*. 2006; 49: 822-7.

11. Davidson MB. Counterpoint: the oral glucose tolerance test is superfluous. *Diabetes Care*. 2002; 25: 1883-5.

12. Faerch K, Vaag A, Holst JJ, Hansen T, Jorgensen T, Borch-Johnsen K. Natural history of insulin sensitivity and insulin secretion in the progression from normal glucose tolerance to impaired fasting glycemia and impaired glucose tolerance: the Inter99 study. *Diabetes Care*. 2009; 32: 439-44.

13. Faerch K, Borch-Johnsen K, Holst JJ, Vaag A. Pathophysiology and aetiology of impaired fasting glycaemia and impaired glucose tolerance: does it matter for prevention and treatment of type 2 diabetes? *Diabetologia*. 2009.

14. Tai ES, Lim SC, Tan BY, Chew SK, Heng D, Tan CE. Screening for diabetes mellitus--a two-step approach in individuals with impaired fasting glucose improves detection of those at risk of complications. *Diabet Med*. 2000; 17: 771-5.

15. Valdes S, Botas P, Delgado E, Alvarez F, Cadorniga FD. Does the new American Diabetes Association definition for impaired fasting glucose improve its ability to predict type 2 diabetes mellitus in Spanish persons? The Asturias Study. *Metabolism*. 2008; 57: 399-403.

16. Gillies CL, Abrams KR, Lambert PC, et al. Pharmacological and lifestyle interventions to prevent or delay type 2 diabetes in people with impaired glucose tolerance: systematic review and meta-analysis. *BMJ*. 2007; 334: 299.

17. Yamaoka K, Tango T. Efficacy of lifestyle education to prevent type 2 diabetes: a meta-analysis of randomized controlled trials. *Diabetes Care*. 2005; 28: 2780-6.

18. Knowler WC, Barrett-Connor E, Fowler SE, et al. Reduction in the incidence of type 2 diabetes with lifestyle intervention or metformin. *N Engl J Med*. 2002; 346: 393-403.

19. DPP. The Diabetes Prevention Program (DPP): description of lifestyle intervention. *Diabetes Care*. 2002; 25: 2165-71.

20. DPP. Within trial cost effectiveness of lifestyle intervention or metformin for the primary prevention of type 2 diabetes. *Diabetes Care*. 2003.

21. Crandall JP, Knowler WC, Kahn SE, et al. The prevention of type 2 diabetes. *Nat Clin Pract Endocrinol Metab*. 2008; 4: 382-93.

22. Schwarz PE, Gruhl U, Bornstein SR, Landgraf R, Hall M, Tuomilehto J. The European perspective on diabetes prevention: development and Implementation of A European Guideline and training standards for diabetes prevention (IMAGE). *Diab Vasc Dis Res*. 2007; 4: 353-7.

23. Active in Diabetes Prevention. 2009.

24. Baksi AK. Experiences in peer-to-peer training in diabetes mellitus: challenges and implications. *Fam Pract*. 2009.

25. Wanless D. Securing our Future Health Taking a Long Term Veiw. In: Treasury H, (ed.): HM Treasury, 2002, p. 14.

26. Dorgo S, Robinson KM, Bader J. The effectiveness of a peer-mentored older adult fitness program on perceived physical, mental, and social function. *J Am Acad Nurse Pract*. 2009; 21: 116-22.

27. Kennedy A, Reeves D, Bower P, et al. The effectiveness and cost effectiveness of a national lay-led self care support programme for patients with long-term conditions: a pragmatic randomised controlled trial. *J Epidemiol Community Health*. 2007; 61: 254-61.

28. Kennedy LA, Milton B, Bundred P. Lay food and health worker involvement in community nutrition and dietetics in England: roles, responsibilities and relationship with professionals. *J Hum Nutr Diet*. 2008; 21: 210-24.

29. Rasmussen SS, Glumer C, Sandbaek A, Lauritzen T, Borch-Johnsen K. Progression from impaired fasting glucose and impaired glucose tolerance to diabetes in a high-risk screening programme in general practice: the ADDITION Study, Denmark. *Diabetologia*. 2007; 50: 293-7.

30. Engberg S, Vistisen D, Lau C, et al. Progression to impaired glucose regulation and diabetes in the population-based Inter99 study. *Diabetes Care*. 2009; 32: 606-11.

31. Paulweber B, Valensi P, Lindstrom J, et al. A European evidence-based guideline for the prevention of type 2 diabetes. *Horm Metab Res*. 42 Suppl 1: S3-36.

32. Lindstrom J, Neumann A, Sheppard KE, et al. Take action to prevent diabetes--the IMAGE toolkit for the prevention of type 2 diabetes in Europe. *Horm Metab Res*. 42 Suppl 1: S37-55.

33. Hardeman W, Griffin S, Johnston M, Kinmonth AL, Wareham NJ. Interventions to prevent weight gain: a systematic review of psychological models and behaviour change methods. *Int J Obes Relat Metab Disord*. 2000; 24: 131-43.

34. Laatikainen T, Dunbar JA, Chapman A, et al. Prevention of type 2 diabetes by lifestyle intervention in an Australian primary health care setting: Greater Green Triangle (GGT) Diabetes Prevention Project. *BMC Public Health*. 2007; 7: 249.

35. Absetz P, Oldenburg B, Hankonen N, et al. Type 2 diabetes prevention in the real world: three-year results of the GOAL lifestyle implementation trial. *Diabetes Care*. 2009; 32: 1418-20.

36. Schwarzer R. *Self-efficacy:Thought control of action*. Washington DC1992.

37. Yates T, Davies M, Gorely T, Bull F, Khunti K. Rationale, design and baseline data from the Pre-diabetes Risk Education and Physical Activity Recommendation and Encouragement (PREPARE) programme study: a randomized controlled trial. *Patient Educ Couns*. 2008; 73: 264-71.

38. Gollwitzer PMS, P. Implementation intentions and goal achievement: A meta-analysis of effects and processes. . *Advances in Experimental Social Psychology* 2006; 38: 69-119.

39. DH. Improving Health: Changing Behaviour: NHS Health Trainer Handbook. 2008.

40. Hobbis IC, Sutton S. Are techniques used in cognitive behaviour therapy applicable to behaviour change interventions based on the theory of planned behaviour? *J Health Psychol*. 2005; 10: 7-18; discussion 37-43.

41. Funnell MM, Anderson RM. Empowerment and Self-Management of Diabetes. *Clinical Diabetes*. 2004; 22: 123-7.

42. Funnell M, Weiss M. Patient empowerment, the LIFE approach. *EDN Summer*. 5: 75-8.

43. Hammersley MS, Meyer LC, Morris RJ, Manley SE, Turner RC, Holman RR. The Fasting Hyperglycaemia Study: I. Subject identification and recruitment for a non-insulin-dependent diabetes prevention trial. *Metabolism*. 1997; 46: 44-9.

44. DH. Putting Prevention First. NHS Healthcheck Vascular Risk Assessment and Management Best Practice Guidance. Department of Health: London, 2009.

45. Anekwe L. Analysis reveals huge workload implications of vascular screening. *PULSE*. 2009.

46. Is fasting glucose sufficient to define diabetes? Epidemiological data from 20 European studies. The DECODE-study group. European Diabetes Epidemiology Group. Diabetes Epidemiology: Collaborative analysis of Diagnostic Criteria in Europe. *Diabetologia*. 1999; 42: 647-54.

47. NHS. Your Weight Your Health. Department of Health: London, 2006.

48. ERPHO. Maps and Atlases.

49. SACN. The Nutritional Wellbeing of the British Population. The Stationary Office: London, 2008.

50. Craig P, Dieppe P, Macintyre S, Michie S, Nazareth I, Petticrew M. Developing and evaluating complex interventions: the new Medical Research Council guidance. *BMJ*. 2008; 337: a1655.

51. Lindstrom J, Ilanne-Parikka P, Peltonen M, et al. Sustained reduction in the incidence of type 2 diabetes by lifestyle intervention: follow-up of the Finnish Diabetes Prevention Study. *Lancet*. 2006; 368: 1673-9.

52. de Vegt F, Dekker JM, Jager A, et al. Relation of impaired fasting and postload glucose with incident type 2 diabetes in a Dutch population: The Hoorn Study. *JAMA*. 2001; 285: 2109-13.

53. Sterne JA, White IR, Carlin JB, et al. Multiple imputation for missing data in epidemiological and clinical research: potential and pitfalls. *BMJ*. 2009; 338: b2393.

54. Brooks R. EuroQol: the current state of play. *Health Policy*. 1996; 37: 53-72.

55. NICE. Guide to the Methods of Technology Appraisal. *NICE publications*: National Institute of Health and Clinical Excellence, 2008.

56. Heijden vA, Ortegon M, Niessen LW, Nijpels G, Dekker JM. Prediction of coronary heart disease in a general pre-diabetic and diabetic population during 10 years of follow-up: accuracy of the Framingham, SCORE and UKPDS risk functions- the Hoorn Study. *Diabetes Care*. 2009.

57. Heisler M. Overview of Peer Support Models to Improve Diabetes Self-Management and Clinical Outcomes. *Diabetes Spectrum*. 2007; Volume 20.

58. Griffin S, Kinmonth AL. Diabetes care: the effectiveness of systems for routine surveillance for people with diabetes. *Cochrane Database Syst Rev*. 2000: CD000541.

59. Spijkerman A, Griffin S, Dekker J, Nijpels G, Wareham NJ. What is the risk of mortality for people who are screen positive in a diabetes screening programme but who do not have diabetes on biochemical testing? Diabetes screening programmes from a public health perspective. *J Med Screen*. 2002; 9: 187-90.

1. This summary has been written in part using the wording in the grant proposal submission which was written in consultation with the Patient and Public Involvement In Research group (PPiRes) [↑](#footnote-ref-1)
