# Supplementary material for: Effects of the Norfolk diabetes prevention lifestyle intervention (NDPS) on glycaemic control in screen-detected type 2 diabetes: a randomised controlled trial
Source: BMC Med. 2021 Aug 19;19:183. doi: 10.1186/s12916-021-02053-x (PMC8375190; doi:10.1186/s12916-021-02053-x)
Supplement: Supplementary file 4 — Additional file 4. Additional data Tables A and B. [file 12916_2021_2053_MOESM4_ESM.docx]

**Additional Table A. Descriptive data for outcomes at 12 months by trial arm**

|  | **CON** | n | **INT** | n | **INT- DPM** | n |
| --- | --- | --- | --- | --- | --- | --- |
| **n** | 119 |  | 100 |  | 97 |  |
| **HbA1c (mmol/mol)** | 48.5 (9.1) | 119 | 46.5 (8.1) | 100 | 45.6 (6.0) | 97 |
| **HbA1c < 58 mmol/mol, n (%)** | 109 (91.6%) | 119 | 94 (94.0%) | 100 | 93 (95.9%) | 97 |
| **Fasting plasma glucose (mmol/l)** | 6.7 (1.4 ) | 119 | 6.7 (1.8) | 100 | 6.5 (1.1) | 97 |
| **Weight (kg)** | 88.3 (19.19) | 119 | 86.1 (15.9) | 100 | 87.9 (17.5 ) | 96 |
| **Body mass index (kg/m^2^ )** | 30.54 (6.2 ) | 119 | 30.27 (5.2 ) | 100 | 30.95 (5.5 ) | 96 |
| **Body fat (%) ^a^** | 34.6 (9.8 ) | 114 | 35.3 (9.4 ) | 95 | 36.0 (8.9) | 94 |
| **Body fat mass (kg) ^a^** | 31.59 (14.1) | 114 | 31.04 (11.76) | 95 | 32.13 (12.63) | 94 |
| **Visceral fat score ^a^** | 14.5 (5.3) | 114 | 14.4 (4.0) | 95 | 14.7 (5.5) | 94 |
| **Waist circumference (cm)** | 103.8 (14.4) | 118 | 102.7 (12.2 ) | 100 | 103.90 (13.1 ) | 96 |
| **Physical activity :**  **MET mins / week ^b^** | 3543 (3998) | 81 | 3608 (3065) | 67 | 2877 (2356) | 45 |
| **Low Physical activity category, n (%) ^b^** | 15 (19%) | 81 | 10 (15%) | 67 | 9 (20%) | 45 |
| **Physical activity : Minutes sitting/week ^b^** | 417 (253) | 85 | 389 (259) | 68 | 458 (273) | 51 |
| **Fat scale score ^c^** | 2.39 (0.36) | 94 | 2.33 (0.34) | 79 | 2.50 (0.37) | 55 |
| **Fibre scale score ^c^** | 2.47 (0.40) | 94 | 2.48 (0.37) | 79 | 2.47 (0.44) | 55 |
| **W - BQ12 ^d^** | 26.7 (5.5 ) | 89 | 26.4 (5.6) | 74 | 26.4 (5.7) | 55 |
| **EQ – 5D ^d^** | 0.82 (0.25) | 93 | 0.82 (0.22) | 77 | 0.84 (0.18) | 56 |
| **DTSQ ^d^** | 30.4 (5.5) | 65 | 28.7 (6.4) | 60 | 28.8 (7.8) | 44 |
| **DMSES ^d^** | 115.6 (21.9) | 49 | 119.0 (24.2) | 40 | 102.9 (25.4) | 28 |
| **ADDQoL ^d^** | 1.61 (0.86) | 73 | 1.62 (0.93) | 62 | 1.61 (0.87) | 44 |
| **HOMA – B, mean (SD), % ^e^** | 74.85 (37.36) | 115 | 83.26 (50.08) | 96 | 81.04 (35.13) | 93 |
| **HOMA – S, mean (SD), % ^e^** | 90.20 (59.41) | 115 | 81.96 (50.60) | 96 | 80.08 (58.18) | 93 |

Data shown as mean and one standard deviation or n (%), with n showing data available for each variable. ^a^ Fat mass by Tanita body composition analyser. ^b^ Physical activity scales derived from international physical activity questionnaire IPAQ ^c^ Dietary fat and fibre scores based on self-reported Diet Behaviour Questionnaire (DBQ). ^d^ Well - being score (WBQ-12) questionnaire , health related quality of life score (EQ-5D) questionnaire and ADDQoL, diabetes treatment satisfaction questionnaire (DTSQ) , and diabetes self - management efficacy scale (DMSES) . ^e^ Homeostasis model assessment (HOMA) of baseline insulin sensitivity (S) and beta cell function (B) as % of standard reference range.

**Additional Table B Descriptive data for outcomes at 24 months by trial arm**

| **Analysis** | **CON** | **n** | **INT** | **n** | **INT- DPM** | **n** |
| --- | --- | --- | --- | --- | --- | --- |
| **n** | 80 |  | 63 |  | 56 |  |
| **HbA1c (mmol/mol)** | 49.6 (8.6) | 80 | 47.9 (7.3 ) | 63 | 47.7 (7.6) | 56 |
| **HbA1c < 58 mmol/mol, n (%)** | 71 (89%) | 80 | 56 (89%) | 63 | 52 (93%) | 56 |
| **Fasting plasma glucose (mmol/l)** | 6.8 (1.4) | 79 | 6.9 (1.2) | 63 | 6.7 (1.2 ) | 56 |
| **Weight (kg)** | 91.7 (20.5) | 78 | 88.5 (17.5 ) | 63 | 89.3 (18.3 ) | 56 |
| **Body mass index (kg/m^2^)** | 31.7 (7.6) | 78 | 31.3 (5.61) | 63 | 31.2 (5.6) | 56 |
| **Body fat ( %) ^a^** | 33.6 (10.4) | 73 | 36.7 (9.5) | 61 | 35.6 (8.6 ) | 54 |
| **Body fat mass (kg) ^a^** | 32.6 (16.3 ) | 74 | 33.1 (12.8) | 61 | 31.5 (10.5) | 54 |
| **Visceral fat score ^a^** | 15.9 (5.6) | 74 | 15.2 (4.7) | 61 | 14.8 (4.3) | 54 |
| **Waist circumference (cm)** | 107.1 (14.4) | 79 | 104.8 (13.4) | 63 | 104.3 (13.3) | 56 |
| **Physical activity : MET mins / week ^b^** | 3934 (3506) | 50 | 3424 (3068) | 48 | 2782 (2527) | 33 |
| **Low Physical activity category, n (%) ^b^** | 8 (16%) | 50 | 9 (20%) | 45 | 3 (10%) | 29 |
| **Physical activity : Minutes sitting/week ^b^** | 444.8 (260.4) | 55 | 438.8 (243.9) | 48 | 346.5 (126.9) | 33 |
| **Fat scale score ^c^** | 2.34 (0.32) | 64 | 2.46 (0.36) | 46 | 2.52 (0.30) | 37 |
| **Fibre scale score ^c^** | 2.45 (0.45) | 64 | 2.51 (0.35) | 45 | 2.52 (0.33) | 37 |
| **W - BQ12 ^d^** | 25.5 (6.1 ) | 60 | 26.2 (5.1) | 45 | 25.5 (6.5 ) | 37 |
| **EQ – 5D ^d^** | 0.83 (0.3) | 62 | 0.85 (0.2) | 44 | 0.82 (0.2 ) | 37 |
| **DTSQ ^d^** | 29.8 (5.6) | 48 | 30.4 (5.7) | 37 | 29.5 (5.2 ) | 24 |
| **DMSES ^d^** | 117.8 (22 ) | 38 | 120.0 (23) | 28 | 110.9 (21 ) | 16 |
| **ADDQoL ^d^** | 1.56 (1.00) | 54 | 1.47 (0.97) | 38 | 1.52 (0.71) | 26 |
| **HOMA – B (%) ^e^** | 84.5 (53.5 ) | 75 | 75.7 (48.1 ) | 60 | 75.6 (44.5) | 54 |
| **HOMA – S (%) ^e^** | 73.8 (40.2) | 75 | 84.4 (60.7) | 60 | 78.7 (34.7) | 54 |

Data shown as mean and one standard deviation or n (%), with n showing data available for each variable. ^a^ Fat mass by Tanita body composition analyser. ^b^ Physical activity scales derived from international physical activity questionnaire IPAQ ^c^ Dietary fat and fibre scores based on self-reported Diet Behaviour Questionnaire (DBQ). ^d^ Well - being score (WBQ-12) questionnaire , health related quality of life score (EQ-5D) questionnaire and ADDQoL, diabetes treatment satisfaction questionnaire (DTSQ) , and diabetes self - management efficacy scale (DMSES) . ^e^ Homeostasis model assessment (HOMA) of baseline insulin sensitivity (S) and beta cell function (B) as % of standard reference range.
