# Supplementary material for: Effects of the Norfolk diabetes prevention lifestyle intervention (NDPS) on glycaemic control in screen-detected type 2 diabetes: a randomised controlled trial
Source: BMC Med. 2021 Aug 19;19:183. doi: 10.1186/s12916-021-02053-x (PMC8375190; doi:10.1186/s12916-021-02053-x)
Supplement: Supplementary file 5 — Additional file 5: Figure 2: Directed acyclic graph (DAG). [file 12916_2021_2053_MOESM5_ESM.docx]

**Supplementary Figure 2. Directed acyclic graph of potential confounding of association between intervention dose and HbA1c, glucose and weight at follow-up (association between weight and HbA1c and glucose at follow-up not modelled)**

**Baseline variables (potential confounders)**

**Intervention dose**

**Weight at 12 and 24 months**

**HbA1c and glucose at 12 and 24 months**
